# Marine Prostanoids with Cytotoxic Activity from Octocoral *Clavularia* spp.

**DOI:** 10.3390/md22050219

**Published:** 2024-05-14

**Authors:** Ming-Ya Cheng, I-Chi Hsu, Shi-Ying Huang, Ya-Ting Chuang, Tzi-Yi Ke, Hsueh-Wei Chang, Tian-Huei Chu, Ching-Yeu Chen, Yuan-Bin Cheng

**Affiliations:** 1 Department of Marine Biotechnology and Resources, National Sun Yat-sen University, Kaohsiung 804201, Taiwan; mina78976@gmail.com (M.-Y.C.); cj96u04@gmail.com (T.-Y.K.); 2 Division of Pharmacy, Zuoying Armed Forces General Hospital, Kaohsiung 813204, Taiwan; u103531014@gap.kmu.edu.tw; 3 College of Ocean Food and Biological Engineering, Jimei University, Xiamen 361021, China; johnhuang@jmu.edu.cn; 4 PhD Program in Life Sciences, Department of Biomedical Science and Environmental Biology, College of Life Science, Kaohsiung Medical University, Kaohsiung 807378, Taiwan; u112851002@gap.kmu.edu.tw (Y.-T.C.); changhw@kmu.edu.tw (H.-W.C.); 5 Center for Cancer Research, Kaohsiung Medical University, Kaohsiung 807378, Taiwan; 6 Department of Medical Research, Kaohsiung Medical University Hospital, Kaohsiung 807378, Taiwan; 7 Medical Laboratory, Medical Education and Research Center, Kaohsiung Armed Forces General Hospital, Kaohsiung 802301, Taiwan; skbboyz0817@gmail.com; 8 Institute of Medical Science and Technology, National Sun Yat-sen University, Kaohsiung 804201, Taiwan; 9 Department of Physical Therapy, Tzu-Hui Institute of Technology, Pingtung 926001, Taiwan; chingyeu1971@yahoo.com.tw; 10 Graduate Institute of Natural Products, College of Pharmacy, Kaohsiung Medical University, Kaohsiung 807378, Taiwan

**Keywords:** prostanoid, *Clavularia* spp., cytotoxicity, Ca9-22

## Abstract

Octocoral of the genus *Clavularia* is a kind of marine invertebrate possessing abundant cytotoxic secondary metabolites, such as prostanoids and dolabellanes. In our continuous natural product study of *C.* spp., two previously undescribed prostanoids [clavulone I-15-one (**1**) and 12-*O*-deacetylclavulone I (**2**)] and eleven known analogs (**3**–**13**) were identified. The structures of these new compounds were elucidated based on analysis of their 1D and 2D NMR, HRESIMS, and IR data. Additionally, all tested prostanoids (**1** and **3**–**13**) showed potent cytotoxic activities against the human oral cancer cell line (Ca9-22). The major compound **3** showed cytotoxic activity against the Ca9-22 cells with the IC_50_ value of 2.11 ± 0.03 μg/mL, which echoes the cytotoxic effect of the coral extract. In addition, in silico tools were used to predict the possible effects of isolated compounds on human tumor cell lines and nitric oxide production, as well as the pharmacological potentials.

## 1. Introduction

Octocoral of the genus *Clavularia* (clove polyps) belongs to the family Clavulariidae, with distribution over subtidal reef flats of the Red Sea, Great Barrier Reef, and Indo-Pacific. The polyps of *Clavularia* show pink, yellow, or green to brownish-gray colors, which are popular in aquariums. Within all *Clavularia* species, four species, namely *C. viridis*, *C. inflata*, *C. kentingsnsis*, and *C. koellikeri*, were found in Taiwan. Among those four species, *C. viridis* and *C. inflata* were investigated as abundant natural product producers, and prostanoids [1], terpenoids [2,3], and steroids [4,5] were identified. To date, more than 60 marine prostanoids have been identified from the genus *Clavularia*. Those marine prostanoids present diverse pharmacological effects, including anti-proliferative [6], cytotoxic [7,8,9], and anti-inflammatory activities [10].

The fruit of *Arecae catechu* Linn (areca nuts), which contains arecoline, is known to be a psychoactive substance. The use of areca nuts as chewing gum is a tradition of people who live in south-central Asia, such as Taiwan and the Philippines. However, this behavior dramatically increases the risk of oral cancer. In 2020, the overall incidence rate of oral cancer was 21.8 per 100,000, which rates fourth among cancer-related deaths of male Taiwanese people. Taiwan has the highest frequency of oral cancer worldwide [11], and there are roughly 3000 fatalities in Taiwan each year. Chemotherapy is one of the typical approaches in the treatment of oral cancer; however, side effects from chemotherapy medications might occur [12]. Hence, finding effective and safe anti-oral cancer medications is essential.

**Figure 1:**
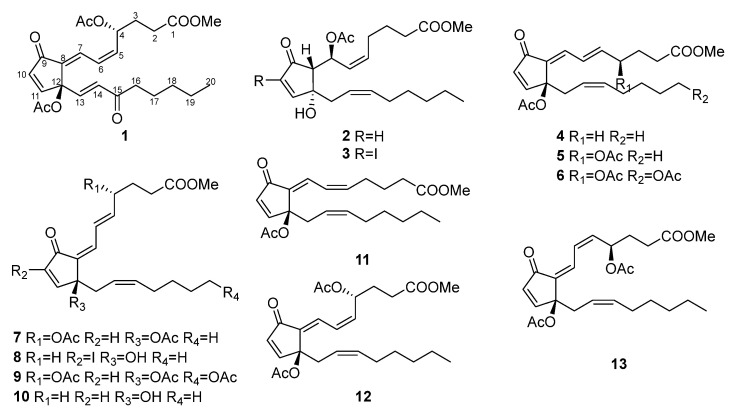
Structures of marine prostanoids **1**–**13** isolated from *Clavularia* spp.

In our continuous research for bioactive natural products from marine invertebrates, we recently reported the isolation and detailed structure elucidation of five new eudensamane-type sesquiterpene lactones and three new dolabellanes from *Clavularia* spp. [3]. We now report the isolation and structure elucidation of two new and eleven known prostanoids (**1**–**13**) from the same specimen (Figure 1). Moreover, the cytotoxic effects of isolated compounds were evaluated. We also used in silico tools to predict the possible effects of thirteen compounds on human tumor cell lines (for cytotoxicity) and the nitric oxide (NO) production in macrophages, and predict the pharmacological potentials of nine compounds (**1**–**5**, **7**, **8**, **10**, and **11**) (the other four compounds were not suitable for the in silico tool).

## 2. Results

### 2.1. Structure Elucidation of Isolated Compounds

**Figure 2:**
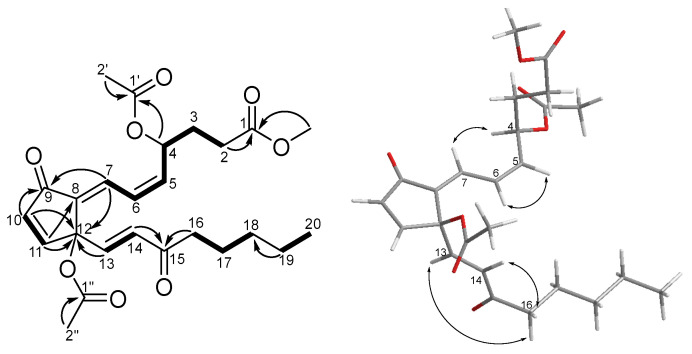
Key COSY (bold), HMBC (arrow), and NOESY (double arrow) correlations of **1**.

Clavulone I-15-one (**1**) was obtained as a colorless oil and had the molecular formula C_25_H_32_O_8_ (Δ = 9) based on its sodiated mass peak at *m*/*z* 483.1987 (calcd. 483.1989). Compound **1** has UV absorption at 221 and 297 nm, corresponding to the cross-conjugated system of marine prostanoids [9]. The IR absorption band was found at 1750 cm^−1^, indicating the presence of ester functional groups. The ^1^H NMR spectrum (Table 1) of **1** revealed resonant signals corresponding to a terminal methyl group [δ_H_ 0.88 (H-20)], two acetyl methyl groups (δ_H_ 2.09 and 2.04), a methoxy group (δ_H_ 3.70), an oxymethine [δ_H_ 5.75 (H-4)], and seven olefinic methines [δ_H_ 5.84 (H-5), 6.35 (H-6), 7.39 (H-7), 6.55 (H-10), 7.48 (H-11), 6.77 (H-13), and 6.31 (H-14)]. The ^13^C NMR and DEPT NMR spectra (Table 1) of **1** demonstrated the presence of 25 carbons, which can be categorized into three methyls [δ_C_ 13.9 (C-20), 21.2 (C-2″), and 21.3 (C-2′)], one carbomethoxy [δ_C_ 51.9 (1-OMe)], one quaternary carbon [δ_C_ 83.5 (C-12)], six methylenes [δ_C_ 22.4 (C-19), 23.5 (C-17), 29.7 (C-2 and C-3, overlapped), 31.3 (C-18), and 41.2 (C-16)], eight methines [δ_C_ 69.3 (C-4), 124.4 (C-6), 126.6 (C-7), 129.2 (C-14), 136.4 (C-10), 140.1 (C-5), 141.0 (C-13), and 155.0 (C-11)], one non-protonated carbon [δ_C_ 135.7 (C-8)], three ester carbonyls [168.8 (C-1″), δ_C_ 170.0 (C-1′) and δ_C_ 172.9 (C-1)], and two carbonyls [δ_C_ 192.7 (C-9) and 199.6 (C-15)]. The COSY correlation between H-10 and H-11, along with the HMBC correlations from H-10 to C-9/C-12 and from H-11 to C-8/C-12 could establish the cyclopentenone moiety of **1** (Figure 2). Furthermore, the COSY correlations of H_2_-2 (δ_H_ 2.38 and 1.29)/H_2_-3 (δ_H_ 2.05 and 1.95)/H-4 /H-5/H-6/H-7 and the HMBC correlations form the methoxy/H-2 to C-1, H-4/H_3_-2′ to C-1′, and from H-6 to C-8, indicated the α-side chain was located at C-8 with an acetoxy group attaching at C-4. The COSY correlations of H-13/H-14, H_2_-16 (δ_H_ 2.38)/H_2_-17 (δ_H_ 1.59)/H_2_-18 (δ_H_ 1.29), H_2_-19 (δ_H_ 1.29)/H_3_-20 and the HMBC correlation from H-19 to C-18, from H-14/H-16 to C-15, and H-13 to C-12 were used to establish the connection of the ω-side chain and a ketone group located at C-15. An additional acetoxy group (2″-OAc) was assigned on C-12 due to its downfield-shifted chemical shift and considering the molecular formula. It is noted that the C=C double bond of the ω-side chain was located at C-13/C-14 in **1** instead of C-14/C-15 in clavulone I, which was rare in marine prostanoids.

**Figure 3:**
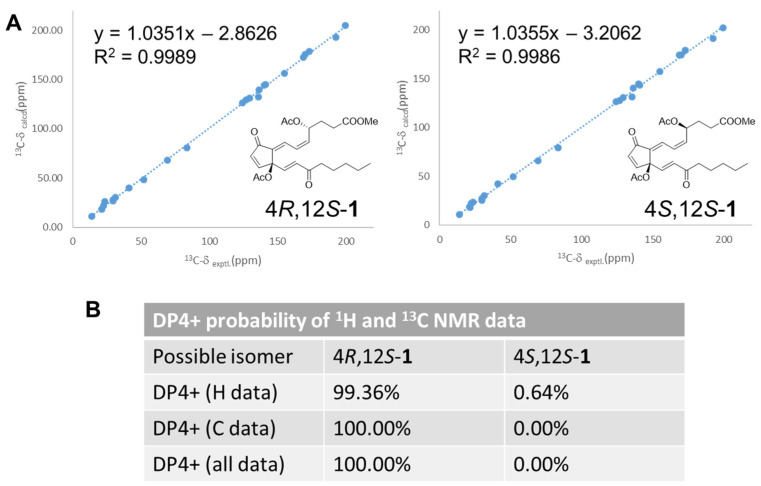
(**A**) Linear correlations between experimental and calculated ^13^C NMR data of two possible isomers. (**B**) Results of the DP4+ probability analysis.

The 5*Z* and 13*E* geometries of two disubstituted double bonds were identified by their coupling constants (*J*_5–6_ = 10.9 Hz and *J*_13–14_ =16.0 Hz). The 7*E* geometry was revealed by the de-shielding effect of H-7 caused by the anisotropy of the C-9 carbonyl group. Moreover, the presence of NOESY correlations of H-7/H-4 and H-5/H-6, and the absence of a NOESY correlation between H-13 and H-14 confirmed the above double-bond geometries (Figure 2). So far, all prostanoids isolated from *Clavularia* with an acetoxy substitution on C-12 were identified as being the *S* configuration. Thus, the configuration of C-12 in compound **1** was determined to be *S* by the biogenetic consideration. To verify the absolute configuration of C-4, the computer-generated ECD data (Figure S1) of two isomers (4*R*,12*S*-**1** and 4*S*,12*S*-**1**) were used for comparison with the experimental data. Unfortunately, none of the calculated ECD curves matched the experimental trend. Thus, the DP4+ probability analysis was selected to determine the configuration of C-4. The conformational search of two isomers was executed by Spartan 20, and the lowest energies of each conformer were calculated using Gaussian 16. The ^1^H and ^13^C NMR data of major conformers were then averaged according to the Boltzmann distribution. As a result, the isomer 4*R*,12*S*-**1** had a better correlation coefficient value (R^2^ = 0.9989) than that of 4*S*,12*S*-**1** (R^2^ = 0.9986) in comparing the ^13^C NMR chemical shifts with experimental data (Figure 3) (Tables S1–S6). Furthermore, the isomer 4*R*,12*S*-**1** also had a higher probability (all data: 100.00%) in the DP4+ analysis (Table S7). Therefore, the configuration of **1** was determined accordingly.

**Figure 4:**
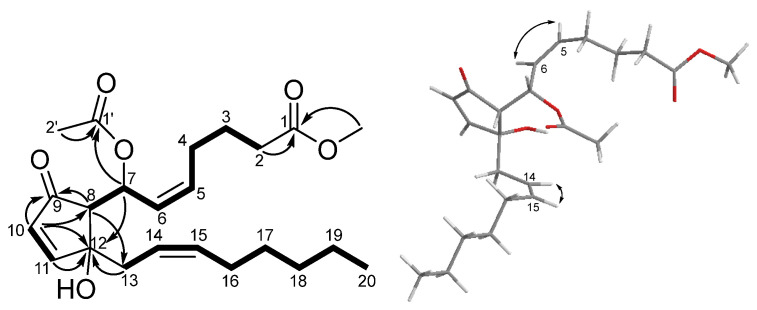
Key COSY (bold), HMBC (arrow), and NOESY (double arrow) correlations of **2**.

**Figure 5:**
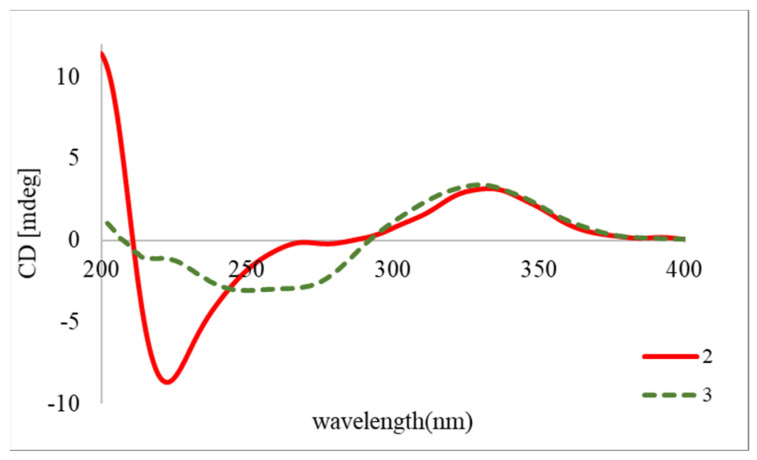
The experimental ECD spectra of compounds **2** and **3**.

12-*O*-deacetylclavulone I (**2**) was isolated as a colorless oil. Its molecular formula was determined as C_23_H_34_O_6_ (Δ = 9) by the interpretation of the mass spectrum (*m*/*z* 429.2246 [M + Na]^+^, calcd. for 429.2248). The IR spectrum indicated the presence of hydroxy (3435 cm^−1^) and ester (1717 cm^−1^) functionalities. The ^1^H NMR spectrum (Table 1) showed six olefenic methines [δ_H_ 5.57 (H-5), 5.85 (H-6), 5.94 (H-7), 6.17 (H-10), 7.43 (H-11), 5.36 (H-14), and 5.65 (H-15)], a carbomethoxy [δ_H_ 3.68 (1-OMe)], an acetyl methyl [δ_H_ 1.99 (H-1′)], and a methyl [δ_H_ 0.89 (H-20)] groups. Twenty-three carbon signals, including three methyls [δ_C_ 14.0 (C-20), 21.2 (C-2), and 51.6 (1-OMe)], eight methylenes [δ_C_ 33.4 (C-2), 24.5 (C-3), 27.0 (C-4), 39.3 (C-13), 27.4 (C-16), 29.1 (C-17), 31.5 (C-18), and 22.5 (C-19)], eight methines [δ_C_ 133.3 (C-5), 129.6 (C-6), 68.3 (C-7), 57.2 (C-8), 133.7 (C-10), 165.4 (C-11), 121.8 (C-14), and 135.7 (C-15)], two ester carbonyls [δ_C_ 174.0 (C-1) and 170.3 (C-1′)], one carbonyl [δ_C_ 204.8 (C-9)], and one non-protonated carbon [δ_C_ 79.5 (C-12)] were recognized in the ^13^C and DEPT spectra. The ^1^H and ^13^C NMR data were quite similar to those of 7-acetoxy-7,8-dihydroiodovulone I (**3**) [7], implying that **2** is a congener of **3**. The significant difference between them was the pattern of H-11 (doublet in **2** and singlet in **3**), and an additional proton (H-10) was found in **2**. The above findings suggested that the iodine atom in **3** was replaced by a hydrogen atom in **2**. The COSY spectrum (Figure 4) demonstrated the proton sequences of H_2_-2 (δ_H_ 2.34)/H_2_-3 (δ_H_ 1.72)/H_2_-4 (δ_H_ 2.22)/H-5/H-6/H-7/H-8 (δ_H_ 2.55) and H_2_-13 (δ_H_ 2.56 and 2.34)/H-14/H-15/H_2_-16 (δ_H_ 2.00)/H_2_-17 (δ_H_ 1.35)/H_2_-18 (δ_H_ 1.27)/H_2_-19 (δ_H_ 1.30)/H_3_-20. The former sequence and the HMBC correlations (Figure 4) from H-2 and 1-OMe to C-1 established the α-side chain, while the latter proton sequence constructed the ω-side chain of compound **2**. The COSY cross-peaks of H-10/H-11, in combination with the HMBC correlations of H-8/C-9, H-10/C-8, C-9, C-12, H-11/C-12, and H-13/C-12, indicated the presence of a cyclopentenone moiety and its connection to α- and ω-side chains. Finally, the acetoxy group attaching at C-4 and the hydroxy group attaching at C-12 were proved by the HMBC correlation from H-4/H-2′ to C-1′ and the chemical shift of C-12, respectively. The NOESY cross-peaks (Figure 4) of H-5/H-6 and H-14/H-15 supported the 5Z and 14Z geometries. The similar ECD trends (Figure 5) manifested between compounds **2** and **3** shared the same absolute configuration at those chiral centers. To reinforce the accuracy of structure determination, the ^1^H and ^13^C NMR data for 7*S*,8*R*,12*R*-**2** and 7*S*,8*R*,12*S*-**2** were calculated by quantum chemical calculations (Tables S8–S13). The DP4+ probability analysis showed isomer 7*S*,8*R*,12*R*-**2** had a higher probability (Table S14). Accordingly, the structure of **2** was then determined.

The known compounds were identified as 7-acetoxy-7,8-dihydroiodovulone I (**3**) [7], claviridenone E (**4**) [9], claviridenone C (**5**) [1], 20-acetoxy-claviridenone C (**6**) [1], claviridenone B (**7**) [1], indovulone III (**8**) [8], 20-acetoxy-claviridenone B (**9**) [1], 4-deacetoxyl-12-*O*-deacetylclavulone III (**10**) [8], claviridenone F (**11**) [9], claviridenone D (**12**) [1], and claviridenone A (**13**) [1] by comparing their spectrometric data with reference data.

### 2.2. Cytotoxic Evaluation of Isolated Compounds

Compounds **1**, **3**–**9**, and **11**–**13** were screened for in vitro cytotoxicity against the oral cancer cell line Ca9-22. All tested compounds demonstrated strong cytotoxicity toward the Ca9-22 cell line with IC_50_ values less than 13 μM (Table 2). Among them, the major compound **3** displayed a significant cytotoxic effect with an IC_50_ value of 3.96 µM.

### 2.3. The Predicted Possible Effects of Thirteen Compounds on Human Tumor Cell Lines for Cytotoxicity

We used the in silico tool CLC-Pred 2.0 to predict the possible cytotoxicity of test compounds for human tumor cell lines. Based on the chemical structure information of the test compound, the tool predicted three kinds of data [13]: the probability for being active (Pa), the probability for being inactive (Pi), and the invariant accuracy of prediction (IAP). Pa means the predicted chance that the test compound could belong to the sub-group of active compounds (the most typical structures of molecules in a sub-set of “actives” in the Prediction of Activity Spectra for Substances (PASS) training set). Pi means the predicted chance that the test compound could belong to the sub-group of inactive compounds (the most typical structures of molecules in a sub-set of “inactive” in the PASS training set). Only predictions with Pa > Pi could be considered possible for the particular test compound. According to the previous studies [13,14], we set the threshold as Pa > 0.7. Most of the compounds were predicted to show cytotoxicity for two human tumor cell lines, A2780cisR and PC-3, but only the in silico predictions of two compounds (**2** and **8**) were markedly different (Table S15). Compound **2** was predicted to only show cytotoxicity for A2780cisR. Compound **8** was predicted to show cytotoxicity for PC-3, HT-29, and A2780cisR. Additionally, IAP means the average accuracy of prediction obtained for the whole training set through PASS technology in the leave-one-out cross-validation method. The IAP values of compounds were located in a similar range (0.838–0.888).

### 2.4. The Predicted Possible Effects of Thirteen Compounds on NO Production in Macrophages

With the in silico tool InflamNat, we predicted the probability of the test compound with IC_50_ (inhibition for NO production) <50 μM in macrophages (Table S16). We selected aminoguanidine [15] and apigenin [16] as the positive controls, predicting the probabilities as 0.657 and 0.621 by the tool InflamNat, respectively. Most of the predicted probabilities of compounds were located in a similar range (0.310–0.383), but only one predicted probability of compound **6** was 0.430.

### 2.5. The Predicted Pharmacological Potentials of Nine Compounds

Four compounds (**6**, **9**, **12**, and **13**) were not suitable for the in silico tool SwissADME, and this kind of situation might be related to the stereochemistry of these compounds. The prediction results using the three models showed that all nine compounds might belong to the moderately soluble compounds or the water-soluble compounds (Table S17). For the pharmacokinetics of the compounds, we performed in silico predictions of gastrointestinal absorption, blood–brain barrier permeation, P-glycoprotein substrate, inhibitors for cytochrome P450, and skin permeation (Table S18). We also used the tool to predict the oral bioavailability of nine compounds (Figure S2). For the drug-likeness of the compounds, we evaluated six indicators, including Lipinski, Ghose, Veber, Egan, Muegge, and the bioavailability score (Table S19). For the medicinal chemistry of the compounds, we evaluated four indicators, including Pan Assay Interference Structures (PAINS), Brenk, lead likeness, and the synthetic accessibility score (Table S20).

## 3. Discussion

### 3.1. The Structure and Activity Relationship of Isolated Prostanoids

Marine prostanoids are found in different genera of soft corals, such as *C. viridis*, *Plexaura homomalla* [17], and *Telesto riisei* [18]. Those primitive nonmammalian prostanoids mainly possess antitumor activities. In our cytotoxic assay, although the C=C geometry at C-5 to C-8 and the oxidation/esterification at C-4, C-12, C-15, and C-20 might change the polarity of prostanoids, the cytotoxicity of isolates against oral cancer lines Ca9-22 remains. This finding implied that the skeleton of the prostanoids might demonstrate cytotoxic effects. Our discovery reinforces the cytotoxic potential of coral extracts and underscores the genus *Clavularia* as a promising source of bioactive marine natural products with anticancer properties.

### 3.2. The Predicted Possible Cytotoxicity of Thirteen Compounds for Human Tumor Cell Lines

Most of the compounds were predicted to show cytotoxicity for two tumor cell lines (A2780cisR and PC-3) (Table 1), but compound **2** was predicted to only show cytotoxicity for A2780cisR, and compound **8** was predicted to show cytotoxicity for PC-3, HT-29, and A2780cisR. A2780cisR is the cisplatin-resistant human ovarian carcinoma [19], PC-3 is the human prostate carcinoma [20], and HT-29 is the human colon adenocarcinoma [21]. In addition, the in silico tool CLC-Pred 2.0 provides the prediction of possible cytotoxicity of the test compound against 391 human tumor cell lines [13], but the cell lines do not include the oral cancer cell line. The important role of inflammation is in regard to inducible nitric oxide synthase (iNOS), which yields the pro-inflammatory mediator nitric oxide (NO) from *L*-arginine [22], and the overexpressed iNOS could contribute to numerous diseases, including cancers [23]. NO has been reported to participate in tumor progression and cancer metastasis, and some studies showed the potential of NOS inhibitors for anticancer therapies [24], such as the studies about triple-negative breast cancer [25] and colorectal cancer [26]. In macrophages, a NOS inhibitor (aminoguanidine [15]) and a flavonoid derivative (apigenin [16]) were reported to have IC_50_ values of 15.06 and 23 μM for inhibition of NO production, respectively. With the in silico tool InflamNat, the predicted probabilities of the positive controls (aminoguanidine and apigenin) with IC_50_ (inhibition for NO production) <50 μM in macrophages were in a similar range (0.621–0.657), but most of the predicted probabilities of compounds were below 0.385 (Table S16). Because only one predicted probability of compound **6** was 0.430, we suggested that future investigators might prioritize examining the possible effect of compound **6** on NO production.

### 3.3. The Pharmacological Potentials of Nine Compounds

By reviewing the previous studies about the clinical trials (from 2010 to 2017), Sun et al. summarized the four possible reasons for about 90% of failures of clinical drug development: the poor clinical therapeutic effects (40–50%), the unexpected or unmanageable toxicity (30%), the poor drug-like properties (10–15%), and the low commercial needs or the weak strategic project (10%) [27]. For evaluation of the possible pharmacological potentials of the compounds, we performed the in silico predictions of the properties of compounds, including water solubility (Table S17), pharmacokinetics (Table S18), oral bioavailability (Figure S2), drug-likeness (Table S19), and medicinal chemistry property (Table S20). The oral administration of poorly water-soluble molecules usually has two disadvantages [28]: (a) they need high doses for the therapeutic plasma concentrations; and (b) the molecules with slow absorption could lead to inadequate, variable bioavailability, or gastrointestinal mucosal toxicity. In the clinical drug development pipeline, the difficulties encountered by about 40–70% of molecules includes the poor aqueous solubility, absorption, and bioavailability [29]. In this study, all nine compounds might belong to the moderately soluble compounds or the water-soluble compounds (Table S17). The gastrointestinal absorption of all nine compounds was predicted to be high (Table S18). The blood–brain barrier (BBB) restricts the access of certain compounds with therapeutic effects on the central nervous system from the peripheral system, and makes them useless [30]. Only four compounds (**4**, **8**, **10**, and **11**) might show blood–brain barrier permeation. Additionally, most of the predicted Log *K*_p_ values for skin permeation of compounds were located in a similar range (−6.55 to −5.26), but only one predicted Log *K*_p_ value of compound **1** was −7.04. For the predicted oral bioavailability, the colored zone in Figure S2 could be the suitable physicochemical space of the ideal compound, and six red spots showed the predicted values for the six properties of the test compound. Almost all of the five values of nine compounds were located in the ideal zone, but only one value (size) of compound **3** was not located in the ideal zone. And, only one value (flexibility) of nine compounds was not located in the ideal zone. There are two key roles for pharmacokinetics: P-glycoprotein and cytochrome P450 (CYP) enzymes. P-glycoprotein, the drug transporter, could remove some substances from cells (especially chemotherapeutic drugs) and participate in the development of the resistance of cancer cells to chemotherapeutic drugs [31]. All nine compounds might be P-glycoprotein substrates (Table S16). CYP enzymes participate in mediating the metabolism for over 75% of marketed drugs [32], and five (1A2, 2C9, 2C19, 2D6, and 3A4) of the CYPs could contribute to about 95% of the metabolism of drugs via CYPs in humans [33]. Most of the compounds were predicted to be inhibitors of CYP (Table S18). For the evaluation of four indicators of drug-likeness (Lipinski, Ghose, Egan, and Muegge), only two compounds (**1** and **3**) failed to pass the filters of one or two indicators (Table S19). All nine compounds did not pass the Veber filter. All nine compounds had the same bioavailability score (0.55). For the evaluation of one indicator of medicinal chemistry (Pan Assay Interference Structures (PAINS)), only two compounds (**3** and **8**) caused alerts (Table S20). All nine compounds caused alerts for Brenk, and did not pass the filter of lead-likeness. Nevertheless, some scientists provided a reminder that the multifarious definitions of both drug-likeness and lead-likeness might severely reduce the possibility of molecular diversity for drug discovery [34]. The clinical development of a natural compound requires a sustainable source compound to be available at a reasonable cost [35], especially marine natural compounds [36]. The synthetic accessibility scores of all nine compounds were located in a similar range (4.58 to 5.41; Table S20), which might be at the moderately difficult level for the synthetic accessibility of the compounds.

## 4. Materials and Methods

### 4.1. General

The Varian (Palo Alto, CA, USA) Mercury Plus 400 MHz and VNMRS 600 MHz FT-NMR spectrometers provided the NMR spectra. HRSIMS was performed by using a Bruker APEX II spectrometer (Bremen, Germany). The V-650 spectrophotometer from Jasco (Tokyo, Japan) was utilized to measure the UV data. For measuring infrared data, the Jasco FT/IR-4X spectrophotometer was selected. Circular dichroism data were recorded using a Jasco J-815 CD spectrometer. A Jasco P-2000 polarimeter was used to quantify specific optical rotation. Merck KGaA (Darmstadt, Germany) silica gel 60 (0.015–0.040 mm) was utilized to pack columns. For high-performance liquid chromatography (HPLC), Phenomenex (Torrance, CA, USA) C18, phenyl-hexyl, and biphenyl columns were utilized. The LC-40D solvent delivery module, DGU-405 de-gassing unit, CBM-40 system controller, CTO-40S column oven, SPD-M40 photo diode array detector, and FRC-10A fraction collector made up the Shimadzu (Kyoto, Japan) HPLC apparatus.

### 4.2. Animal Material

The specimens of *Clavularia* spp. collected in Green Island, Taiwan, were identified by the corresponding author. The collection was stored at the Department of Marine Biotechnology and Resources, National Sun Yat-sen University, and a voucher ID (CI2021) was given.

### 4.3. Extraction and Isolation

The coral material was frozen to remove water and then extracted by ethanol three times. The ethanol extract was divided into an ethyl acetate portion and a water portion, and the ethyl acetate soluble portion was partitioned between hexanes and 75% methanol. The 75% methanol portion was fractionated using a silica gel flash column. Nine fractions (A–I) were collected by eluting with hexanes/EtOAc/MeOH (8/1/0 to 0/0/1). Fraction C (494.7 mg) was fractionated by silica gel CC eluted with hexanes/methylene chloride/acetone (8/2/0 to 0/0/1) to afford subfractions (C1–C7). Subfraction C5 (95.0 mg) was separated by a silica gel CC, eluted stepwise by hexanes/acetone (15/1 to 0/1) to give fractions C5A–C5E. The reverse-phase HPLC (phenyl hexyl column) separation of fraction C5C (24.0 mg) eluting with 75% methanol produced compound **11** (5.4 mg). Subfraction C5D (13.5 mg) was also separated by reverse-phase HPLC (phenyl hexyl column) isocratically eluted by 75% methanol to yield compound **7** (1.0 mg). Subfraction C6 (136.1 mg) was subjected to silica gel CC eluted stepwise with hexanes/acetone/methanol (6/1/0 to 0/0/1), and three fractions (C6A–C6C) were obtained. Fraction C6B (95.0 mg) was partitioned by C_18_ CC eluted stepwise with methanol/water (7/3 to 1/0) to afford six subfractions, C6B1–C6B6. Subfraction C6B3 (25.4 mg) was purified by reverse-phase HPLC (C_18_ column) with 75% methanol to yield compound **5** (6.1 mg). Compounds **3** (4.7 mg) and **8** (0.9 mg) were isolated from subfraction C6B4 (18.2 mg) by reverse-phase HPLC (C_18_ column) eluted with 60% methanol. Fraction D (2.7 g) eluted with hexanes/ethyl acetate/methanol (100/10/1 to 0/0/1) was further separated by silica gel CC to obtain six subfractions (D1–D6). Subsequent separation of subfraction D2 (152.1 mg) by silica gel CC, eluted stepwise with methylene chloride/methanol/acetone (120/1/0 to 0/0/1), gave three fractions (D2A–C6C). Fraction D2B (100.8 mg) was further isolated by silica gel CC eluted stepwise with methylene hexanes/acetone/methanol (20/10/1 to 0/0/1) to afford subfraction D2B5 (39.9 mg). This subfraction was purified by semi-preparative reverse-phase HPLC (C_18_ column) gradient eluted with acetonitrile/water (0–10 min, 50% acetonitrile; 10–15 min, 50–60% acetonitrile; 15–50 min, 60% acetonitrile) to give compounds **4** (0.9 mg) and **13** (1.6 mg). Subfraction D4 (986.2 mg) was separated by silica gel CC methylene chloride/acetone/methanol (120/1/0 to 0/0/1) to yield six subfractions D4A–D4F. Subfraction D4D (294.2 mg) was subjected to silica gel CC using hexanes/methylene chloride/methanol (70/20/1 to 0/0/1) as the eluent, and a prostanoid-enriched fraction D4D2 was obtained. Fraction D4D2 (184.4 mg) was isolated by reverse-phase HPLC (C_18_ column) gradient eluted with acetonitrile/water (0–35 min, 55% acetonitrile; 35–36 min, 55–70% acetonitrile; 36–55 min, 70% acetonitrile) to obtain compounds **1** (1.8 mg) and **12** (40.1 mg). Fraction D4D4 (65.6 mg) was purified by normal-phase HPLC (silica column) eluted with *n*-hexane/methylene chloride/methanol (70/10/1) to yield compound **10** (2.2 mg). Fraction D4E (65.3 mg) was separated by semi-preparative normal-phase HPLC (silica column) eluted with *n*-hexane/methylene chloride/methanol (80/10/1) to give compounds **6** (1.4 mg) and **9** (1.0 mg) and subfraction D4E4 (15.1 mg). This subfraction was further purified by reverse-phase HPLC (phenyl-hexyl column) eluted with 50% acetonitrile, and compound **2** (0.6 mg) was obtained.

### 4.4. Spectroscopic Data

Clavulone I-15-one (**1**): colorless oil; [α]^D^_25_ −57 (c 0.05, MeOH); UV λ_max_ (log *ε*) 221 (3.58), 297 (3.43) nm; IR (neat) *v*_max_ 3076, 2952, 2883, 1750, 1551, 1464, 1383, 1241, 1031 cm^−1^; ECD (*c* 0.0001, MeOH) λ_max_(Δε) 339 (+0.32), 290 (−1.92), 264 (−2.09), 231 (−0.42), 208 (+1.10) nm; ^1^H and ^13^C NMR data are presented in Table 1; HRESIMS *m*/*z* 483.1987 [M + Na]^+^ (calcd for C_25_H_32_NaO_8_, 483.1989).

12-*O*-Deacetylclavulone I (**2**): colorless oil; [α]^D^_25_ −98 (c 0.035, MeOH); UV λ_max_ (log *ε*) 210 (3.21), 297 (2.56) nm; IR (neat) *v*_max_ 3435, 2925, 2859, 1717, 1445, 1372, 1238, 1136, 1024 cm^−1^; ECD (*c* 0.0007, MeOH) λ_max_(Δε) 333 (+1.17), 223 (−3.21) nm; ^1^H and ^13^C NMR data are presented in Table 2; HRESIMS *m*/*z* 429.2246 [M + Na]^+^ (calcd for C_23_H_34_NaO_6_, 429.2248).

### 4.5. Parameter of NMR and ECD Calculations

The NMR and ECD calculations were performed using Gaussian 16 software. Initially, potential conformers were generated using Spartan20 software. Conformers exhibiting a probability greater than 1% were optimized using Gaussian 16 at the B3LYP/6-31G(d) level. The electronic and thermal free energies of each conformer were summed, and the Boltzmann distribution ratio was computed. Theoretical calculations of NMR data were carried out using the Gauge-Including Atomic Orbitals (GIAO) method at mPW1PW91/6-311G(d,p) in chloroform. Subsequently, regression analyses were conducted to compare calculated and experimental NMR chemical shifts, with linear R^2^ values calculated to assess the results. ECD spectra were computed using the parameters of B3LYP/6-311G++(d,p).

### 4.6. Cytotoxicity Assays

A luminometer (Berthold Technologies GmbH and Co., Bad Wildbad, Germany) and an ATP detection kit (PerkinElmer Life Sciences, Boston, MA, USA) [37,38] were used to quantify the cell viability (IC50) of oral cancer Ca9-22 cells (HSRRB, Ibaraki, Osaka, Japan) at 72 h [39]. These data are presented as means ± SD in three independent experiments.

### 4.7. In Silico Prediction and Evaluation of Isolated Compounds

With the in silico tool CLC-Pred 2.0 [13] (Department for Bioinformatics, Laboratory for Structure–Function Based Drug Design, Institute of Biomedical Chemistry of the Russian Academy of Medical Sciences, Moscow, Russia), we predicted the possible cytotoxicity of test compounds for human tumor cell lines. By the in silico tool InflamNat [40] (Yunnan University, Kunming, China), we predicted the possible effects of test compounds on NO production in macrophages. The results could be shown as the probability of the test compound with IC_50_ (inhibition for NO production) <50 μM in macrophages without cytotoxicity. In addition, the tool InflamNat was mainly designed for natural products, so it may not be effectively applied to synthetic compounds. Using the in silico tool SwissADME [41] (Swiss Institute of Bioinformatics, Lausanne, Switzerland), we predicted the pharmacological potentials of test compounds, including (1) the water solubility (by three kinds of models), pharmacokinetics, (2) drug-likeness, (3) medicinal chemistry property, and (4) oral bioavailability.

## 5. Conclusions

The present investigation on octocoral *Clavularia* spp. resulted in the isolation and characterization of thirteen marine prostanoids, including two new ones. The absolute configuration of new components was determined by the comparison of ECD data and DP4+ probability analysis. In our continuously chromatographic study of *Clavularia* spp., three types of marine natural products (prostanoids, eudensamanes, and dollabellanes) were identified. Among those isolated compounds, prostanoids show the most potent cytotoxic activity against oral cancer cell line Ca9-22. Our findings lay a foundation for further developing or utilizing marine octocorals and prostanoids as anti-oral cancer agents.

## Figures and Tables

**Table 1:** ^1^H-NMR and ^13^C-NMR data for compounds **1** and **2**.

	1 ^a^		2 ^a^	
	δ_H_ (*J* in Hz)	δc, Type	δ_H_ (*J* in Hz)	δc, Type
1		172.9, C		174.0, C
2	2.38, m	29.7, CH_2_	2.34, t (7.5)	33.4, CH_2_
	1.29, m			
3	2.05, m	29.7, CH_2_	1.72, m	24.5, CH_2_
	1.95, m			
4	5.75, m	69.3, CH	2.22, m	27.0, CH_2_
5	5.84, dd (10.9, 8.6)	140.0, CH	5.57, dt (10.6, 7.5)	133.3, CH
6	6.35, m	124.4, CH	5.85, dd (10.6, 9.5)	129.6, CH
7	7.39, d (12.5)	126.6, CH	5.94, dd (9.5, 3.6)	68.3, CH
8		135.7, C	2.55, d (3.6)	57.2, CH
9		192.7, C		204.8, C
10	6.55, d (6.1)	136.4, CH	6.17, d (5.8)	133.7, CH
11	7.48, d (6.1)	155.0, CH	7.43, d (5.8)	165.4, CH
12		83.5, C		79.5, C
13	6.77, d (16.0)	141.1, CH	2.56, dd (13.9, 8.3)	39.3, CH_2_
			2.34, m	
14	6.31, d (16.0)	129.2, CH	5.36, dt (10.5, 6.9)	121.8, CH
15		199.6, C	5.65, dt (10.5, 7.4)	135.7, CH
16	2.53, t (7.4)	41.2, CH_2_	2.00, m	27.4, CH_2_
17	1.59, m	23.5, CH_2_	1.35, m	29.1, CH_2_
18	1.29, m ^b^	31.3, CH_2_	1.27, m	31.5, CH_2_
19	1.29, m ^b^	22.4, CH_2_	1.30, m	22.5, CH_2_
20	0.88, t (7.0)	13.9, CH_3_	0.89, t (6.8)	14.0, CH_3_
1-OMe	3.70, s	51.9, CH_3_	3.68, s	51.6, CH_3_
1′		170.0, C		170.3, C
2′	2.04, s	21.3, CH_3_	1.99, s	21.2, CH_3_
1″		168.8, C		
2″	2.09, s	21.2, CH_3_		

^a 1^H and ^13^C data were obtained in CDCl_3_ at 600 and 150 MHz, respectively; ^b^ overlapped.

**Table 2:** Cytotoxic data (IC_50_, µM) of isolated compounds.

Compound/Tumor Cell	Ca9-22
**1**	4.85 ± 0.52
**3**	3.96 ± 0.06
**4**	12.4 ± 0.41
**5**	5.13 ± 0.11
**6**	4.34 ± 0.06
**7**	5.15 ± 0.18
**8**	10.65 ± 0.64
**9**	9.57 ± 0.32
**11**	12.72 ± 0.88
**12**	4.70 ± 0.20
**13**	10.82 ± 0.36
cisplatin ^a^	1.08 ± 0.10

^a^ Positive control.

## Data Availability

The original data presented in the study are included in the article/Supplementary Material; further inquiries can be directed to the corresponding author.

## Supplementary Materials

The following supporting information can be downloaded at: https://www.mdpi.com/article/10.3390/md22050219/s1, Tables S1–S14: Quantum chemical calculations of compounds **1** and **2**; Tables S15–S20: The in silico predicted data; Figure S1: Experimental and calculated ECD spectra of 1; Figure S2: The predicted oral bioavailability of nine compounds; Figures S3–S8: 1D and 2D NMR spectra of **1**; Figure S9: HRESIMS spectrum of **1**; Figure S10: UV spectrum of **1**; Figure S11: IR spectrum of **1**; Figures S12–S17: 1D and 2D NMR spectra of **2**; Figure S18: HRESIMS spectrum of **2**; Figure S19: UV spectrum of **2**; Figure S20: IR spectrum of **2**.
