# Supplementary material for: Marine Prostanoids with Cytotoxic Activity from Octocoral Clavularia spp
Source: Mar Drugs. 2024 May 14;22(5):219. doi: 10.3390/md22050219 (PMC11122631; doi:10.3390/md22050219)
Supplement: Supplementary file 1 [file marinedrugs-22-00219-s001.zip › marinedrugs-2996810-supplementary.pdf]

Supplementary Materials

Marine Prostanoids with Cytotoxic Activity from Octocoral *Clavularia* spp.

Ming-Ya Cheng [1,†], I-Chi Hsu [2,†], Shi-Ying Huang [3,4], Ya-Ting Chuang [5], Hsueh-Wei Chang [5,6,7], Tian-Huei Chu [8], Ching-Yeu Chen [9] and Yuan-Bin Cheng [1,10,*]

Corresponding Author

Yuan-Bin Cheng − Department of Marine Biotechnology and Resources, National Sun Yat-sen University, Kaohsiung 80424, Taiwan; orcid.org/0000-0001-6581-1320; E-mail: jmb@mail.nsysu.edu.tw; Tel.: +886-7-5252-000-5212

Authors

Ming-Ya Cheng − Department of Marine Biotechnology and Resources, National Sun Yat-sen University, Kaohsiung 80424, Taiwan

I-Chi Hsu − Division of Pharmacy, Zuoying Branch of Kaohsiung Armed Forces General Hospital, Kaohsiung 813204, Taiwan

Shi-Ying Huang − College of Food and Biological Engineering, Jimei University, Xiamen 361021, China; Fujian Provincial Engineering Technology Research Center of Marine Functional Food, Xiamen 361021, China

Ya-Ting Chuang − Department of Biomedical Science and Environmental Biology, PhD program of Life Science, College of Life Science, Kaohsiung Medical University, Kaohsiung 80708, Taiwan

Hsueh-Wei Chang − Department of Biomedical Science and Environmental Biology, PhD program of Life Science, College of Life Science, Kaohsiung Medical University, Kaohsiung 80708, Taiwan; Center for Cancer Research, Kaohsiung Medical University, Kaohsiung 80708, Taiwan; Department of Medical Research, Kaohsiung Medical University Hospital, Kaohsiung 80708, Taiwan

Tian-Huei Chu − Medical Laboratory, Medical Education and Research Center, Kaohsiung Armed Forces General Hospital, Kaohsiung City, 802301

Ching-Yeu Chen − Department of Physical Therapy, Tzu-Hui Institute of Technology, Pingtung 926001, Taiwan

Content

Table S1. Energy analyses of 4*R*,12*S*-**1**/4*S*,12*R*-**1** (fourteen conformers)....4
Table S2. Cartesian coordinates of the low-energy re-optimized conformers of 4*R*,12*S*-**1**/4*S*,12*R*-**1** calculated at B3LYP/6-31G(d,p) level of theory. 7
Table S3. Energy analyses of 4*S*,12*S*-**1**/4*R*,12*R*-**1** (ten conformers)....21
Table S4. Cartesian coordinates of the low-energy re-optimized conformers of 4*S*,12*S*-**1**/4*R*,12*R*-**1** calculated at B3LYP/6-31G(d,p) level of theory. 24
Table S5. Experimental and calculated ^{1}H NMR data for compound **1**....34
Table S6. Experimental and calculated ^{13}C NMR data for compound **1**. 35
Table S7. DP4+ analyses of calculated and experimental NMR chemical shifts of **1** (unscaled). Isomer 1: 4*R*,12*S*-**1**; Isomer 2: 4*S*,12*S*-**1**....36
Table S8. Energy analyses of 7*S*,8*R*,12*R*-**2**/7*R*,8*S*,12*S*-**2** (eleven conformers)....37
Table S9. Cartesian coordinates of the low-energy re-optimized conformers of 7*S*,8*R*,12*R*-**2**/7*R*,8*S*,12*S*-**2** calculated at B3LYP/6-31G(d,p) level of theory....39
Table S10. Energy analyses of 7*S*,8*R*,12*S*-**2**/7*R*,8*S*,12*R*-**2** (five conformers)....50
Table S11. Cartesian coordinates of the low-energy re-optimized conformers of 7*S*,8*R*,12*S*-**2**/7*R*,8*S*,12*R*-**2** calculated at B3LYP/6-31G(d,p) level of theory. 51
Table S12. Experimental and calculated ^{1}H NMR data for compound **2**....56
Table S13. Experimental and calculated ^{13}C NMR data for compound **2**. 57
Table S14. DP4+ analyses of calculated and experimental NMR chemical shifts of **2** (unscaled). Isomer 1: 7*S*,8*R*,12*R*-**2**; Isomer 2: 7*S*,8*R*,12*S*-**2**....58
Table S15. *In silico* prediction of cytotoxicity of the compounds for human tumor cell lines. 60
Table S16. *In silico* prediction of the nitric oxide (NO) production inhibition activity of the compounds. 61
Table S17. The *in silico* predicted water solubility of the compounds. 62
Table S18. The *in silico* predicted pharmacokinetics of the compounds. 62
Table S19. The *in silico* evaluation of the compounds for druglikeness....63
Table S20. The *in silico* evaluation of the compounds for medicinal chemistry. 64

Figure S1. Experimental and Calculated ECD of **1** 65
Figure S2. The predicted oral bioavailability of nine compounds. 65
Figure S3. ^{1}H NMR spectrum of **1** (600 MHz, $CDCl_3$)....66

Figure S4. ^{13}C NMR spectrum of **1** (125 MHz, $CDCl_3$) 66
Figure S5. COSY spectrum of **1** 67
Figure S6. HSQC spectrum of **1** 67
Figure S7. HMBC spectrum of **1** 68
Figure S8. NOESY spectrum of **1** 68
Figure S9. HRESIMS spectrum of **1** 69
Figure S10. UV spectrum of **1** 70
Figure S11. IR spectrum of **1** 70
Figure S12. ^{1}H NMR spectrum of **2** (600 MHz, $CDCl_3$) 71
Figure S13. ^{13}C NMR spectrum of **2** (125 MHz, $CDCl_3$) 71
Figure S14. COSY spectrum of **2** 72
Figure S15. HSQC spectrum of **2** 72
Figure S16. HMBC spectrum of **2** 73
Figure S17. NOESY spectrum of **2** 73
Figure S18. HRESIMS spectrum of **2** 74
Figure S19. UV spectrum of **2** 75
Figure S20. IR spectrum of **2** 75

Table S1. Energy analyses of 4*R*,12*S*-**1**/4*S*,12*R*-**1** (fourteen conformers)

**NO.**	**3D conformers** B3LYP/6-31G(d,p)	**G (Hartree)**	**Boltzmann distribution**	**Calculated ECD spectrum** 4*R*,12*S*-**1**
1	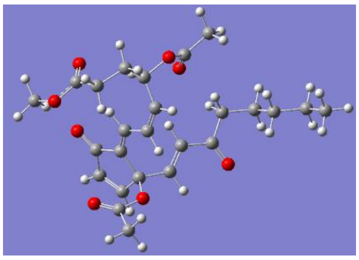	-1573.243097	18.53 %	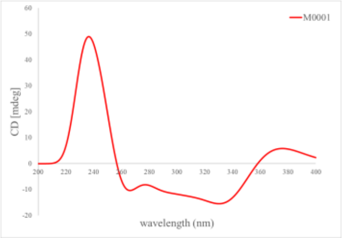
2	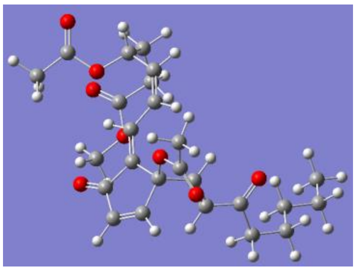	-1573.242279	7.79 %	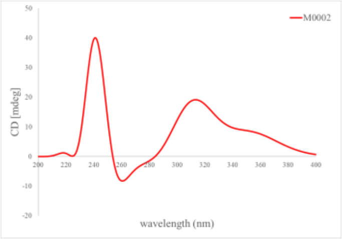
3	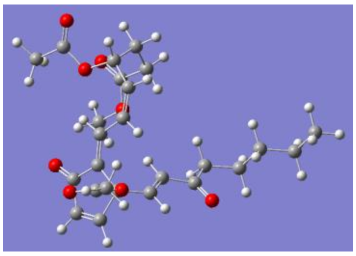	-1573.240387	1.05 %	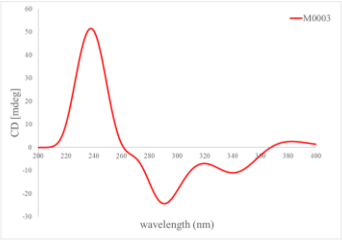
4	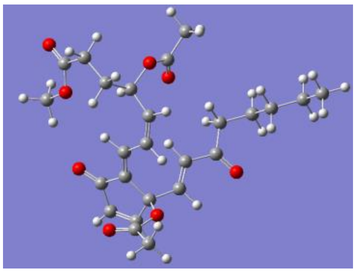	-1573.243648	33.21 %	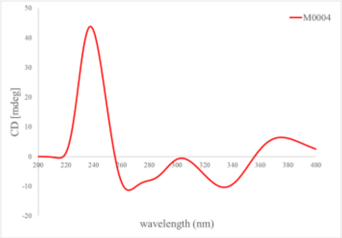

5	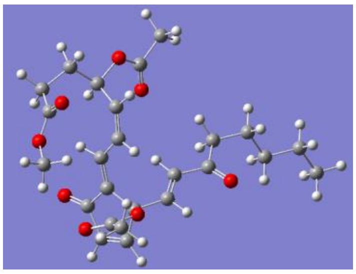	-1573.240860	1.73 %	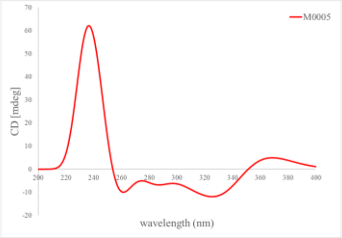
6	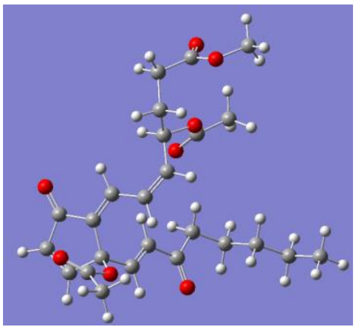	-1573.241282	2.71 %	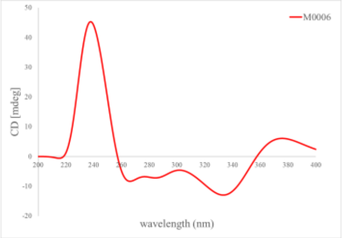
7	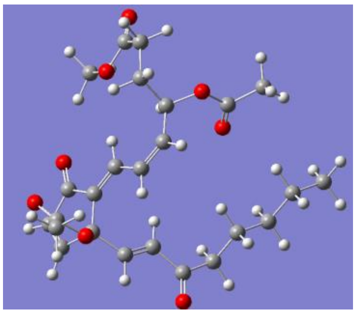	-1573.242657	11.63 %	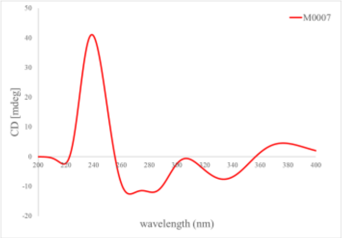
8	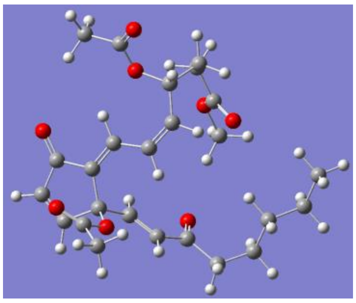	-1573.241846	4.93 %	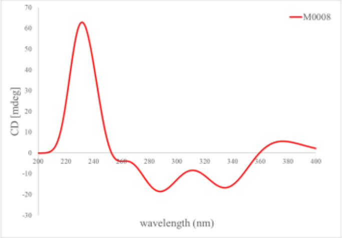
9	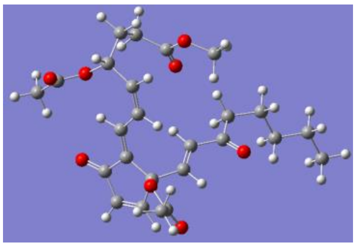	-1573.240792	1.61 %	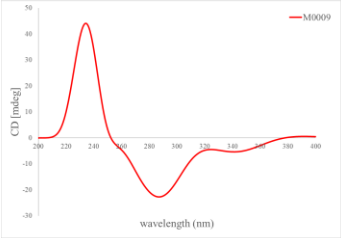

10	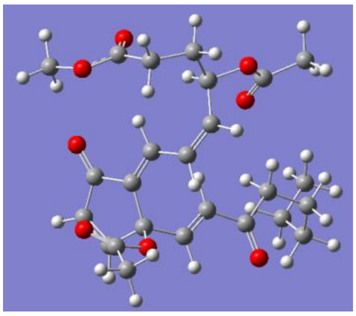	-1573.239958	0.67 %	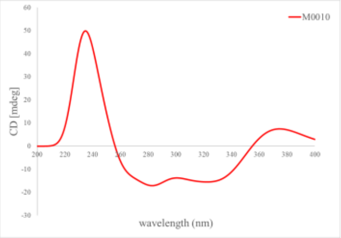
11	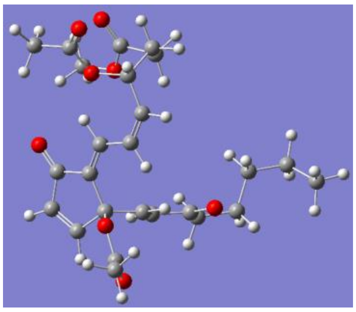	-1573.240849	1.71 %	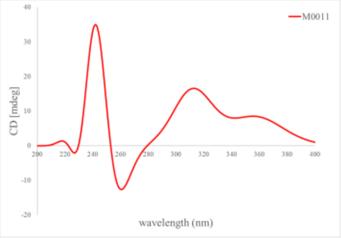
12	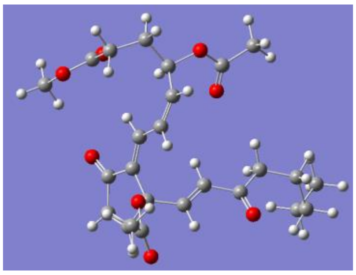	-1573.240968	1.94 %	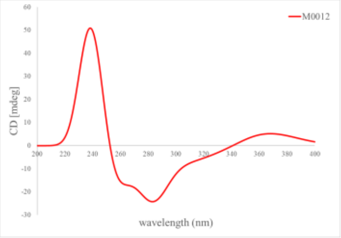
13	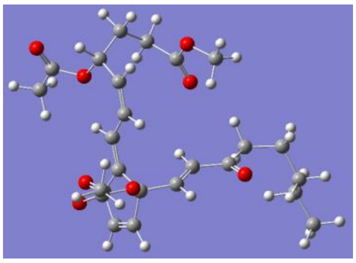	-1573.239961	0.67 %	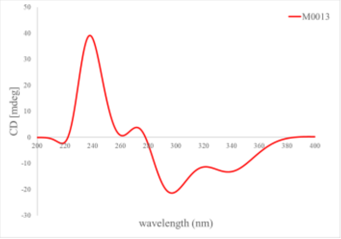
14	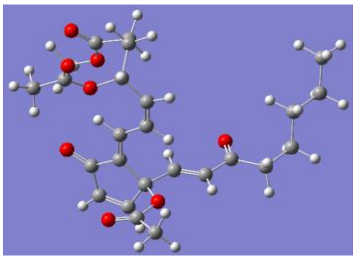	-1573.242638	11.4 %	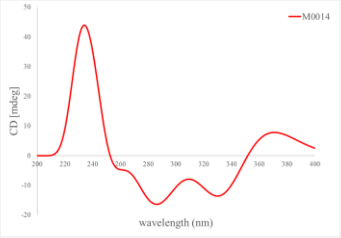

Table S2. Cartesian coordinates of the low-energy re-optimized conformers of 4*R*,12*S*-**1**/4*S*,12*R*-**1** calculated at B3LYP/6-31G(d,p) level of theory.

Conformer 1							
Atomic	Standard Orientation (Å)			Atomic	Standard Orientation (Å)		
Type	X	Y	Z	Type	X	Y	Z
C	-1.133	-3.757	-1.0164	H	-0.7578	-4.7693	-0.9036
C	-0.6894	-2.6582	-0.0532	H	-2.3736	-3.8718	-2.7963
C	-1.4742	-1.4556	-0.5425	H	1.36952	-3.3393	0.3506
C	-2.2508	-1.8671	-1.7517	H	-2.0528	0.51974	-0.6661
C	-1.9483	-3.3045	-1.9767	H	-0.4089	-0.4845	1.78584
O	-2.9916	-1.1606	-2.4181	H	1.01989	-0.7159	-1.2075
C	0.81798	-2.5282	-0.1232	H	-0.0394	1.7951	2.35781
C	-1.4702	-0.1899	-0.0822	H	-1.1386	2.50708	-0.4286
C	-0.7785	0.27955	1.10641	H	-1.7491	3.96149	2.19498
C	1.51177	-1.5581	-0.7293	H	-2.1344	4.52437	0.56486
C	-0.5547	1.56947	1.42423	H	-3.3171	1.99533	1.87642
C	-0.9025	2.77838	0.59942	H	-4.125	3.54291	1.75532
C	-2.0359	3.63076	1.18953	H	3.40045	-0.5878	-2.5698
C	-3.3817	2.90174	1.26441	H	3.19127	0.49955	-1.2262
C	3.008	-1.5825	-0.7479	H	5.61518	-1.3766	-1.5422
C	3.67375	-0.4469	-1.5112	H	5.59935	0.28627	-2.1274
O	3.64369	-2.471	-0.1982	H	5.22337	1.1037	0.22041
C	5.19537	-0.381	-1.3541	H	5.25196	-0.5612	0.7975
C	5.65328	0.10873	0.02668	H	7.6059	-0.8117	-0.0271
C	7.17899	0.18267	0.16496	H	7.58418	0.84375	-0.6149
C	7.63705	0.6765	1.54107	H	8.73031	0.71533	1.61038
O	-0.9218	-3.0267	1.34698	H	7.25463	1.68384	1.7489
O	-3.1768	-3.1888	1.08333	H	7.27576	0.01549	2.33845
C	-2.1923	-3.2679	1.7781	H	-3.192	-3.8705	3.5667
C	-2.1767	-3.6377	3.24401	H	-1.5219	-4.4985	3.41245
C	-3.9319	2.5175	-0.1002	H	-1.7814	-2.8062	3.83709
O	-3.4836	2.89885	-1.1633	H	-6.3949	0.59721	-0.9394
O	-4.9806	1.68674	0.01689	H	-5.8282	2.02745	-1.8668
C	-5.5292	1.19442	-1.2261	H	-4.7895	0.57789	-1.7431
O	0.25841	3.66397	0.56924	H	3.20139	4.03294	-0.8441
O	1.28823	2.30794	-0.9293	H	2.01627	5.3165	-0.4592
C	1.2809	3.30862	-0.2441	H	2.76921	4.40648	0.85129
C	2.39342	4.32898	-0.1743				

Conformer 2							
Atomic	Standard Orientation (Å)			Atomic	Standard Orientation (Å)		
Type	X	Y	Z	Type	X	Y	Z
C	-0.8989	-3.2806	1.6654	H	-1.7084	-3.9976	1.71769
C	-0.6039	-2.555	0.35793	H	-0.1062	-3.2457	3.68715
C	0.61684	-1.7123	0.71626	H	-1.783	-1.5109	-1.2043
C	0.88808	-1.8883	2.17177	H	2.22836	-0.4522	0.46223
C	-0.0893	-2.8964	2.66087	H	0.45818	-1.2129	-1.9796
O	1.74829	-1.3228	2.83231	H	-2.7279	-1.282	1.7025
C	-1.7556	-1.7079	-0.134	H	1.754	0.19024	-3.2949
C	1.40898	-0.9384	-0.0547	H	3.99409	0.79208	-2.7031
C	1.27187	-0.6966	-1.4769	H	3.85653	3.0069	-1.7719
C	-2.6933	-1.1302	0.62598	H	2.53062	2.73927	-2.9033
C	2.04025	0.10418	-2.2466	H	1.63996	3.95559	-1.0249
C	3.2381	0.95461	-1.9265	H	0.97601	2.32746	-0.9061
C	2.91519	2.4672	-1.9128	H	-5.3411	-0.5864	1.35073
C	1.90229	2.90455	-0.8452	H	-4.2903	0.72369	1.83131
C	-3.7344	-0.2569	0.00897	H	-6.1484	0.86312	-0.613
C	-4.7967	0.28676	0.95704	H	-6.6292	1.41156	0.99192
O	-3.7428	-0.0146	-1.1896	H	-4.7507	3.08285	0.9798
C	-5.7667	1.28788	0.32323	H	-4.2837	2.53567	-0.6293
C	-5.1428	2.66152	0.04119	H	-6.5209	3.2336	-1.5199
C	-6.1315	3.65458	-0.5823	H	-6.9994	3.77125	0.08295
C	-5.5121	5.02834	-0.8586	H	-6.2408	5.71406	-1.3061
O	-0.1874	-3.483	-0.6975	H	-5.1437	5.49084	0.0656
O	-2.1915	-4.5515	-0.6709	H	-4.6631	4.94747	-1.5485
C	-1.0774	-4.4181	-1.1251	H	-1.1892	-6.012	-2.5417
C	-0.4731	-5.2509	-2.2308	H	-0.2141	-4.6137	-3.0828
C	2.45155	2.83408	0.57085	H	0.45144	-5.7235	-1.8842
O	3.56456	3.19124	0.90095	H	2.29665	1.2176	2.96878
O	1.52128	2.35742	1.42038	H	2.63981	2.97793	3.07472
C	1.89981	2.22279	2.80549	H	0.98162	2.35353	3.38046
O	3.82365	0.56907	-0.6668	H	6.69456	0.34675	0.90493
O	5.88491	1.10394	-1.4469	H	5.31252	1.31159	1.49657
C	5.17297	0.74473	-0.5375	H	5.11722	-0.4328	1.26946
C	5.61012	0.45995	0.87448				

Conformer 3							
Atomic	Standard Orientation (Å)			Atomic	Standard Orientation (Å)		
Type	X	Y	Z	Type	X	Y	Z
C	0.41482	-3.7788	1.37924	H	-0.1363	-4.7127	1.42871
C	0.20243	-2.816	0.21177	H	1.54832	-3.7598	3.23236
C	1.2211	-1.7286	0.49583	H	-1.9572	-3.1069	-0.1449
C	1.82273	-1.9986	1.83534	H	2.32147	-0.0054	0.20152
C	1.26572	-3.2973	2.29396	H	0.35531	-1.0135	-1.9888
O	2.60294	-1.2781	2.44264	H	-1.0528	-0.4271	1.05237
C	-1.2452	-2.3693	0.22303	H	0.72026	0.95039	-3.1742
C	1.57027	-0.6521	-0.2364	H	2.65123	2.34731	-2.7802
C	1.02868	-0.2908	-1.5335	H	1.80753	4.15074	-1.3902
C	-1.7171	-1.2027	0.67865	H	0.55845	3.52653	-2.4651
C	1.24716	0.84834	-2.2251	H	-0.4582	3.97463	-0.3209
C	2.0509	2.07714	-1.9038	H	-0.4369	2.22219	-0.5226
C	1.15292	3.29369	-1.5732	H	-3.3405	0.37293	2.34849
C	0.20103	3.09867	-0.3833	H	-2.9577	1.20156	0.86305
C	-3.1857	-0.9238	0.68276	H	-5.6865	-0.1033	1.42279
C	-3.6044	0.41454	1.27937	H	-5.3369	1.58841	1.7746
O	-3.9972	-1.7312	0.254	H	-4.8562	1.99074	-0.6609
C	-5.0862	0.75675	1.10257	H	-5.219	0.30015	-1.0044
C	-5.4667	1.13404	-0.3357	H	-7.5594	0.62884	-0.1695
C	-6.9515	1.4853	-0.4929	H	-7.2041	2.31237	0.18633
C	-7.3316	1.86802	-1.9268	H	-8.3975	2.10982	-2.0078
O	0.33367	-3.4674	-1.0907	H	-6.765	2.7437	-2.2676
O	2.46005	-4.1675	-0.6835	H	-7.1221	1.0473	-2.6238
C	1.50347	-4.0905	-1.4154	H	2.31039	-5.24	-3.0238
C	1.41724	-4.6496	-2.8169	H	0.52042	-5.266	-2.931
C	0.91452	3.00198	0.95505	H	1.34552	-3.8292	-3.5394
O	1.82547	3.72141	1.3082	H	0.20796	1.35098	3.63235
O	0.37362	2.03384	1.7261	H	1.83933	1.17217	2.92518
C	0.97443	1.83305	3.02406	H	1.27259	2.78877	3.45779
O	2.96009	1.82833	-0.8144	H	4.92038	1.25187	0.70687
O	4.40711	3.35738	-1.6559	H	5.86609	2.74853	0.40555
C	4.09991	2.58169	-0.7799	H	4.32824	2.80432	1.31434
C	4.86562	2.32252	0.48979				

Conformer 4							
Atomic	Standard Orientation (Å)			Atomic	Standard Orientation (Å)		
Type	X	Y	Z	Type	X	Y	Z
C	-0.5509	-4.0707	-1.2026	H	0.02229	-4.9914	-1.248
C	-0.2338	-3.015	-0.146	H	-1.9123	-4.2388	-2.8876
C	-1.291	-1.9595	-0.4098	H	1.95059	-3.2919	-0.0134
C	-2.0737	-2.3757	-1.6128	H	-2.2994	-0.1715	-0.19
C	-1.5285	-3.6913	-2.0348	H	-0.212	-1.0294	1.95624
O	-2.9756	-1.7504	-2.1509	H	0.9237	-0.6308	-1.15
C	1.20151	-2.5639	-0.3222	H	-0.2273	1.25021	2.69552
C	-1.5057	-0.7909	0.2235	H	-1.6046	1.89781	0.04309
C	-0.7908	-0.3032	1.39135	H	-3.3982	1.61981	1.84241
C	1.62098	-1.4029	-0.8376	H	-2.5933	2.89251	2.75882
C	-0.7817	0.98025	1.79773	H	-4.4252	3.93297	1.55746
C	-1.4584	2.12864	1.09775	H	-2.949	4.48294	0.80231
C	-2.791	2.52465	1.74456	H	3.07102	0.18032	-2.6502
C	-3.5666	3.59315	0.96432	H	2.80724	1.01158	-1.1427
C	3.08221	-1.1095	-0.9768	H	5.49671	-0.2637	-1.9368
C	3.42717	0.23135	-1.6082	H	5.09495	1.42234	-2.2596
O	3.9345	-1.91	-0.6189	H	4.82725	1.82634	0.20702
C	4.91535	0.58947	-1.5665	H	5.2409	0.1423	0.52136
C	5.41803	0.97672	-0.1689	H	7.49424	0.49644	-0.5179
C	6.90633	1.34652	-0.1443	H	7.08806	2.17397	-0.8455
C	7.40755	1.73925	1.24923	H	8.47348	1.99442	1.23545
O	-0.2365	-3.5704	1.21013	H	6.86227	2.60914	1.63666
O	-2.4215	-4.1988	1.10461	H	7.27043	0.91872	1.96421
C	-1.3771	-4.1298	1.70633	H	-2.037	-5.1229	3.47799
C	-1.1336	-4.6356	3.10994	H	-0.2953	-5.3394	3.12102
C	-4.1278	3.19617	-0.3959	H	-0.8684	-3.802	3.76878
O	-4.6424	3.98214	-1.1601	H	-4.2223	0.38132	-2.0083
O	-4.0074	1.8743	-0.6502	H	-5.6245	1.50945	-1.9182
C	-4.5348	1.42125	-1.9175	H	-4.1279	2.02656	-2.7306
O	-0.5979	3.30469	1.17367	H	2.10309	4.61446	-0.1681
O	0.71597	2.46281	-0.4707	H	0.61973	5.48242	0.32606
C	0.46021	3.3398	0.32669	H	1.60768	4.68354	1.54999
C	1.25426	4.61057	0.51627				

Conformer 5							
Atomic	Standard Orientation (Å)			Atomic	Standard Orientation (Å)		
Type	X	Y	Z	Type	X	Y	Z
C	0.00029	-3.6393	1.02721	H	-0.4751	-4.5407	0.65351
C	0.18184	-2.429	0.11428	H	0.38181	-4.0944	3.11678
C	0.92711	-1.4535	1.00443	H	-1.6491	-2.5962	-1.1094
C	1.03562	-2.0586	2.36815	H	1.83281	0.3059	1.57067
C	0.43153	-3.4146	2.27418	H	1.11987	-0.1995	-1.4217
O	1.51681	-1.5324	3.35772	H	-1.4174	-0.2073	0.80735
C	-1.1775	-1.9596	-0.3621	H	1.55193	2.10061	-1.8283
C	1.38331	-0.2172	0.72928	H	1.60654	2.45319	1.23184
C	1.34787	0.43388	-0.5679	H	3.4106	3.79288	-0.8657
C	-1.8436	-0.8837	0.07184	H	3.18798	4.34425	0.7981
C	1.57198	1.74141	-0.8007	H	5.23605	3.1113	0.77501
C	1.80995	2.81801	0.22236	H	4.09096	2.03979	1.57518
C	3.20373	3.45667	0.15594	H	-3.9228	0.49564	1.24877
C	4.32693	2.51657	0.61899	H	-3.1593	1.50601	0.05997
C	-3.2014	-0.5517	-0.4613	H	-5.4982	2.03861	-0.1093
C	-3.8647	0.66742	0.16192	H	-5.1785	1.01467	-1.5077
O	-3.7245	-1.2118	-1.3479	H	-6.104	-0.9599	-0.2749
C	-5.2412	1.01438	-0.4122	H	-6.4188	0.05719	1.13002
C	-6.3605	0.06169	0.03056	H	-7.9829	1.46524	-0.2508
C	-7.7322	0.43542	-0.5446	H	-7.6738	0.43755	-1.6422
C	-8.8515	-0.5097	-0.0959	H	-9.8177	-0.2201	-0.5252
O	0.85798	-2.7916	-1.1369	H	-8.6449	-1.5415	-0.4054
O	2.79603	-3.391	-0.1058	H	-8.9585	-0.5078	0.99604
C	2.13401	-3.2606	-1.1095	H	3.57645	-4.0535	-2.4727
C	2.59837	-3.5729	-2.5143	H	1.87918	-4.2238	-3.0203
C	4.68116	1.45749	-0.4121	H	2.66808	-2.6483	-3.098
O	4.821	1.67436	-1.5976	H	4.93138	-1.7471	-0.1823
O	4.84287	0.25084	0.16467	H	4.66477	-0.7529	-1.6552
C	5.20486	-0.8337	-0.7096	H	6.28051	-0.806	-0.909
O	0.88013	3.913	-0.0458	H	-2.2418	4.83934	0.36672
O	-0.8262	2.74554	0.889	H	-0.7708	5.8604	0.30154
C	-0.4098	3.73733	0.32877	H	-1.3088	5.00813	-1.1466
C	-1.2412	4.93922	-0.0552				

Conformer 6							
Atomic	Standard Orientation (Å)			Atomic	Standard Orientation (Å)		
Type	X	Y	Z	Type	X	Y	Z
C	4.5987	-1.2215	-0.9099	H	5.56908	-0.7939	-0.678
C	3.39818	-0.9431	-0.0075	H	4.94053	-2.2655	-2.7839
C	2.30344	-1.7694	-0.6547	H	3.86916	1.12347	0.60808
C	2.85428	-2.372	-1.9095	H	0.41347	-2.5241	-0.966
C	4.28223	-1.9644	-1.9775	H	1.08476	-1.0306	1.68672
O	2.24033	-3.0534	-2.7133	H	1.42606	0.69464	-1.2041
C	3.14964	0.55083	0.0243	H	-1.2548	-0.8638	2.09073
C	1.0188	-1.927	-0.2863	H	-1.6342	-1.807	-0.8176
C	0.40608	-1.3874	0.91593	H	-2.2346	-3.6727	0.71363
C	2.17921	1.21231	-0.6167	H	-3.2634	-2.6006	1.66268
C	-0.9166	-1.2779	1.14166	H	-3.7313	-3.1755	-1.33
C	-2.0252	-1.617	0.18426	H	-4.4853	-4.081	-0.0092
C	-2.8868	-2.7937	0.65237	H	1.2587	3.17555	-2.4178
C	-4.0659	-3.1074	-0.2909	H	0.05469	2.75353	-1.2337
C	2.08034	2.70245	-0.5299	H	1.71904	5.34442	-1.1323
C	0.9771	3.3378	-1.3645	H	0.13597	5.24314	-1.9015
O	2.85225	3.36081	0.15293	H	-0.8861	4.58787	0.30083
C	0.75245	4.82775	-1.093	H	0.69906	4.70234	1.06255
C	0.07956	5.11508	0.25631	H	0.81496	7.13433	0.46621
C	-0.1501	6.61025	0.50958	H	-0.7604	7.02832	-0.3041
C	-0.8259	6.8978	1.85413	H	-0.9732	7.97309	2.00763
O	3.67453	-1.2612	1.39584	H	-1.8097	6.41539	1.91525
O	4.06039	-3.4633	0.96979	H	-0.2215	6.52162	2.68869
C	3.99576	-2.5398	1.74466	H	4.55314	-3.6335	3.49226
C	4.24652	-2.6195	3.23343	H	5.02154	-1.905	3.52811
C	-5.1973	-2.0957	-0.2446	H	3.33562	-2.3576	3.78219
O	-5.6252	-1.4689	-1.1901	H	-7.1136	-1.1724	2.19979
O	-5.7106	-2.0057	1.00356	H	-6.4499	-0.0546	0.96432
C	-6.7916	-1.0743	1.16259	H	-7.6108	-1.3139	0.47988
O	-2.9228	-0.4723	0.0926	H	-3.3829	2.3607	-1.5079
O	-1.5431	0.54525	-1.3915	H	-4.5947	1.0739	-1.2082
C	-2.582	0.52257	-0.7672	H	-3.8942	1.95153	0.15828
C	-3.6812	1.55273	-0.8391				

Conformer 7							
Atomic	Standard Orientation (Å)			Atomic	Standard Orientation (Å)		
Type	X	Y	Z	Type	X	Y	Z
C	-4.1155	-1.3668	-1.1011	H	-4.8782	-2.1311	-0.991
C	-3.0264	-1.2049	-0.0427	H	-4.5706	-0.4683	-3.0262
C	-2.2469	-0.0067	-0.5545	H	-2.7625	-3.302	0.59106
C	-2.8148	0.38317	-1.8801	H	-0.8066	1.47368	-0.5675
C	-3.9605	-0.5277	-2.1325	H	-1.064	-0.4442	1.88475
O	-2.4106	1.27	-2.6179	H	-0.5023	-2.0034	-1.0258
C	-2.2524	-2.5025	0.05554	H	0.86816	0.65125	2.76324
C	-1.2019	0.63604	0.00455	H	1.30279	1.88629	0.02632
C	-0.5943	0.33131	1.28584	H	-0.3033	3.45788	1.23786
C	-1.056	-2.7641	-0.4823	H	0.8915	3.60154	2.52606
C	0.50233	0.9404	1.7793	H	1.14001	5.53324	1.07206
C	1.2916	2.0338	1.10736	H	2.55734	4.53281	0.84813
C	0.76947	3.43675	1.44868	H	1.11238	-5.3882	-1.0597
C	1.47586	4.56261	0.68369	H	0.90823	-3.9009	-2.0119
C	-0.4397	-4.1218	-0.3514	H	1.8513	-2.5486	-0.0982
C	0.92423	-4.3121	-0.9922	H	2.05035	-4.0291	0.83367
O	-0.998	-5.022	0.25871	H	3.63292	-4.9102	-0.9127
C	2.05004	-3.6251	-0.188	H	3.41922	-3.4373	-1.8504
C	3.43118	-3.8324	-0.8238	H	4.35277	-2.086	0.0403
C	4.56461	-3.1601	-0.0385	H	4.57361	-3.5529	0.98871
C	5.94153	-3.3727	-0.6765	H	6.73242	-2.8854	-0.0942
O	-3.5778	-1.053	1.30501	H	6.18958	-4.4394	-0.7449
O	-4.7211	0.84429	0.78309	H	5.97524	-2.9599	-1.6923
C	-4.3985	-6E-05	1.58344	H	-5.535	0.75484	3.22625
C	-4.8268	-0.0513	3.03216	H	-5.2844	-1.0187	3.26146
C	1.26008	4.6142	-0.8235	H	-3.9535	0.05977	3.68387
O	1.93219	5.28426	-1.5759	H	-0.8823	3.15713	-2.7773
O	0.21689	3.85536	-1.2251	H	-0.407	4.88878	-2.9276
C	-0.0842	3.88534	-2.638	H	0.80286	3.61628	-3.2164
O	2.6698	1.99136	1.5743	H	5.50513	0.3852	1.13445
O	3.10272	0.24729	0.19464	H	5.28655	2.1408	1.39649
C	3.47472	1.04768	1.02549	H	4.84826	1.00918	2.67681
C	4.87174	1.14632	1.59069				

Conformer 8							
Atomic	Standard Orientation (Å)			Atomic	Standard Orientation (Å)		
Type	X	Y	Z	Type	X	Y	Z
C	1.91187	3.57864	-1.3514	H	1.5588	4.60285	-1.4165
C	1.23434	2.59467	-0.3984	H	3.51395	3.49304	-2.8141
C	2.05703	1.3266	-0.5995	H	-0.3184	1.90785	-1.7963
C	3.0904	1.60115	-1.6394	H	2.59602	-0.6551	-0.4132
C	2.89789	3.01684	-2.0602	H	0.23289	0.49615	1.2488
O	3.9169	0.81251	-2.0706	H	-1.2214	3.15135	0.8569
C	-0.1965	2.37877	-0.823	H	-0.1828	-1.6149	2.08848
C	1.90004	0.0984	-0.0635	H	1.73955	-3.1824	2.1568
C	0.89452	-0.2933	0.90226	H	0.92551	-4.8979	0.74778
C	-1.2956	2.67982	-0.1176	H	-0.4623	-4.0369	1.41456
C	0.65661	-1.5252	1.40143	H	0.59376	-3.3834	-1.3896
C	1.33015	-2.8504	1.19453	H	-0.4979	-4.722	-1.1237
C	0.36851	-3.956	0.70547	H	-3.5279	2.84867	1.27587
C	-0.1803	-3.7551	-0.7109	H	-4.1589	3.70899	-0.1181
C	-2.6475	2.3175	-0.6266	H	-5.915	2.19654	0.55584
C	-3.8447	2.7135	0.23364	H	-5.2187	1.53441	-0.9227
O	-2.8	1.7331	-1.6929	H	-3.8222	-0.0551	0.48174
C	-5.0157	1.72585	0.13731	H	-4.5686	0.60328	1.93152
C	-4.7457	0.40066	0.864	H	-6.8214	-0.168	1.10239
C	-5.8884	-0.6133	0.72727	H	-6.0584	-0.8192	-0.3394
C	-5.6085	-1.928	1.46357	H	-6.4268	-2.6447	1.32711
O	1.18238	3.09482	0.96535	H	-4.6836	-2.3907	1.10052
O	3.44651	3.16713	1.18526	H	-5.4927	-1.7587	2.54159
C	2.34252	3.32978	1.64405	H	2.96269	4.05352	3.5544
C	2.03096	3.81111	3.04249	H	1.38116	4.69136	3.00877
C	-1.3897	-2.8386	-0.7976	H	1.49869	3.03034	3.59629
O	-2.0718	-2.478	0.14172	H	-2.9206	-1.6956	-3.4105
O	-1.6354	-2.5144	-2.0787	H	-3.6693	-2.0954	-1.8291
C	-2.7882	-1.6851	-2.3282	H	-2.6104	-0.6641	-1.9829
O	2.43634	-2.7411	0.27096	H	5.55934	-3.6453	-0.187
O	3.68243	-4.1708	1.51691	H	4.29701	-3.7068	-1.4442
C	3.56553	-3.4513	0.55093	H	4.72217	-2.1523	-0.7291
C	4.60992	-3.2195	-0.5136				

Conformer 9							
Atomic	Standard Orientation (Å)			Atomic	Standard Orientation (Å)		
Type	X	Y	Z	Type	X	Y	Z
C	0.20674	3.58889	1.11238	H	0.94351	4.35221	0.90119
C	-0.0775	2.48282	0.09387	H	-0.4799	4.08757	3.11072
C	-1.1603	1.67605	0.78644	H	1.88176	2.26975	-0.9089
C	-1.4163	2.27631	2.12798	H	-2.5921	0.2269	1.06645
C	-0.5156	3.45144	2.23356	H	-1.1113	0.43604	-1.6579
O	-2.2229	1.88568	2.96056	H	0.94733	-0.0125	0.92365
C	1.19794	1.74288	-0.2477	H	-2.3624	-1.3693	-2.4215
C	-1.8832	0.62491	0.35082	H	-4.5183	-1.8184	-1.3644
C	-1.8164	0.00081	-0.9547	H	-4.0882	-3.9063	-0.2425
C	1.57464	0.55448	0.2409	H	-3.017	-3.7589	-1.6335
C	-2.5508	-1.0416	-1.3994	H	-2.1396	-3.1511	1.25586
C	-3.6155	-1.8824	-0.7455	H	-1.9739	-4.7265	0.48617
C	-3.2164	-3.3677	-0.6307	H	3.26155	-0.9825	1.75078
C	-2.0169	-3.6464	0.29009	H	2.56193	-2.0119	0.53425
C	2.90257	-0.0321	-0.1267	H	4.8347	-2.7821	0.77184
C	3.33513	-1.244	0.68336	H	4.81195	-1.8852	-0.7456
O	3.57921	0.43387	-1.0329	H	5.77296	0.11306	0.4163
C	4.73012	-1.776	0.34322	H	5.79081	-0.7728	1.94001
C	5.87681	-0.8897	0.84862	H	7.36423	-2.4586	0.93089
C	7.26483	-1.4474	0.51002	H	7.35075	-1.5613	-0.5799
C	8.40954	-0.567	1.02182	H	9.38744	-0.9886	0.76185
O	-0.6785	3.0054	-1.1482	H	8.35556	0.44054	0.59161
O	0.97516	4.52457	-1.5234	H	8.37175	-0.4621	2.11334
C	-0.0657	3.99514	-1.846	H	-0.3908	5.16149	-3.6055
C	-0.8708	4.33605	-3.0789	H	-0.9349	3.46347	-3.7374
C	-0.6492	-3.2587	-0.2427	H	-1.8928	4.61029	-2.7994
O	0.24057	-2.7759	0.42646	H	0.70159	-3.6009	-3.1706
O	-0.509	-3.5775	-1.5485	H	0.97925	-2.2087	-2.0728
C	0.77187	-3.2804	-2.1309	H	1.56445	-3.828	-1.6147
O	-3.9687	-1.4262	0.57736	H	-6.3394	0.06973	2.30061
O	-5.9847	-0.6687	-0.1498	H	-5.221	-1.215	2.85147
C	-5.1823	-0.816	0.74223	H	-4.5798	0.37265	2.43064
C	-5.3481	-0.366	2.17204				

Conformer 10							
Atomic	Standard Orientation (Å)			Atomic	Standard Orientation (Å)		
Type	X	Y	Z	Type	X	Y	Z
C	-0.1504	3.69269	0.88031	H	0.34934	4.63124	0.66195
C	-0.2233	2.59331	-0.177	H	-0.7553	3.91151	2.95551
C	-1.0275	1.51498	0.5249	H	1.69828	2.91918	-1.2121
C	-1.316	1.98031	1.91575	H	-1.8834	-0.3397	0.81007
C	-0.7063	3.32962	2.04262	H	-0.9046	0.52095	-2.0224
O	-1.9352	1.3718	2.77506	H	1.38312	0.34589	0.43459
C	1.18471	2.20073	-0.5746	H	-1.1342	-1.7488	-2.6949
C	-1.3875	0.29593	0.07942	H	-1.4408	-2.4388	0.2891
C	-1.1807	-0.2104	-1.2669	H	-3.0776	-3.6014	-2.0217
C	1.84651	1.10319	-0.1914	H	-3.0404	-4.1916	-0.3566
C	-1.2948	-1.4979	-1.6464	H	-4.0932	-1.4227	-1.2167
C	-1.5848	-2.6805	-0.7631	H	-5.0894	-2.8166	-0.8614
C	-2.9788	-3.2902	-0.9748	H	3.84134	-0.3493	1.01624
C	-4.1294	-2.3418	-0.6216	H	3.24718	-1.2422	-0.3446
C	3.2548	0.85951	-0.6362	H	5.60438	-1.6872	-0.3373
C	3.90383	-0.3989	-0.0815	H	5.36341	-0.542	-1.6482
O	3.8252	1.61857	-1.407	H	7.35754	0.09831	-0.4603
C	5.33994	-0.6451	-0.5569	H	6.13133	1.33046	-0.2158
C	6.39978	0.29958	0.04102	H	7.32766	0.97525	1.85942
C	6.61631	0.1933	1.56096	H	5.68354	0.42629	2.09344
C	7.14554	-1.1655	2.03544	H	7.34238	-1.1581	3.11375
O	-0.7963	3.07844	-1.4364	H	6.43293	-1.9749	1.83918
O	-2.7996	3.60499	-0.4927	H	8.08411	-1.4217	1.52763
C	-2.0727	3.55676	-1.4557	H	-3.4394	4.42466	-2.8486
C	-2.4332	4.00447	-2.8541	H	-1.7161	4.75022	-3.2116
C	-4.1591	-1.9563	0.84904	H	-2.3907	3.15458	-3.5435
O	-3.4792	-2.4685	1.71632	H	-5.8744	0.28477	2.4282
O	-5.0327	-0.9607	1.07097	H	-5.306	-1.2711	3.12322
C	-5.0792	-0.4609	2.42614	H	-4.1217	-0.0043	2.68925
O	-0.6518	-3.7507	-1.105	H	2.46637	-4.7026	-0.6991
O	1.02389	-2.6791	-0.0143	H	1.00655	-5.7369	-0.7001
C	0.62088	-3.6228	-0.6608	H	1.47278	-4.8883	-2.1749
C	1.45156	-4.8127	-1.0826				

Conformer 11							
Atomic	Standard Orientation (Å)			Atomic	Standard Orientation (Å)		
Type	X	Y	Z	Type	X	Y	Z
C	-0.1504	3.69269	0.88031	H	0.34934	4.63124	0.66195
C	-0.2233	2.59331	-0.177	H	-0.7553	3.91151	2.95551
C	-1.0275	1.51498	0.5249	H	1.69828	2.91918	-1.2121
C	-1.316	1.98031	1.91575	H	-1.8834	-0.3397	0.81007
C	-0.7063	3.32962	2.04262	H	-0.9046	0.52095	-2.0224
O	-1.9352	1.3718	2.77506	H	1.38312	0.34589	0.43459
C	1.18471	2.20073	-0.5746	H	-1.1342	-1.7488	-2.6949
C	-1.3875	0.29593	0.07942	H	-1.4408	-2.4388	0.2891
C	-1.1807	-0.2104	-1.2669	H	-3.0776	-3.6014	-2.0217
C	1.84651	1.10319	-0.1914	H	-3.0404	-4.1916	-0.3566
C	-1.2948	-1.4979	-1.6464	H	-4.0932	-1.4227	-1.2167
C	-1.5848	-2.6805	-0.7631	H	-5.0894	-2.8166	-0.8614
C	-2.9788	-3.2902	-0.9748	H	3.84134	-0.3493	1.01624
C	-4.1294	-2.3418	-0.6216	H	3.24718	-1.2422	-0.3446
C	3.2548	0.85951	-0.6362	H	5.60438	-1.6872	-0.3373
C	3.90383	-0.3989	-0.0815	H	5.36341	-0.542	-1.6482
O	3.8252	1.61857	-1.407	H	7.35754	0.09831	-0.4603
C	5.33994	-0.6451	-0.5569	H	6.13133	1.33046	-0.2158
C	6.39978	0.29958	0.04102	H	7.32766	0.97525	1.85942
C	6.61631	0.1933	1.56096	H	5.68354	0.42629	2.09344
C	7.14554	-1.1655	2.03544	H	7.34238	-1.1581	3.11375
O	-0.7963	3.07844	-1.4364	H	6.43293	-1.9749	1.83918
O	-2.7996	3.60499	-0.4927	H	8.08411	-1.4217	1.52763
C	-2.0727	3.55676	-1.4557	H	-3.4394	4.42466	-2.8486
C	-2.4332	4.00447	-2.8541	H	-1.7161	4.75022	-3.2116
C	-4.1591	-1.9563	0.84904	H	-2.3907	3.15458	-3.5435
O	-3.4792	-2.4685	1.71632	H	-5.8744	0.28477	2.4282
O	-5.0327	-0.9607	1.07097	H	-5.306	-1.2711	3.12322
C	-5.0792	-0.4609	2.42614	H	-4.1217	-0.0043	2.68925
O	-0.6518	-3.7507	-1.105	H	2.46637	-4.7026	-0.6991
O	1.02389	-2.6791	-0.0143	H	1.00655	-5.7369	-0.7001
C	0.62088	-3.6228	-0.6608	H	1.47278	-4.8883	-2.1749
C	1.45156	-4.8127	-1.0826				

Conformer 12							
Atomic	Standard Orientation (Å)			Atomic	Standard Orientation (Å)		
Type	X	Y	Z	Type	X	Y	Z
C	0.09782	3.57189	1.25349	H	0.72602	4.44535	1.14154
C	-0.0394	2.56369	0.11265	H	-0.6463	3.74085	3.28646
C	-0.9929	1.53703	0.69759	H	1.95014	2.74887	-0.8369
C	-1.3302	1.945	2.09314	H	-2.1047	-0.1947	0.79698
C	-0.5978	3.2116	2.34155	H	-0.9118	0.73363	-1.9249
O	-2.0756	1.36161	2.86702	H	1.23421	0.07969	0.5059
C	1.31946	2.04147	-0.3036	H	-1.3949	-1.4369	-2.7752
C	-1.4925	0.41759	0.13752	H	-1.8982	-2.2978	0.13721
C	-1.2982	-0.0126	-1.2365	H	-3.5763	-3.0602	-2.3077
C	1.81122	0.82591	-0.033	H	-3.6773	-3.7817	-0.6979
C	-1.5598	-1.2443	-1.7151	H	-4.3378	-0.8391	-1.3449
C	-2.0296	-2.4413	-0.9345	H	-5.5167	-2.1169	-1.1483
C	-3.4798	-2.8491	-1.2359	H	3.58265	-0.9455	1.09506
C	-4.5156	-1.7931	-0.8361	H	2.91048	-1.6643	-0.331
C	3.18626	0.43499	-0.4782	H	5.19327	-2.4016	-0.3249
C	3.66125	-0.9295	-0.0027	H	5.12374	-1.1508	-1.5574
O	3.86264	1.16396	-1.1895	H	7.15696	-0.8455	-0.3049
C	5.06507	-1.3223	-0.4761	H	6.08845	0.51144	0.00874
C	6.2216	-0.5596	0.19764	H	7.18845	-0.1272	2.06898
C	6.39231	-0.793	1.70917	H	5.48478	-0.4816	2.24513
C	6.74007	-2.2357	2.0957	H	6.91414	-2.3247	3.17429
O	-0.7157	3.14293	-1.0656	H	5.9373	-2.9353	1.83563
O	0.71834	4.90198	-1.2463	H	7.65015	-2.5722	1.58291
C	-0.2412	4.2773	-1.6414	H	-0.747	5.6001	-3.2399
C	-1.0808	4.64022	-2.8446	H	-0.9776	3.86944	-3.616
C	-4.5601	-1.5345	0.66144	H	-2.1389	4.6906	-2.5704
O	-3.9829	-2.1977	1.4999	H	-6.0762	0.74005	2.38972
O	-5.3191	-0.4626	0.94628	H	-5.7085	-0.9258	2.95058
C	-5.3667	-0.0863	2.34067	H	-4.3765	0.23378	2.67494
O	-1.2265	-3.5943	-1.3305	H	1.72719	-4.9676	-0.8997
O	0.52384	-2.8303	-0.1069	H	0.14991	-5.8017	-1.0328
C	0.03308	-3.6645	-0.8375	H	0.78361	-4.9121	-2.4183
C	0.72431	-4.9167	-1.3248				

Conformer 13							
Atomic	Standard Orientation (Å)			Atomic	Standard Orientation (Å)		
Type	X	Y	Z	Type	X	Y	Z
C	0.57209	3.68637	0.62326	H	1.26914	4.43822	0.26671
C	0.14454	2.53642	-0.2882	H	0.21982	4.26258	2.68761
C	-0.8608	1.79589	0.57226	H	1.985	2.23071	-1.4619
C	-0.8932	2.44569	1.91781	H	-2.2613	0.38243	1.10415
C	0.04382	3.59764	1.85017	H	-1.112	0.44409	-1.7851
O	-1.5622	2.09371	2.87748	H	1.25632	0.08476	0.61069
C	1.37962	1.75779	-0.6898	H	-2.4138	-1.4064	-2.3058
C	-1.6383	0.73262	0.29057	H	-4.4399	-1.7849	-0.9307
C	-1.725	0.04234	-0.9813	H	-3.8861	-3.8714	0.08154
C	1.81448	0.60846	-0.1608	H	-3.0171	-3.7368	-1.4437
C	-2.4855	-1.0315	-1.2854	H	-1.7387	-3.1737	1.30282
C	-3.4463	-1.8533	-0.4713	H	-1.6967	-4.7379	0.49237
C	-3.065	-3.3483	-0.4211	H	3.56899	-0.9508	1.23283
C	-1.7543	-3.6541	0.32196	H	2.79189	-1.972	0.0636
C	3.1058	0.00336	-0.6186	H	5.0631	-2.7663	0.1528
C	3.57938	-1.2104	0.16389	H	4.92789	-1.8806	-1.364
O	3.72108	0.45733	-1.5738	H	7.04238	-1.3068	-0.39
C	4.94084	-1.7614	-0.2743	H	6.01883	0.11439	-0.2872
C	6.15447	-0.898	0.11328	H	5.57837	-0.3988	2.14959
C	6.43981	-0.8287	1.62018	H	6.55966	-1.8486	2.01402
C	7.6888	-0.0044	1.95081	H	7.87401	0.02955	3.03065
O	-0.3741	3.00571	-1.5757	H	8.58054	-0.4281	1.47243
O	-2.0593	4.20832	-0.6298	H	7.58549	1.02884	1.59713
C	-1.4678	3.81886	-1.6075	H	-2.6436	4.86419	-3.0519
C	-1.8253	4.14354	-3.0402	H	-0.9575	4.5482	-3.57
C	-0.4674	-3.2653	-0.3832	H	-2.132	3.23106	-3.5629
O	0.50242	-2.7854	0.16591	H	0.49226	-3.5933	-3.4627
O	-0.4989	-3.5781	-1.6978	H	0.90832	-2.2054	-2.4033
C	0.69581	-3.2771	-2.4393	H	1.5488	-3.8258	-2.0319
O	-3.5597	-1.3571	0.87907	H	-5.741	-0.4974	3.18022
O	-5.8167	-1.5966	0.83781	H	-4.2593	-1.4438	3.48003
C	-4.8096	-1.2631	1.41925	H	-4.1184	0.19704	2.85114
C	-4.7335	-0.7083	2.82023				

Conformer 14							
Atomic	Standard Orientation (Å)			Atomic	Standard Orientation (Å)		
Type	X	Y	Z	Type	X	Y	Z
C	-0.4847	-3.5337	1.53607	H	-1.2555	-4.2943	1.60691
C	-0.5688	-2.4572	0.4549	H	0.82079	-3.9631	3.21758
C	0.72644	-1.6809	0.66712	H	-1.7419	-1.044	1.66854
C	1.39623	-2.2255	1.88449	H	2.20092	-0.2786	0.31799
C	0.56183	-3.3636	2.35273	H	-0.3249	-0.3887	-1.4953
O	2.42366	-1.8113	2.40167	H	-2.9642	-2.0281	-0.9658
C	-1.7806	-1.6014	0.73476	H	0.56759	1.33736	-2.7698
C	1.24995	-0.6572	-0.0377	H	2.92984	1.73898	-2.8821
C	0.65996	-0.0304	-1.2048	H	3.33783	3.61685	-1.4335
C	-2.8763	-1.489	-0.0273	H	1.68891	3.6748	-2.0557
C	1.18706	0.96072	-1.9556	H	1.6148	4.33954	0.2599
C	2.50072	1.6876	-1.8748	H	0.86209	2.74705	0.18595
C	2.35477	3.14103	-1.3666	H	-4.8472	-0.402	-1.5659
C	1.80996	3.2772	0.06249	H	-5.6212	-1.6033	-0.5623
C	-4.0093	-0.6131	0.39569	H	-7.2085	0.19576	-0.7203
C	-5.211	-0.5809	-0.5424	H	-6.5344	0.30074	0.90525
O	-3.9807	0.01986	1.44103	H	-4.9896	2.12208	0.15126
C	-6.295	0.42962	-0.1571	H	-5.6499	2.01664	-1.4801
C	-5.9	1.89014	-0.4152	H	-7.9187	2.64758	-0.592
C	-7.0015	2.88745	-0.0349	H	-7.2489	2.76131	1.02842
C	-6.6109	4.34533	-0.2979	H	-7.4153	5.03326	-0.0135
O	-0.7521	-3.0045	-0.8761	H	-5.7167	4.62396	0.27298
O	1.17969	-4.2041	-0.8025	H	-6.3904	4.51196	-1.3598
C	0.19064	-3.8458	-1.3931	H	0.52192	-4.9814	-3.1701
C	-0.1894	-4.2421	-2.8008	H	-1.2043	-4.6511	-2.8248
C	2.79244	2.815	1.12727	H	-0.1737	-3.3611	-3.4516
O	3.97902	3.07479	1.13155	H	2.31907	1.6152	4.05259
O	2.16026	2.11007	2.0846	H	3.23494	0.56047	2.94007
C	2.95943	1.59286	3.16916	H	3.8478	2.20889	3.315
O	3.43478	0.9717	-1.043	H	5.20304	-0.5398	-0.0252
O	5.15314	1.68184	-2.3413	H	6.63189	0.31833	-0.6955
C	4.7587	1.09421	-1.3603	H	5.6236	1.07286	0.57183
C	5.6155	0.4305	-0.316				

Table S3. Energy analyses of 4*S*,12*S*-**1**/4*R*,12*R*-**1** (ten conformers)

**NO.**	**3D conformers** B3LYP/6-31G(d,p)	**G (Hartree)**	**Boltzmann distribution**	**Calculated ECD spectrum** 4*S*,12*S*-**1**
1	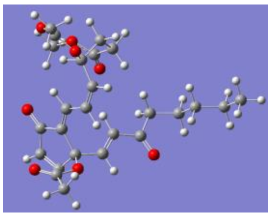	-1573.242744	5.14 %	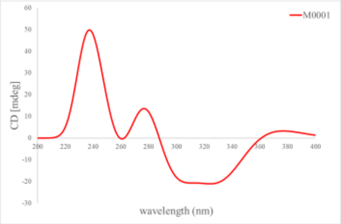
2	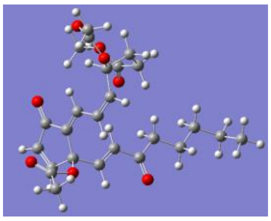	-1573.242248	3.04 %	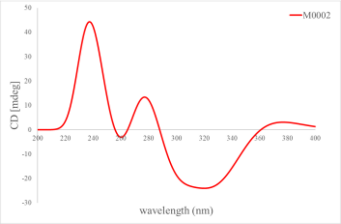
3	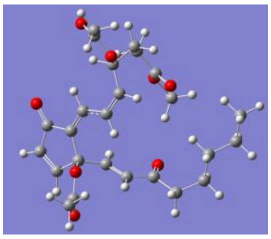	-1573.241432	1.28 %	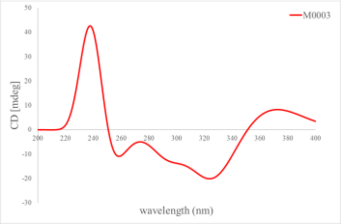
4	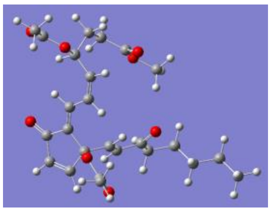	-1573.244033	20.12 %	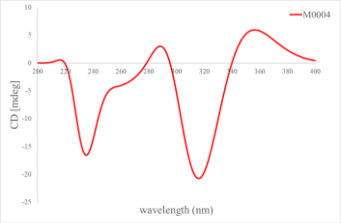

5	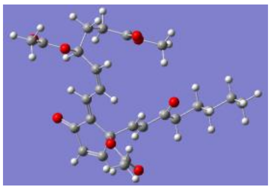	-1573.245111	63.01 %	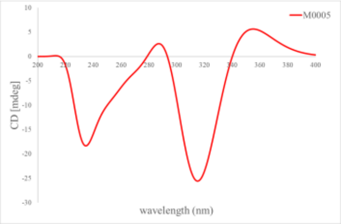
6	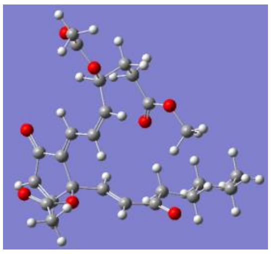	-1573.241519	1.4 %	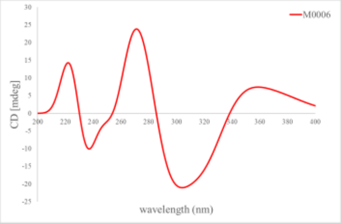
7	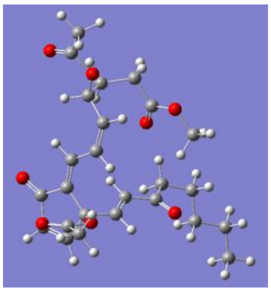	-1573.242182	2.83 %	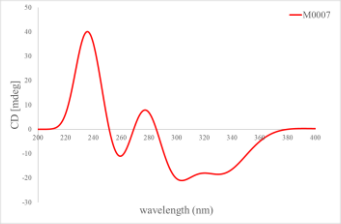
8	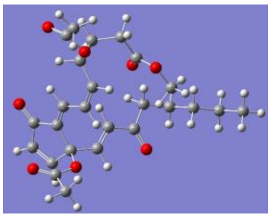	-1573.241161	0.96 %	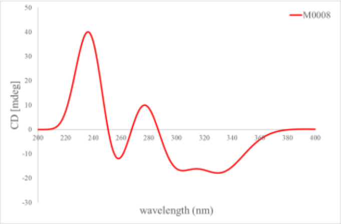
9	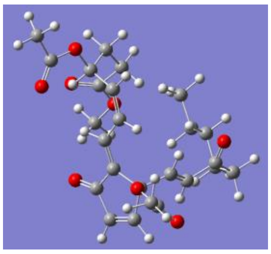	-1573.241458	1.32 %	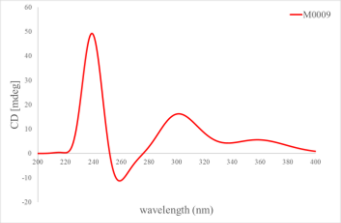

10	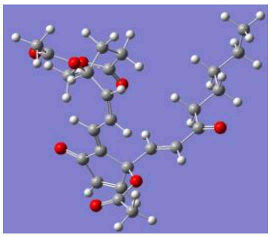	-1573.240815	0.67 %	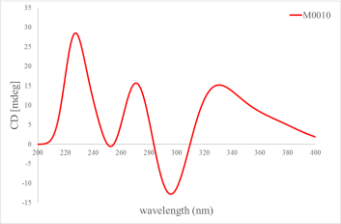

Table S4. Cartesian coordinates of the low-energy re-optimized conformers of 4*S*,12*S*-**1**/4*R*,12*R*-**1** calculated at B3LYP/6-31G(d,p) level of theory.

Conformer 1							
Atomic	Standard Orientation (Å)			Atomic	Standard Orientation (Å)		
Type	X	Y	Z	Type	X	Y	Z
C	-0.0539	-4.0445	0.93033	H	-0.6445	-4.9314	0.72414
C	-0.0324	-2.8837	-0.0623	H	0.75433	-4.4235	2.91134
C	0.96889	-1.9346	0.57075	H	-2.0853	-2.9052	-0.876
C	1.32944	-2.4739	1.91741	H	2.17669	-0.2782	0.76761
C	0.65284	-3.7891	2.03878	H	0.56759	-0.7462	-1.8654
O	2.02616	-1.922	2.75694	H	-1.3589	-0.6885	1.12947
C	-1.4399	-2.3409	-0.2044	H	1.27761	1.41429	-2.5588
C	1.47409	-0.7773	0.10282	H	2.8039	1.61434	0.12401
C	1.17436	-0.1631	-1.1771	H	2.14547	3.90915	0.309
C	-1.9531	-1.291	0.44718	H	1.262	3.8636	-1.2219
C	1.56918	1.06597	-1.5686	H	-0.2981	3.8983	0.67738
C	2.34356	2.06258	-0.7561	H	-0.4998	2.42127	-0.2457
C	1.50761	3.28441	-0.3232	H	-3.9054	-0.1508	2.15243
C	0.19673	2.95542	0.40667	H	-3.1123	1.02891	1.15307
C	-3.3829	-0.8965	0.24753	H	-5.9534	-0.0397	0.61692
C	-3.881	0.24408	1.12354	H	-5.6297	1.42189	1.54863
O	-4.1035	-1.473	-0.5553	H	-4.5229	2.45089	-0.4616
C	-5.2529	0.79672	0.728	H	-4.8638	0.99038	-1.3879
C	-5.2357	1.6185	-0.5683	H	-7.3222	1.34727	-1.0539
C	-6.6123	2.17931	-0.9462	H	-6.9922	2.79898	-0.121
C	-6.5942	3.00424	-2.2371	H	-7.5911	3.38826	-2.4823
O	0.29722	-3.3109	-1.4215	H	-5.9188	3.86456	-2.1489
O	2.30837	-4.1839	-0.8131	H	-6.2512	2.40107	-3.0868
C	1.4846	-3.9381	-1.6606	H	2.51478	-4.8467	-3.2955
C	1.60886	-4.2622	-3.1316	H	0.73205	-4.8181	-3.4776
C	0.29169	2.13929	1.68634	H	1.65967	-3.3356	-3.7137
O	-0.6137	1.4497	2.11567	H	2.59376	1.9117	3.94936
O	1.47086	2.29303	2.30859	H	0.8228	1.78296	4.23002
C	1.64417	1.56236	3.54466	H	1.68464	0.48841	3.34642
O	3.41168	2.53611	-1.6276	H	6.51092	3.62958	-1.58
O	4.77547	2.85274	0.15896	H	5.79679	2.49297	-2.7619
C	4.5819	2.88886	-1.0355	H	5.18917	4.14691	-2.6707
C	5.58988	3.32038	-2.0751				

Conformer 2							
Atomic	Standard Orientation (Å)			Atomic	Standard Orientation (Å)		
Type	X	Y	Z	Type	X	Y	Z
C	0.15923	-3.9419	1.14684	H	-0.3261	-4.8924	0.94888
C	0.27766	-2.8915	0.04426	H	0.62935	-4.0451	3.26577
C	1.08837	-1.8013	0.72141	H	-1.5958	-3.1683	-1.0923
C	1.24036	-2.1663	2.163	H	2.13482	-0.0392	0.92362
C	0.64145	-3.5148	2.32092	H	1.04807	-0.9104	-1.8693
O	1.73985	-1.4733	3.03778	H	-1.3792	-0.708	0.74181
C	-1.1161	-2.4866	-0.3914	H	1.73159	1.21715	-2.6815
C	1.59328	-0.6611	0.21319	H	2.74107	1.82193	0.17908
C	1.48565	-0.2105	-1.1618	H	1.91817	4.06103	-0.0211
C	-1.8036	-1.4244	0.0429	H	1.32963	3.7847	-1.6653
C	1.86491	0.9994	-1.6225	H	-0.5461	3.88955	-0.0797
C	2.41856	2.13591	-0.8133	H	-0.4867	2.31357	-0.8463
C	1.44456	3.32406	-0.6763	H	-4.0168	-0.2361	1.28742
C	0.04639	2.96698	-0.15	H	-3.2478	0.86762	0.19203
C	-3.2012	-1.1762	-0.4329	H	-5.1498	0.52077	-1.468
C	-3.9192	-0.0032	0.21407	H	-5.9141	-0.5865	-0.3515
O	-3.7273	-1.8795	-1.2836	H	-6.0713	1.26092	1.36945
C	-5.2834	0.31037	-0.4	H	-5.3631	2.3843	0.22058
C	-5.9892	1.48218	0.29546	H	-8.0175	0.89413	-0.175
C	-7.3919	1.79493	-0.2519	H	-7.8612	2.54685	0.39694
C	-7.4103	2.30596	-1.6976	H	-8.4291	2.56039	-2.012
O	0.86724	-3.4262	-1.1824	H	-6.7948	3.20842	-1.8043
O	2.79313	-4.0607	-0.1503	H	-7.0273	1.55817	-2.4008
C	2.11619	-3.9731	-1.146	H	1.75349	-5.0978	-2.9473
C	2.51674	-4.435	-2.5283	H	2.60481	-3.5726	-3.1979
C	-0.0411	2.29713	1.21258	H	3.47494	-4.9523	-2.4706
O	-0.9642	1.58303	1.55507	H	1.82099	2.49683	3.85175
O	0.99389	2.61092	2.00746	H	0.04111	2.24517	3.83244
C	0.98667	2.02765	3.33083	H	1.1343	0.94662	3.26822
O	3.59377	2.60081	-1.5395	H	6.55619	3.953	-1.0947
O	4.59272	3.21715	0.40321	H	6.15002	2.62771	-2.2243
C	4.61469	3.10944	-0.8024	H	5.42541	4.21955	-2.457
C	5.76469	3.50738	-1.698				

Conformer 3							
Atomic	Standard Orientation (Å)			Atomic	Standard Orientation (Å)		
Type	X	Y	Z	Type	X	Y	Z
C	2.06977	-3.6832	-1.2463	H	3.11115	-3.9582	-1.1517
C	1.47851	-2.5831	-0.3587	H	1.34927	-4.9632	-2.8424
C	0.02718	-2.5321	-0.8356	H	2.14115	-0.9149	-1.6213
C	-0.1495	-3.5376	-1.9239	H	-1.931	-1.9126	-0.9483
C	1.17379	-4.1892	-2.1037	H	0.0073	-0.6187	1.12681
O	-1.1755	-3.7629	-2.545	H	2.82596	-0.7644	1.36332
C	2.17157	-1.2648	-0.5905	H	-1.7297	0.8771	1.69148
C	-0.9883	-1.7407	-0.4299	H	-3.5214	-0.6355	-0.3248
C	-0.9322	-0.7178	0.59293	H	-4.6497	1.51353	-0.6815
C	2.75732	-0.4761	0.31901	H	-3.3932	2.3729	0.2246
C	-1.9238	0.13609	0.91918	H	-2.6752	0.73688	-2.2732
C	-3.2989	0.21369	0.32474	H	-3.3281	2.34499	-2.4222
C	-3.583	1.52149	-0.437	H	3.62128	1.36086	2.00838
C	-2.7832	1.68588	-1.7341	H	5.05611	1.37616	0.99874
C	3.30721	0.85303	-0.069	H	4.57991	3.69486	1.47524
C	3.9942	1.66794	1.02251	H	4.12433	3.42482	-0.2066
O	3.22417	1.27228	-1.2172	H	1.70445	3.15389	0.52879
C	3.85496	3.1838	0.82818	H	2.19672	3.48431	2.18415
C	2.44151	3.70009	1.13263	H	3.00802	5.76322	1.46971
C	2.27226	5.20249	0.87488	H	2.50903	5.41368	-0.1779
C	0.85917	5.70414	1.19161	H	0.754	6.77226	0.96794
O	1.44629	-2.9496	1.05427	H	0.10993	5.15638	0.60847
O	3.63933	-3.5605	1.14813	H	0.61928	5.56238	2.25299
C	2.55661	-3.4355	1.67278	H	3.14243	-4.0996	3.61718
C	2.22859	-3.8023	3.10188	H	1.75994	-2.9588	3.61811
C	-1.4044	2.30203	-1.558	H	1.51161	-4.6302	3.11457
O	-0.9778	2.79952	-0.5348	H	1.28922	2.26383	-2.1597
O	-0.729	2.26195	-2.7195	H	0.8726	2.90005	-3.7793
C	0.57977	2.86793	-2.7293	H	0.53546	3.87668	-2.3118
O	-4.2039	0.1461	1.46805	H	-7.2168	-0.8448	2.33015
O	-5.8046	-0.8097	0.17518	H	-5.7151	-1.0258	3.2854
C	-5.4268	-0.4001	1.2508	H	-6.3305	0.5893	2.93187
C	-6.2308	-0.4241	2.52976				

Conformer 4							
Atomic	Standard Orientation (Å)			Atomic	Standard Orientation (Å)		
Type	X	Y	Z	Type	X	Y	Z
C	-0.8213	3.57712	-1.1011	H	-1.6908	4.15912	-0.8227
C	-0.3372	2.44726	-0.2005	H	-0.2333	4.41125	-3.018
C	0.91852	1.96537	-0.9206	H	-1.1925	0.67964	0.82587
C	1.02462	2.70933	-2.2115	H	2.65269	0.91842	-1.2666
C	-0.0843	3.70114	-2.2124	H	1.15039	0.59342	1.44837
O	1.86456	2.54201	-3.0803	H	-2.5459	1.64855	-1.7499
C	-1.342	1.32991	-0.0344	H	2.73355	-1.0607	2.02826
C	1.85263	1.06803	-0.5427	H	4.01535	-0.5989	-0.7533
C	1.90489	0.34042	0.70875	H	4.7248	-2.9146	-0.6563
C	-2.3402	1.03489	-0.8759	H	3.8736	-3.1797	0.87552
C	2.81132	-0.6003	1.04655	H	2.3275	-2.3592	-1.6475
C	3.92784	-1.1312	0.1958	H	2.76814	-4.0235	-1.3597
C	3.83614	-2.6434	-0.0781	H	-4.9118	0.66949	-1.6726
C	2.58264	-3.0682	-0.8511	H	-3.9659	-0.4832	-2.5819
C	-3.2252	-0.138	-0.6283	H	-6.0245	-1.619	-2.0779
C	-4.3993	-0.3005	-1.5855	H	-4.8181	-2.3302	-1.0081
O	-3.0112	-0.9305	0.28045	H	-5.6197	-0.8732	0.86773
C	-5.3833	-1.4123	-1.2107	H	-6.8221	-0.1518	-0.1992
C	-6.2622	-1.0779	0.00236	H	-7.8854	-2.4115	-0.5144
C	-7.2497	-2.1967	0.35685	H	-6.6895	-3.1203	0.56014
C	-8.1327	-1.8602	1.56275	H	-8.8244	-2.6785	1.79361
O	0.08031	2.93815	1.11555	H	-7.5259	-1.6739	2.45723
O	-2.0143	3.66514	1.60993	H	-8.7318	-0.9602	1.37694
C	-0.8553	3.5094	1.92159	H	0.61699	4.57545	3.06908
C	-0.2366	3.91184	3.23922	H	-0.9865	4.41358	3.85144
C	1.36029	-3.2774	0.02906	H	0.13772	3.02631	3.76365
O	1.37131	-3.3597	1.24018	H	-1.7069	-3.9218	-0.7627
O	0.2513	-3.4049	-0.7225	H	-0.8452	-4.4422	0.72548
C	-0.977	-3.6395	-0.0035	H	-1.3017	-2.7276	0.50207
O	5.14636	-0.879	0.95906	H	8.35722	-0.2849	0.58692
O	6.348	-0.7401	-0.9605	H	7.25192	0.48311	1.76582
C	6.28681	-0.6925	0.24856	H	7.57493	-1.2476	1.87721
C	7.44842	-0.4174	1.17477				

Conformer 5							
Atomic	Standard Orientation (Å)			Atomic	Standard Orientation (Å)		
Type	X	Y	Z	Type	X	Y	Z
C	0.63291	3.59675	1.13915	H	1.39186	4.29211	0.80352
C	0.08804	2.54041	0.18565	H	0.3271	4.13163	3.2216
C	-0.9812	1.85326	1.02959	H	0.87874	0.99392	-1.1895
C	-0.9164	2.42192	2.40926	H	-2.5413	0.60458	1.50974
C	0.09489	3.51216	2.3626	H	-1.5205	0.7634	-1.434
O	-1.5751	2.06831	3.37334	H	2.59823	1.79761	1.21925
C	1.13105	1.5555	-0.2915	H	-3.0453	-0.9604	-1.9655
C	-1.8948	0.9196	0.69156	H	-3.8452	-0.9735	1.0268
C	-2.1098	0.3473	-0.6217	H	-4.3773	-3.3194	0.7299
C	2.2855	1.26881	0.32186	H	-3.7947	-3.3075	-0.9439
C	-2.9843	-0.6327	-0.9307	H	-1.8922	-2.6505	1.37503
C	-3.8866	-1.3739	0.01198	H	-2.2463	-4.303	0.93858
C	-3.6298	-2.8912	0.05506	H	5.0368	1.07352	0.45001
C	-2.2293	-3.2792	0.5425	H	4.35211	-0.1026	1.54293
C	3.19986	0.22484	-0.2219	H	4.93419	-1.9555	-0.0809
C	4.54552	0.08786	0.47513	H	5.61247	-0.7779	-1.1816
O	2.89042	-0.4762	-1.1765	H	7.31875	-0.1243	0.56359
C	5.45387	-0.9895	-0.1168	H	6.63667	-1.3066	1.6701
C	6.8038	-1.0961	0.60285	H	7.20715	-3.147	0.05421
C	7.7207	-2.1759	0.0139	H	7.88804	-1.9653	-1.0518
C	9.06946	-2.2806	0.73275	H	9.69989	-3.0594	0.28879
O	-0.5898	3.14117	-0.9664	H	9.62202	-1.3344	0.67927
O	1.32188	4.13141	-1.6919	H	8.93578	-2.5253	1.7938
C	0.14264	3.89623	-1.8294	H	-0.1384	5.03648	-3.6127
C	-0.7259	4.38648	-2.9636	H	-1.1066	3.53566	-3.5385
C	-1.1709	-3.2553	-0.5493	H	-1.5917	4.9283	-2.5703
O	-1.3912	-3.1866	-1.7411	H	2.02776	-3.6889	-0.3768
O	0.06044	-3.3651	-0.0179	H	0.95671	-4.0959	-1.7607
C	1.15585	-3.3843	-0.9564	H	1.31742	-2.3877	-1.3725
O	-5.2375	-1.1544	-0.496	H	-8.3674	-0.9377	0.48261
O	-6.0856	-1.3734	1.59705	H	-7.553	0.07762	-0.745
C	-6.2443	-1.1722	0.41295	H	-7.754	-1.6499	-1.0406
C	-7.5701	-0.9041	-0.2605				

Conformer 6							
Atomic	Standard Orientation (Å)			Atomic	Standard Orientation (Å)		
Type	X	Y	Z	Type	X	Y	Z
C	0.27991	3.83798	1.20683	H	0.96669	4.62023	0.89955
C	-0.0648	2.70305	0.24449	H	-0.1807	4.33777	3.26969
C	-1.0953	1.90631	1.03721	H	1.55822	1.39828	0.8995
C	-1.1955	2.50822	2.40295	H	-2.4269	0.39276	1.46077
C	-0.2966	3.69348	2.406	H	-1.1814	0.66048	-1.3894
O	-1.8731	2.09327	3.32793	H	1.40617	2.15563	-2.0835
C	1.1815	1.87837	0.00023	H	-2.1851	-1.4183	-1.9326
C	-1.7785	0.79961	0.68611	H	-3.3042	-1.4699	0.94841
C	-1.7328	0.14515	-0.6075	H	-2.9323	-3.8752	0.94199
C	1.79597	1.70267	-1.1769	H	-2.0885	-3.8041	-0.6103
C	-2.2958	-1.0404	-0.9177	H	-1.075	-2.3819	1.93493
C	-3.0345	-1.9589	0.01002	H	-0.6296	-4.0206	1.4846
C	-2.2719	-3.2607	0.32178	H	3.95621	1.10356	0.5062
C	-0.9494	-3.0512	1.07864	H	3.054	-0.3932	0.34329
C	2.98984	0.83812	-1.3911	H	4.78512	-1.2695	-1.2634
C	3.74015	0.28028	-0.1897	H	5.68775	0.22041	-1.1214
O	3.32966	0.58199	-2.5401	H	6.00794	-0.2024	1.34838
C	5.02739	-0.458	-0.5658	H	5.09644	-1.6972	1.20805
C	5.76549	-1.0216	0.65444	H	6.8129	-2.5905	-0.4003
C	7.05411	-1.7709	0.29148	H	7.7219	-1.095	-0.2613
C	7.79086	-2.3322	1.5118	H	8.70523	-2.8612	1.21959
O	-0.506	3.17978	-1.0508	H	8.07663	-1.5317	2.2053
O	-2.2883	4.29734	-0.1857	H	7.16	-3.0383	2.06587
C	-1.6228	3.96125	-1.1342	H	-2.7428	5.00221	-2.6234
C	-1.8909	4.32324	-2.5762	H	-1.0093	4.79327	-3.023
C	0.22712	-2.5264	0.2729	H	-2.1085	3.41886	-3.1546
O	1.04738	-1.7345	0.6893	H	1.32659	-3.3103	-2.6754
O	0.29627	-3.1023	-0.9457	H	1.38469	-1.6538	-1.9943
C	1.41775	-2.7208	-1.7629	H	2.35742	-2.9478	-1.2531
O	-4.2685	-2.3042	-0.6842	H	-7.3841	-3.1961	-0.1332
O	-5.3132	-2.6474	1.29981	H	-6.8134	-2.0202	-1.3536
C	-5.3396	-2.6166	0.0893	H	-6.3222	-3.7102	-1.48
C	-6.5473	-2.9078	-0.7701				

Conformer 7							
Atomic	Standard Orientation (Å)			Atomic	Standard Orientation (Å)		
Type	X	Y	Z	Type	X	Y	Z
C	-1.024	3.86668	-0.6633	H	-1.8251	4.456	-0.2284
C	-0.3943	2.72058	0.12646	H	-0.8146	4.692	-2.6612
C	0.69598	2.23642	-0.8136	H	-2.0209	1.90792	1.37912
C	0.5895	3.00937	-2.0895	H	2.27562	1.07831	-1.4684
C	-0.5146	3.98654	-1.8952	H	1.171	0.77449	1.43702
O	1.26781	2.84967	-3.0905	H	-1.2813	0.37912	-1.1807
C	-1.4618	1.70034	0.46821	H	2.5659	-1.1307	1.66775
C	1.60799	1.26266	-0.6273	H	3.79455	-0.5153	-1.0586
C	1.762	0.47057	0.5771	H	1.88926	-1.8415	-1.7933
C	-1.7993	0.62951	-0.2587	H	3.38408	-2.7722	-1.8637
C	2.55044	-0.614	0.71036	H	1.82481	-4.3127	-0.9744
C	3.39014	-1.2525	-0.3606	H	2.6272	-3.7495	0.49036
C	2.65306	-2.3308	-1.1792	H	-3.5371	-0.7484	-1.8203
C	1.98787	-3.4335	-0.3378	H	-2.5538	-1.9546	-1.0219
C	-2.9201	-0.2627	0.1708	H	-4.775	-2.8994	-1.1368
C	-3.3998	-1.2703	-0.861	H	-4.5287	-2.4416	0.54734
O	-3.3957	-0.1925	1.29669	H	-5.8337	-0.3386	0.17312
C	-4.6635	-2.0383	-0.4641	H	-6.0742	-0.7794	-1.5163
C	-5.9447	-1.1943	-0.5048	H	-7.3065	-2.8503	-0.7912
C	-7.2013	-1.9852	-0.1204	H	-7.0718	-2.3976	0.89026
C	-8.4812	-1.1444	-0.1676	H	-9.3602	-1.7354	0.11444
O	0.07756	3.14906	1.44495	H	-8.419	-0.291	0.51878
O	1.54533	4.66608	0.59354	H	-8.6572	-0.746	-1.1746
C	1.03725	4.11369	1.5389	H	2.07196	5.20011	3.0583
C	1.36701	4.37085	2.9916	H	0.45826	4.60114	3.55621
C	0.61668	-3.0357	0.18002	H	1.81112	3.47419	3.43726
O	-0.2464	-2.513	-0.4968	H	-0.785	-3.3998	3.07766
O	0.45217	-3.3669	1.47539	H	-1.0618	-2.0089	1.97792
C	-0.8423	-3.0773	2.03796	H	-1.6211	-3.6322	1.5084
O	4.51334	-1.8565	0.34437	H	7.67385	-2.6772	-0.0389
O	5.82808	-1.6121	-1.4876	H	6.93132	-1.9341	1.40841
C	5.68211	-1.9643	-0.3383	H	6.43033	-3.5498	0.90751
C	6.75353	-2.5719	0.53624				

Conformer 8							
Atomic	Standard Orientation (Å)			Atomic	Standard Orientation (Å)		
Type	X	Y	Z	Type	X	Y	Z
C	-0.3053	-4.3534	-0.6215	H	0.27783	-5.1761	-0.2198
C	-0.3485	-3.0115	0.10852	H	-1.0744	-5.1435	-2.493
C	-1.3242	-2.2134	-0.7361	H	1.70756	-2.9932	0.89791
C	-1.6749	-3.0315	-1.9398	H	-2.5213	-0.636	-1.3068
C	-0.9872	-4.3382	-1.7732	H	-0.9702	-0.5435	1.40308
O	-2.3807	-2.68	-2.8705	H	1.01874	-0.9399	-1.2779
C	1.06528	-2.4752	0.18677	H	-1.7303	1.7003	1.63078
C	-1.8383	-0.9849	-0.5327	H	-3.4906	1.30476	-0.8284
C	-1.5745	-0.1241	0.603	H	-1.412	1.96466	-1.934
C	1.6039	-1.4999	-0.5537	H	-2.6096	3.25852	-1.946
C	-2.0016	1.14768	0.7338	H	-0.5626	4.35839	-1.4617
C	-2.7903	1.93594	-0.2758	H	-1.3149	4.23395	0.13043
C	-1.9265	2.69636	-1.3016	H	3.38242	-0.5558	-2.4208
C	-0.8871	3.65058	-0.6886	H	2.97614	0.75585	-1.3455
C	3.0567	-1.1604	-0.4178	H	5.64308	-0.7767	-1.1856
C	3.59488	-0.1507	-1.4187	H	5.43323	0.65776	-2.1875
O	3.75859	-1.6863	0.43493	H	4.87074	2.00656	-0.1567
C	5.08644	0.16502	-1.2693	H	5.06148	0.57008	0.85152
C	5.41929	1.05601	-0.0647	H	7.46451	0.40419	0.16542
C	6.91796	1.35289	0.07083	H	7.28151	1.82336	-0.8542
C	7.24945	2.25307	1.26545	H	8.32645	2.44302	1.33844
O	-0.7224	-3.1505	1.51749	H	6.74593	3.22461	1.18243
O	-2.7277	-4.1156	1.0406	H	6.92787	1.79328	2.20814
C	-1.9237	-3.7087	1.84372	H	-3.0093	-4.2589	3.59835
C	-2.0918	-3.7264	3.34591	H	-1.2319	-4.2066	3.82284
C	0.36635	2.93378	-0.2207	H	-2.1468	-2.7004	3.72594
O	0.99844	2.14427	-0.8936	H	2.02139	3.0738	2.56076
O	0.71575	3.29765	1.03002	H	1.79728	1.59349	1.56962
C	1.90709	2.68086	1.55034	H	2.77386	2.94105	0.93777
O	-3.5735	2.88433	0.50475	H	-6.402	4.55997	0.46552
O	-5.1814	2.91177	-1.0947	H	-5.6782	3.72278	1.87019
C	-4.7614	3.28068	-0.0207	H	-4.8315	5.08875	1.14227
C	-5.469	4.22667	0.92072				

Conformer 9							
Atomic	Standard Orientation (Å)			Atomic	Standard Orientation (Å)		
Type	X	Y	Z	Type	X	Y	Z
C	1.73245	2.75292	1.81792	H	2.49752	3.51407	1.73158
C	0.8893	2.37411	0.60749	H	1.89637	2.08498	3.87841
C	-0.0869	1.355	1.18935	H	1.20033	1.7778	-1.5024
C	0.3246	1.07936	2.59674	H	-1.6876	0.05657	1.26627
C	1.42825	2.02757	2.90215	H	-1.2011	1.77912	-1.2874
O	-0.1456	0.23599	3.34581	H	3.4059	1.10365	0.51476
C	1.68342	1.76841	-0.5267	H	-2.8745	0.4866	-2.3573
C	-1.1566	0.75747	0.62341	H	-3.3161	-0.9826	0.30605
C	-1.6489	0.96517	-0.7242	H	-3.5029	-3.0777	-0.9903
C	2.86659	1.15088	-0.428	H	-2.8944	-2.2725	-2.4407
C	-2.6045	0.23318	-1.3324	H	-1.0638	-3.5505	-1.4517
C	-3.3377	-0.9584	-0.7831	H	-0.6822	-1.8564	-1.2477
C	-2.825	-2.2955	-1.3467	H	5.19491	-0.4469	-2.4311
C	-1.3882	-2.6355	-0.9396	H	5.5382	0.6124	-1.0493
C	3.47881	0.50428	-1.6268	H	5.85963	-1.7359	-0.4097
C	4.8426	-0.1446	-1.4399	H	4.55887	-1.0311	0.5315
O	2.90808	0.49237	-2.7085	H	2.85549	-2.139	-0.9112
C	4.83142	-1.3565	-0.4823	H	4.11624	-2.7627	-1.9633
C	3.89642	-2.4916	-0.9206	H	5.04371	-4.1177	-0.0761
C	4.01232	-3.739	-0.035	H	3.83535	-3.4566	1.01331
C	3.03716	-4.8517	-0.4332	H	3.16315	-5.738	0.19947
O	0.09836	3.50394	0.11624	H	1.99903	-4.5135	-0.3357
O	1.96	4.6992	-0.3996	H	3.19433	-5.1615	-1.4738
C	0.75492	4.59134	-0.3719	H	0.30731	6.50976	-1.1954
C	-0.2328	5.623	-0.8626	H	-0.8205	5.21473	-1.6916
C	-1.2318	-2.8655	0.55392	H	-0.934	5.88508	-0.0641
O	-2.1461	-2.9445	1.34829	H	1.39811	-3.2796	2.39348
O	0.06663	-2.9734	0.89801	H	0.00297	-2.2449	2.85259
C	0.32015	-3.1388	2.31056	H	-0.2132	-4.0119	2.69384
O	-4.7281	-0.8796	-1.2045	H	-7.598	0.50341	-0.3911
O	-5.1886	0.51348	0.52671	H	-6.9753	0.1648	-2.0353
C	-5.5509	-0.1076	-0.4458	H	-7.3447	-1.176	-0.9497
C	-6.9612	-0.1509	-0.9872				

Conformer 10							
Atomic	Standard Orientation (Å)			Atomic	Standard Orientation (Å)		
Type	X	Y	Z	Type	X	Y	Z
C	0.26533	3.68237	1.44494	H	0.97792	4.49444	1.34162
C	0.01246	2.73325	0.27645	H	-0.3706	3.81538	3.51851
C	-1.0963	1.83943	0.81867	H	1.49835	1.24724	0.86197
C	-1.2927	2.17566	2.26096	H	-2.4397	0.2778	0.85663
C	-0.4134	3.33784	2.54668	H	-1.0827	1.08205	-1.8289
O	-2.0126	1.58342	3.05187	H	1.78767	2.59134	-1.8989
C	1.26428	1.91423	0.03644	H	-1.7619	-1.0279	-2.6986
C	-1.7354	0.81528	0.22304	H	-2.8419	-1.6623	0.13155
C	-1.5691	0.3827	-1.1538	H	-1.9449	-3.8742	-0.0418
C	2.03045	1.94353	-1.0619	H	-1.3356	-3.5872	-1.6768
C	-1.9367	-0.8182	-1.644	H	0.5118	-3.6349	-0.0586
C	-2.4882	-1.9674	-0.8536	H	0.42412	-2.0618	-0.8321
C	-1.4813	-3.1266	-0.692	H	3.98328	0.82955	0.70715
C	-0.1022	-2.7276	-0.1431	H	2.89809	-0.4374	0.19468
C	3.23672	1.0959	-1.2775	H	4.65656	-1.2823	-1.4159
C	3.7294	0.19185	-0.1537	H	5.74469	-0.0167	-0.8966
O	3.8079	1.14997	-2.3587	H	5.66016	-0.9112	1.46571
C	4.93194	-0.6678	-0.5508	H	4.61151	-2.214	0.93193
C	5.42593	-1.5512	0.60261	H	7.47765	-1.7356	-0.06
C	6.66472	-2.399	0.2682	H	7.0188	-2.8801	1.19003
C	6.4277	-3.4783	-0.795	H	7.32956	-4.0814	-0.9514
O	-0.2825	3.42545	-0.9602	H	5.62164	-4.159	-0.4922
O	-2.1168	4.45514	-0.0965	H	6.1507	-3.0456	-1.7627
C	-1.366	4.25465	-1.0197	H	-2.3076	5.56672	-2.4152
C	-1.476	4.86171	-2.3985	H	-0.545	5.37103	-2.6666
C	-0.0617	-2.0531	1.21917	H	-1.6443	4.07556	-3.1423
O	0.80025	-1.2671	1.56375	H	-1.8934	-2.4284	3.8616
O	-1.0661	-2.4553	2.01449	H	-0.1421	-2.0243	3.83964
C	-1.1044	-1.8854	3.34169	H	-1.3448	-0.8207	3.28988
O	-3.63	-2.4658	-1.6078	H	-6.5671	-3.8935	-1.2341
O	-4.6588	-3.1095	0.31006	H	-6.1513	-2.5809	-2.376
C	-4.6533	-3.005	-0.896	H	-5.3876	-4.1614	-2.5534
C	-5.7659	-3.442	-1.82				

Table S5. Experimental and calculated ^{1}H NMR data for compound **1**.

No.	**1**, exptl. δ_H [a]	4*R*,12*S*-**1**, calcd. δ_H [b]	4*S*,12*S*-**1**, calcd. δ_H [b]
2	2.38	2.59	2.41
	1.29	2.44	2.28
3	2.05	2.45	2.29
	1.95	1.87	2.15
4	5.75	5.59	5.74
5	5.84	6.16	6.45
6	6.35	6.74	7.09
7	7.39	7.92	7.75
10	6.55	6.82	6.93
11	7.48	7.62	8.38
13	6.77	7.36	7.53
14	6.31	6.54	6.59
16	2.53	2.55	2.62
17	1.59	1.62	1.58
18	1.29	1.02	1.15
19	1.29	1.23	1.34
20	0.88	0.92	0.79
1-OMe	3.7	3.87	3.70
2′	2.04	2.20	2.14
2″	2.09	2.09	2.11

[a] Recorded in $CDCl_3$ at 600 MHz.

[b] Calculated in $CDCl_3$

Table S6. Experimental and calculated ^{13}C NMR data for compound **1**.

No.	**1**, exptl. δ_C [a]	4*R*,12*S*-**1**, calcd. δ_C [b]	4*S*,12*S*-**1**, calcd. δ_C [b]
1	172.9	178.2	179.6
2	29.7	28.2	27.1
3	29.7	26.8	25.5
4	69.3	68.1	66.1
5	140	144.1	144.9
6	124.4	126.2	126.5
7	126.6	129.2	127.7
8	135.7	132.4	131.6
9	192.7	192.9	191.4
10	136.4	139.8	140.3
11	155	156.2	157.3
12	83.5	80.7	79.5
13	141	144.7	143.5
14	129.2	131.0	130.5
15	199.6	204.8	202.6
16	41.2	39.7	42.3
17	23.5	26.4	23.4
18	31.3	30.3	30.3
19	22.4	21.9	22.2
20	13.9	11.0	10.9
1-OMe	51.9	48.4	49.3
1′	170	175.4	174.5
2′	21.3	18.0	17.8
1″	168.8	172.5	174.4
2″	21.2	18.3	18.4

[a] Recorded in $CDCl_3$ at 600 MHz.

[b] Calculated in $CDCl_3$

Table S7. DP4+ analyses of calculated and experimental NMR chemical shifts of **1** (unscaled). Isomer 1: 4*R*,12*S*-**1**; Isomer 2: 4*S*,12*S*-**1**

Functional	Solvent?	Basis Set	Type of Data			
mPW1PW91	PCM	6-311+G(d,p)	Unscaled Shifts			
	Isomer 1	Isomer 2	Isomer 3	Isomer 4	Isomer 5	Isomer 6
sDP4+ (H data)	84.32%	15.68%	-	-	-	-
sDP4+ (C data)	99.51%	0.49%	-	-	-	-
sDP4+ (all data)	99.91%	0.09%	-	-	-	-
uDP4+ (H data)	96.66%	3.34%	-	-	-	-
uDP4+ (C data)	99.93%	0.07%	-	-	-	-
uDP4+ (all data)	100.00%	0.00%	-	-	-	-
DP4+ (H data)	99.36%	0.64%	-	-	-	-
DP4+ (C data)	100.00%	0.00%	-	-	-	-
DP4+ (all data)	100.00%	0.00%	-	-	-	-

Functional		Solvent?		Basis Set		Type of Data
mPW1PW91		PCM		6-311+G(d,p)		Unscaled Shifts
		DP4+	100.00%	0.00%	-	-	-
Nuclei	sp2?	Experimenta	Isomer 1	Isomer 2	Isomer 3	Isomer 4	Isomer 5
C	x	172.9	178.2	179.6			
C		29.7	28.2	27.1			
C		29.7	26.8	25.5			
C		69.3	68.1	66.1			
C	x	140.0	144.1	144.9			
C	x	124.4	126.2	126.5			
C	x	126.6	129.2	127.7			
C	x	135.7	132.4	131.6			
C	x	192.7	192.9	191.4			
C	x	136.4	139.8	140.3			
C	x	155.0	156.2	157.3			
C		83.5	80.65	79.53			
C	x	141.0	144.66	143.50			
C	x	129.2	130.97	130.53			
C	x	199.6	204.78	202.58			
C		41.2	39.67	42.29			
C		23.5	26.36	23.41			
C		31.3	30.32	30.25			
C		22.4	21.89	22.18			
C		13.9	10.98	10.91			
C		51.9	48.37	49.26			
C	x	170.0	175.41	174.47			
C		21.3	18.04	17.76			
C	x	168.8	172.50	174.36			
C		21.2	18.30	18.42			
H		2.38	2.58518849	2.41448727			
H		1.29	2.43990968	2.27709942			
H		2.05	2.44954676	2.29200722			
H		1.95	1.87121344	2.1475089			
H		5.75	5.59041485	5.73881978			
H	x	5.84	6.16074762	6.4481194			
H	x	6.35	6.73651802	7.08593171			
H	x	7.39	7.91950138	7.75119996			
H	x	6.55	6.81510378	6.93090113			
H	x	7.48	7.61945923	8.37815606			
H	x	6.77	7.35598783	7.53311107			
H	x	6.31	6.54216996	6.58805942			
H		2.53	2.55168596	2.61700834			
H		1.59	1.61731089	1.57946925			
H		1.29	1.01922143	1.14774555			
H		1.29	1.22741903	1.3361358			
H		0.88	0.91671997	0.79032632			
H		3.70	3.87041314	3.70000147			
H		2.04	2.19913453	2.14469985			
H		2.09	2.08721569	2.10528836			

Table S8. Energy analyses of 7*S*,8*R*,12*R*-**2**/7*R*,8*S*,12*S*-**2** (eleven conformers)

**NO.**	**3D conformers** B3LYP/6-31G(d,p)	**G (Hartree)**	**Boltzmann distribution**	**Calculated ECD spectrum** 7*S*,8*R*,12*R*-**2**
1	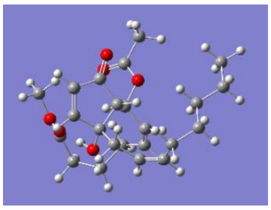	-1347.768036	3.55 %	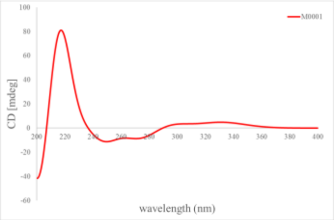
2	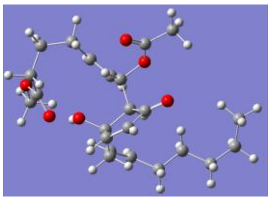	-1347.766188	0.5 %	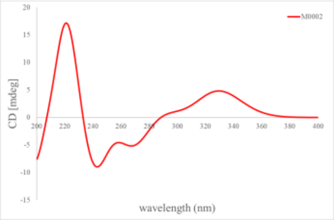
3	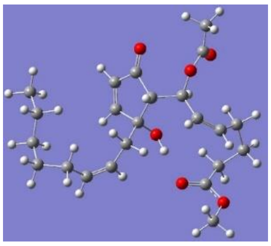	-1347.766520	0.71 %	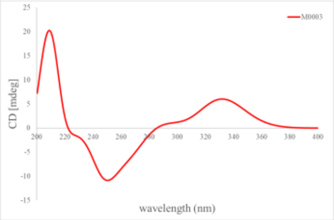
4	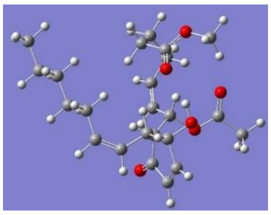	-1347.770379	42.49 %	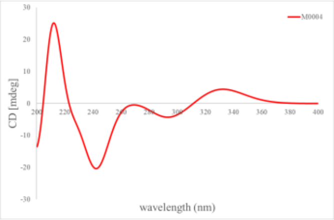
5	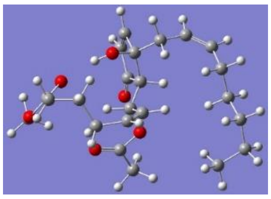	-1347.766810	0.97 %	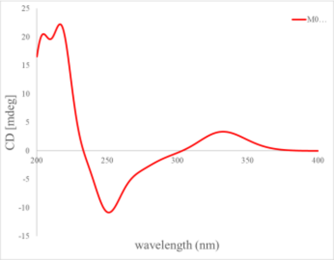

6	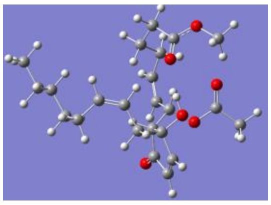	-1347.768948	9.33 %	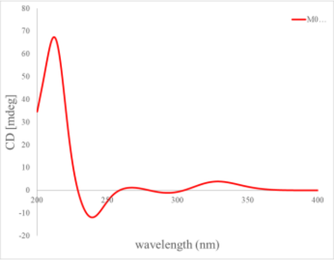
7	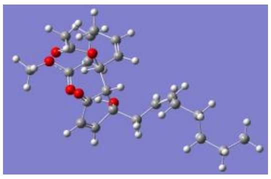	-1347.768453	5.53 %	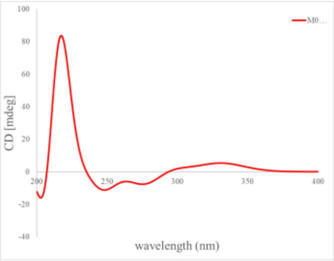
8	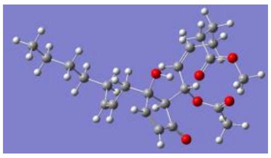	-1347.770035	29.51 %	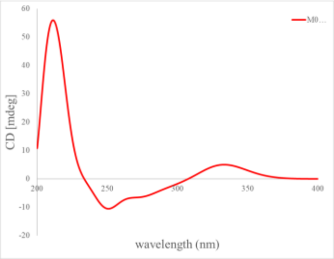
9	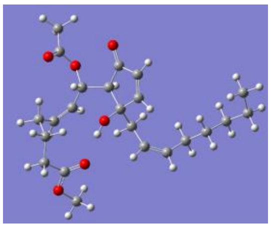	-1347.767498	2.01 %	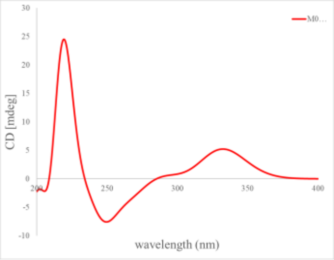
10	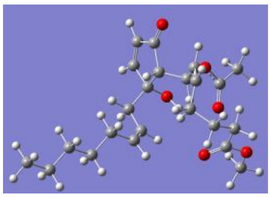	-1347.767665	2.4 %	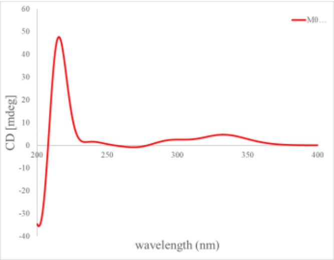
11	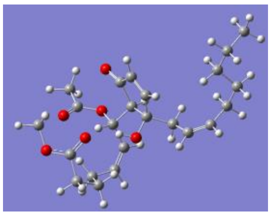	-1347.767761	2.66 %	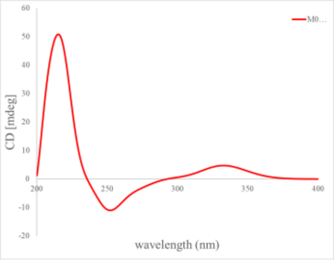

Table S9. Cartesian coordinates of the low-energy re-optimized conformers of 7*S*,8*R*,12*R*-**2**/7*R*,8*S*,12*S*-**2** calculated at B3LYP/6-31G(d,p) level of theory.

Conformer 1							
Atomic	Standard Orientation (Å)			Atomic	Standard Orientation (Å)		
Type	X	Y	Z	Type	X	Y	Z
C	-0.288	-0.0857	3.1934	H	1.17168	-3.3259	1.67923
C	-0.292	-1.3387	2.72446	H	2.21528	-1.9234	1.91488
C	0.194	-1.4659	1.29198	H	1.26167	-2.8627	-0.8733
C	0.48856	0.02354	0.87213	H	3.50041	-2.7117	-1.5765
C	0.21157	0.84465	2.15904	H	4.23048	-1.8664	1.31276
O	0.40106	2.03829	2.31081	H	5.11803	-3.0666	0.38979
C	1.46113	-2.3557	1.24909	H	6.32672	-0.9914	0.23349
C	2.0085	-2.5769	-0.136	H	5.66647	-1.3523	-1.3544
C	3.2869	-2.4894	-0.5284	H	3.78623	0.27084	-0.936
C	4.50677	-2.156	0.29167	H	4.4479	0.62519	0.65312
C	5.38712	-1.0537	-0.333	H	6.55978	1.47502	-0.4191
C	4.7266	0.32951	-0.3699	H	5.89409	1.12395	-2.006
C	5.6176	1.41739	-0.983	H	5.60974	3.55067	-1.4572
C	4.95197	2.79717	-1.0088	H	4.02138	2.77727	-1.5891
O	-0.7858	-2.1463	0.51651	H	4.69957	3.13415	0.00396
C	-0.326	0.53267	-0.341	H	-1.6677	-1.7438	0.65629
C	0.06107	-0.0963	-1.6547	H	-1.3896	0.42627	-0.1407
C	-0.7889	-0.4649	-2.6204	H	1.13061	-0.2168	-1.8158
C	-2.2939	-0.3911	-2.5582	H	-0.3668	-0.8952	-3.5293
O	-0.044	1.96006	-0.4699	H	-2.5956	0.38571	-1.8473
C	-2.9391	-1.7441	-2.1611	H	-2.6885	-0.0737	-3.5326
C	-4.328	-1.5701	-1.5244	H	-2.2863	-2.2604	-1.4528
C	-4.24	-0.8062	-0.2156	H	-3.0278	-2.394	-3.0397
C	-1.0501	2.8264	-0.2331	H	-4.7606	-2.5545	-1.3003
O	-2.2065	2.50414	-0.0371	H	-5.0225	-1.0526	-2.1929
C	-0.5342	4.2431	-0.2269	H	0.05412	4.38382	0.68621
O	-5.1907	0.12426	-0.1028	H	0.12633	4.41977	-1.0806
O	-3.3997	-1.0029	0.65147	H	-1.3714	4.94223	-0.2396
C	-5.126	0.95995	1.07612	H	-5.157	0.34567	1.9789
H	-0.5832	0.25353	4.1803	H	-4.2075	1.54995	1.05312
H	-0.6123	-2.2266	3.26354	H	-6.0038	1.60318	1.01601
H	1.54996	0.15253	0.62824				

Conformer 2							
Atomic	Standard Orientation (Å)			Atomic	Standard Orientation (Å)		
Type	X	Y	Z	Type	X	Y	Z
C	0.91394	1.91254	3.04189	H	-0.0374	-1.8135	3.08879
C	0.08211	0.88212	3.22862	H	1.53209	-1.2351	2.53574
C	-0.1366	0.03064	1.99116	H	-0.6077	-2.5774	0.73739
C	0.61839	0.83093	0.86821	H	1.1578	-3.5754	-0.4141
C	1.37834	1.93916	1.6391	H	3.10667	-2.0713	1.46645
O	2.23013	2.67769	1.17891	H	3.30965	-3.7301	0.92645
C	0.49083	-1.3759	2.22908	H	3.43349	-2.9442	-1.4601
C	0.40067	-2.3211	1.05869	H	3.25929	-1.283	-0.9136
C	1.41766	-2.8982	0.40231	H	5.38925	-1.4856	0.40351
C	2.89828	-2.7515	0.63252	H	5.52784	-3.1723	-0.0714
C	3.65178	-2.2657	-0.6218	H	7.03964	-1.8877	-1.4408
C	5.17038	-2.19	-0.4129	H	5.75426	-2.4863	-2.4756
C	5.96907	-1.7792	-1.6612	H	6.34234	-0.0971	-2.9983
C	5.70398	-0.3478	-2.1433	H	4.66408	-0.2065	-2.4579
O	-1.5462	-0.0453	1.82731	H	5.91	0.38066	-1.3492
C	-0.3211	1.41554	-0.2122	H	-1.812	-0.8377	1.32024
C	-0.8689	0.3771	-1.1584	H	-1.1117	1.99248	0.26247
C	-2.0882	0.37444	-1.7112	H	-0.1618	-0.3974	-1.4526
C	-3.2115	1.3489	-1.4753	H	-2.3092	-0.4173	-2.4308
O	0.47102	2.32152	-1.0338	H	-2.9425	2.07352	-0.7006
C	-4.5549	0.68031	-1.1173	H	-3.3683	1.93361	-2.3945
C	-4.5051	-0.2172	0.13943	H	-4.9249	0.09042	-1.9641
C	-3.9259	-1.5968	-0.0946	H	-5.2939	1.47106	-0.9435
C	0.20546	3.64679	-0.941	H	-5.5286	-0.3708	0.5053
O	-0.7114	4.11831	-0.3021	H	-3.9267	0.25494	0.93758
C	1.22414	4.43772	-1.7234	H	2.17994	4.38295	-1.1911
O	-4.6039	-2.2705	-1.041	H	1.37091	4.00669	-2.718
O	-2.9837	-2.0909	0.50405	H	0.90307	5.47732	-1.8007
C	-4.1422	-3.6043	-1.3204	H	-4.8053	-3.984	-2.0978
H	1.2466	2.63655	3.77741	H	-3.1075	-3.585	-1.6721
H	-0.4199	0.61661	4.15483	H	-4.2034	-4.2258	-0.4236
H	1.36307	0.20891	0.35869				

Conformer 3							
Atomic	Standard Orientation (Å)			Atomic	Standard Orientation (Å)		
Type	X	Y	Z	Type	X	Y	Z
C	-1.7484	2.08618	-1.1377	H	-1.9614	-0.3392	1.38627
C	-1.6968	0.75066	-1.1901	H	-0.4665	-1.2654	1.34144
C	-0.6533	0.13703	-0.2744	H	-1.3936	-2.5463	-0.7111
C	-0.0486	1.38182	0.47279	H	-3.5423	-3.3692	-0.271
C	-0.7605	2.6032	-0.1681	H	-4.2557	-2.7215	2.14698
O	-0.573	3.77597	0.09948	H	-3.9075	-1.0516	1.74685
C	-1.285	-0.8778	0.71455	H	-5.9494	-2.8722	0.35106
C	-1.9842	-2.0328	0.04564	H	-6.3389	-1.655	1.556
C	-3.2135	-2.4992	0.30186	H	-5.1192	-1.0652	-1.1995
C	-4.2197	-2.0066	1.31008	H	-6.8288	-0.9589	-0.8088
C	-5.647	-1.8872	0.73429	H	-6.3037	0.81358	0.91745
C	-5.8216	-0.8456	-0.3826	H	-4.6158	0.76725	0.42874
C	-5.6396	0.61159	0.06439	H	-5.7895	2.64939	-0.712
C	-5.9277	1.6177	-1.0548	H	-5.2602	1.45905	-1.911
O	0.28042	-0.5065	-1.139	H	-6.9581	1.52188	-1.4193
C	1.48888	1.47008	0.42717	H	0.65356	-1.299	-0.6998
C	2.17925	0.38258	1.21249	H	1.82724	1.49615	-0.606
C	3.33832	-0.2112	0.9006	H	1.70625	0.12631	2.16098
C	4.19611	0.01805	-0.3155	H	3.73446	-0.9387	1.61286
O	1.85689	2.72888	1.06103	H	3.72242	0.7277	-1.0001
C	4.57328	-1.2717	-1.0724	H	5.13341	0.49538	0.00827
C	3.3661	-2.0843	-1.5918	H	5.19417	-1.918	-0.4409
C	2.68008	-2.9323	-0.5412	H	5.19117	-0.9925	-1.9336
C	2.51405	3.64166	0.30653	H	3.71322	-2.7758	-2.3701
O	2.93057	3.43355	-0.8135	H	2.61348	-1.4273	-2.0358
C	2.62735	4.9475	1.05259	H	1.63013	5.39811	1.09975
O	3.52442	-3.8163	0.0173	H	2.96729	4.78107	2.07897
O	1.50068	-2.8758	-0.2291	H	3.31147	5.61493	0.52694
C	2.96011	-4.6829	1.01867	H	2.1596	-5.2909	0.59
H	-2.395	2.74499	-1.7068	H	3.78396	-5.3124	1.3549
H	-2.2936	0.10802	-1.8293	H	2.5579	-4.0966	1.84861
H	-0.3462	1.37449	1.53058				

Conformer 4							
Atomic	Standard Orientation (Å)			Atomic	Standard Orientation (Å)		
Type	X	Y	Z	Type	X	Y	Z
C	2.04008	-3.4825	-0.4917	H	-0.493	-1.1765	2.13009
C	2.07755	-3.033	0.76917	H	0.44596	-2.4537	2.89401
C	1.22764	-1.8021	1.0052	H	-0.5188	-4.1549	1.26879
C	0.7731	-1.3995	-0.4395	H	-2.7954	-3.8886	0.81942
C	1.20493	-2.5903	-1.325	H	-3.8499	-1.9315	2.08229
O	0.89989	-2.7584	-2.4919	H	-2.4925	-0.9125	1.62726
C	0.01711	-2.1251	1.9363	H	-3.0803	-1.225	-0.8004
C	-0.9447	-3.1645	1.42909	H	-4.4099	-2.2777	-0.3473
C	-2.2507	-3.01	1.17067	H	-5.4834	-0.3825	0.9182
C	-3.0932	-1.7684	1.29839	H	-4.1581	0.67493	0.45805
C	-3.8228	-1.4106	-0.0113	H	-4.7444	0.35201	-1.9682
C	-4.7461	-0.1935	0.12357	H	-6.0697	-0.7027	-1.5069
C	-5.4813	0.16421	-1.1745	H	-6.9114	1.60968	-1.9754
C	-6.4015	1.38102	-1.0327	H	-7.1717	1.20927	-0.2706
O	2.05463	-0.8463	1.65905	H	-5.8368	2.27342	-0.7349
C	1.35495	-0.067	-0.9484	H	1.49253	-0.0897	1.91375
C	0.65562	0.41128	-2.1964	H	1.29105	0.67624	-0.1613
C	0.2908	1.67614	-2.4477	H	0.41673	-0.3654	-2.9189
C	0.57712	2.91473	-1.6338	H	-0.2575	1.85514	-3.3747
O	2.7775	-0.2855	-1.2078	H	1.39304	2.73728	-0.9267
C	-0.6414	3.50787	-0.8905	H	0.94342	3.68531	-2.3265
C	-1.137	2.66032	0.29574	H	-1.4786	3.63823	-1.5883
C	-0.0903	2.40704	1.36419	H	-0.3751	4.50507	-0.5245
C	3.65544	0.58033	-0.6643	H	-1.4923	1.68059	-0.0352
O	3.35471	1.59926	-0.0727	H	-1.9842	3.16879	0.77611
C	5.07152	0.10193	-0.8794	H	5.25966	-0.7525	-0.2196
O	0.60847	3.50561	1.66963	H	5.21398	-0.2368	-1.9094
O	0.10007	1.32318	1.89672	H	5.77071	0.90423	-0.6402
C	1.70688	3.33003	2.59129	H	2.4701	2.69854	2.13058
H	2.52615	-4.3655	-0.8921	H	2.09388	4.33329	2.7687
H	2.62288	-3.4828	1.59491	H	1.35504	2.88065	3.52265
H	-0.3173	-1.3172	-0.4987				

Conformer 5							
Atomic	Standard Orientation (Å)			Atomic	Standard Orientation (Å)		
Type	X	Y	Z	Type	X	Y	Z
C	-0.5913	2.46769	-2.4332	H	1.48808	2.10501	1.45098
C	-0.3889	3.12192	-1.283	H	0.95532	3.73345	1.06818
C	0.05409	2.22433	-0.1384	H	2.36535	3.74417	-1.0383
C	0.32222	0.85302	-0.8462	H	4.46599	2.74463	-0.846
C	-0.2719	1.0315	-2.2665	H	5.07592	1.76636	1.36445
O	-0.4369	0.1656	-3.1067	H	3.41809	1.27019	1.6642
C	1.28151	2.78437	0.61936	H	3.53657	-0.3251	-0.2595
C	2.4979	3.01751	-0.2357	H	5.17562	0.19029	-0.6138
C	3.70483	2.446	-0.1228	H	5.96724	-0.6252	1.58979
C	4.16025	1.40875	0.86939	H	4.3349	-1.0823	2.05104
C	4.45749	0.04761	0.20681	H	5.83436	-2.2916	-0.3374
C	5.0126	-0.9939	1.18817	H	5.82089	-2.9934	1.27131
C	5.22652	-2.3881	0.57376	H	4.14285	-4.1258	-0.1776
C	3.92858	-3.1398	0.25093	H	3.33047	-3.295	1.15762
O	-0.9849	2.202	0.83897	H	3.29989	-2.5978	-0.4644
C	-0.1778	-0.411	-0.1236	H	-1.8184	1.86411	0.45068
C	0.39068	-0.6243	1.25453	H	-1.2644	-0.4238	-0.1081
C	-0.3086	-0.9936	2.33502	H	1.4693	-0.4992	1.34097
C	-1.7997	-1.2184	2.39476	H	0.23633	-1.1199	3.27118
O	0.2645	-1.5351	-0.9501	H	-2.1599	-1.5314	1.40891
C	-2.5774	0.03824	2.86663	H	-2.0164	-2.0558	3.07096
C	-4.0311	0.05808	2.36187	H	-2.0647	0.93381	2.50796
C	-4.0883	0.13315	0.8465	H	-2.5816	0.09205	3.9618
C	-0.6833	-2.3551	-1.4505	H	-4.5451	0.94627	2.75307
O	-1.8594	-2.3187	-1.1451	H	-4.5915	-0.8202	2.69586
C	-0.0824	-3.306	-2.4547	H	0.12893	-2.7399	-3.3685
O	-4.9455	-0.7484	0.32546	H	0.86191	-3.7179	-2.088
O	-3.43	0.90207	0.15925	H	-0.7895	-4.107	-2.6748
C	-4.9952	-0.8117	-1.1185	H	-5.7957	-1.5162	-1.3442
H	-0.964	2.87054	-3.3687	H	-5.2165	0.17358	-1.535
H	-0.5742	4.17821	-1.1048	H	-4.0385	-1.1756	-1.4987
H	1.40576	0.74762	-0.9928				

Conformer 6							
Atomic	Standard Orientation (Å)			Atomic	Standard Orientation (Å)		
Type	X	Y	Z	Type	X	Y	Z
C	1.06973	-3.8961	0.1948	H	-0.0623	-1.9381	3.27401
C	1.3071	-3.1948	1.31043	H	-1.1567	-2.5524	2.03481
C	0.73363	-1.7902	1.28487	H	-0.5888	0.45568	2.5299
C	0.26858	-1.6291	-0.2036	H	-2.851	0.83241	2.14908
C	0.3653	-3.053	-0.7951	H	-3.192	-2.2263	1.82978
O	-0.0687	-3.4028	-1.8776	H	-4.3796	-1.1203	2.49831
C	-0.473	-1.7317	2.27506	H	-3.4289	-1.136	-0.418
C	-1.2005	-0.4136	2.29648	H	-4.9148	-1.8581	0.18301
C	-2.506	-0.1993	2.07312	H	-5.7043	0.4251	0.9283
C	-3.5795	-1.2051	1.74654	H	-4.251	1.13789	0.24103
C	-4.2113	-1.0295	0.34644	H	-4.8882	0.25843	-2.0248
C	-4.9515	0.29783	0.13522	H	-6.3461	-0.4419	-1.3416
C	-5.639	0.39279	-1.2334	H	-6.8568	1.75417	-2.4331
C	-6.3769	1.71813	-1.4484	H	-7.1578	1.86493	-0.6919
O	1.77452	-0.9157	1.69632	H	-5.6893	2.57048	-1.382
C	1.09947	-0.6215	-1.0208	H	1.45493	0.00562	1.73085
C	0.44148	-0.2424	-2.3209	H	1.29076	0.26003	-0.4158
C	0.21029	1.02077	-2.7028	H	0.11714	-1.0728	-2.9436
C	0.61009	2.26056	-1.9346	H	-0.3143	1.17684	-3.6456
O	2.39419	-1.2531	-1.2773	H	1.58926	2.09057	-1.4732
C	-0.4073	2.68047	-0.8469	H	0.74524	3.09511	-2.6345
C	0.19634	3.66768	0.17083	H	-0.7571	1.79763	-0.3012
C	1.28322	2.99354	0.98718	H	-1.2915	3.13492	-1.3097
C	3.50347	-0.5125	-1.0983	H	-0.5819	3.99741	0.87073
O	3.51048	0.67372	-0.8313	H	0.60544	4.55509	-0.3206
C	4.74087	-1.3631	-1.2618	H	4.81624	-2.0527	-0.4137
O	2.44216	3.65601	0.9509	H	4.67549	-1.9659	-2.1722
O	1.12215	1.95124	1.60492	H	5.62427	-0.7241	-1.2916
C	3.55478	3.03233	1.63137	H	4.36832	3.75489	1.56718
H	1.32962	-4.9307	-0.0012	H	3.29739	2.83061	2.67372
H	1.82002	-3.5542	2.19859	H	3.81756	2.10414	1.1202
H	-0.7732	-1.2961	-0.257				

Conformer 7							
Atomic	Standard Orientation (Å)			Atomic	Standard Orientation (Å)		
Type	X	Y	Z	Type	X	Y	Z
C	1.06973	-3.8961	0.1948	H	-0.0623	-1.9381	3.27401
C	1.3071	-3.1948	1.31043	H	-1.1567	-2.5524	2.03481
C	0.73363	-1.7902	1.28487	H	-0.5888	0.45568	2.5299
C	0.26858	-1.6291	-0.2036	H	-2.851	0.83241	2.14908
C	0.3653	-3.053	-0.7951	H	-3.192	-2.2263	1.82978
O	-0.0687	-3.4028	-1.8776	H	-4.3796	-1.1203	2.49831
C	-0.473	-1.7317	2.27506	H	-3.4289	-1.136	-0.418
C	-1.2005	-0.4136	2.29648	H	-4.9148	-1.8581	0.18301
C	-2.506	-0.1993	2.07312	H	-5.7043	0.4251	0.9283
C	-3.5795	-1.2051	1.74654	H	-4.251	1.13789	0.24103
C	-4.2113	-1.0295	0.34644	H	-4.8882	0.25843	-2.0248
C	-4.9515	0.29783	0.13522	H	-6.3461	-0.4419	-1.3416
C	-5.639	0.39279	-1.2334	H	-6.8568	1.75417	-2.4331
C	-6.3769	1.71813	-1.4484	H	-7.1578	1.86493	-0.6919
O	1.77452	-0.9157	1.69632	H	-5.6893	2.57048	-1.382
C	1.09947	-0.6215	-1.0208	H	1.45493	0.00562	1.73085
C	0.44148	-0.2424	-2.3209	H	1.29076	0.26003	-0.4158
C	0.21029	1.02077	-2.7028	H	0.11714	-1.0728	-2.9436
C	0.61009	2.26056	-1.9346	H	-0.3143	1.17684	-3.6456
O	2.39419	-1.2531	-1.2773	H	1.58926	2.09057	-1.4732
C	-0.4073	2.68047	-0.8469	H	0.74524	3.09511	-2.6345
C	0.19634	3.66768	0.17083	H	-0.7571	1.79763	-0.3012
C	1.28322	2.99354	0.98718	H	-1.2915	3.13492	-1.3097
C	3.50347	-0.5125	-1.0983	H	-0.5819	3.99741	0.87073
O	3.51048	0.67372	-0.8313	H	0.60544	4.55509	-0.3206
C	4.74087	-1.3631	-1.2618	H	4.81624	-2.0527	-0.4137
O	2.44216	3.65601	0.9509	H	4.67549	-1.9659	-2.1722
O	1.12215	1.95124	1.60492	H	5.62427	-0.7241	-1.2916
C	3.55478	3.03233	1.63137	H	4.36832	3.75489	1.56718
H	1.32962	-4.9307	-0.0012	H	3.29739	2.83061	2.67372
H	1.82002	-3.5542	2.19859	H	3.81756	2.10414	1.1202
H	-0.7732	-1.2961	-0.257				

Conformer 8							
Atomic	Standard Orientation (Å)			Atomic	Standard Orientation (Å)		
Type	X	Y	Z	Type	X	Y	Z
C	0.47283	-2.0409	2.6413	H	-2.0581	0.68219	0.2691
C	-0.2511	-0.9201	2.74681	H	-2.3085	0.73197	2.00395
C	-0.4857	-0.21	1.42424	H	-2.8206	-1.7881	1.99091
C	0.14771	-1.1888	0.37223	H	-4.2755	-2.4162	0.27645
C	0.81928	-2.2946	1.22519	H	-3.3653	0.10363	-1.2779
O	1.49339	-3.2225	0.81555	H	-3.6946	-1.4672	-1.9977
C	-1.9869	0.07973	1.17898	H	-5.6177	-0.0676	-2.3825
C	-2.8708	-1.1361	1.11786	H	-6.083	-1.3272	-1.2489
C	-3.7022	-1.4981	0.13109	H	-5.7993	0.30107	0.65878
C	-3.981	-0.7975	-1.1729	H	-5.3534	1.55558	-0.4868
C	-5.4678	-0.4211	-1.353	H	-7.6067	1.34964	-1.59
C	-5.9639	0.64711	-0.3711	H	-8.0532	0.09797	-0.442
C	-7.4436	1.00553	-0.5585	H	-8.9928	2.31063	0.26268
C	-7.9334	2.07767	0.42032	H	-7.8157	1.74827	1.46005
O	0.11316	1.08164	1.48444	H	-7.3665	3.00959	0.30365
C	1.09986	-0.5549	-0.6608	H	1.06491	1.00403	1.71276
C	0.45402	0.51605	-1.5053	H	1.99593	-0.1835	-0.1694
C	1.0826	1.54616	-2.0842	H	-0.6163	0.40394	-1.6717
C	2.5528	1.87684	-2.0455	H	0.47271	2.23649	-2.6701
O	1.51124	-1.642	-1.5468	H	3.13569	1.04442	-1.6382
C	2.88702	3.17382	-1.2759	H	2.89728	2.00783	-3.0815
C	2.61511	3.08911	0.23749	H	2.29897	4.0078	-1.6807
C	3.39214	1.99264	0.93923	H	3.94181	3.41882	-1.4395
C	2.81805	-1.9802	-1.5652	H	1.55567	2.91124	0.43802
O	3.70565	-1.3419	-1.033	H	2.88996	4.0421	0.70935
C	3.01819	-3.2671	-2.3247	H	4.07668	-3.4033	-2.5506
O	4.68972	1.99243	0.6177	H	2.66846	-4.0874	-1.6885
O	2.90944	1.18316	1.72029	H	2.42417	-3.2762	-3.2427
C	5.47408	0.89941	1.14743	H	6.49279	1.09113	0.81057
H	0.80305	-2.6947	3.44114	H	5.42228	0.88826	2.23839
H	-0.6218	-0.4801	3.66931	H	5.10165	-0.0449	0.74447
H	-0.6568	-1.6843	-0.1874				

Conformer 9							
Atomic	Standard Orientation (Å)			Atomic	Standard Orientation (Å)		
Type	X	Y	Z	Type	X	Y	Z
C	1.38722	2.26044	1.36086	H	1.7097	-0.124	-1.2652
C	1.4393	0.92473	1.3241	H	0.23436	-1.0843	-1.23
C	0.40288	0.29521	0.40915	H	1.15781	-2.4564	0.69872
C	-0.248	1.53653	-0.2995	H	3.40874	-3.0573	0.44671
C	0.32175	2.76	0.46544	H	4.50687	-2.2284	-1.6721
O	-0.0199	3.92183	0.34325	H	3.70831	-0.6727	-1.5085
C	1.04744	-0.6898	-0.602	H	4.93228	-0.2689	0.64023
C	1.77316	-1.8407	0.04531	H	5.71049	-1.8388	0.50754
C	3.06019	-2.1811	-0.1039	H	6.75126	-1.1329	-1.678
C	4.12254	-1.5085	-0.9325	H	6.03408	0.46141	-1.5005
C	5.30681	-1.0036	-0.084	H	8.06092	-0.7894	0.43658
C	6.43134	-0.3923	-0.9309	H	8.45066	0.35492	-0.8362
C	7.66168	0.06459	-0.1294	H	8.32251	1.54738	1.32768
C	7.39899	1.23337	0.82796	H	7.00084	2.10244	0.28912
O	-0.501	-0.3825	1.28077	H	6.6775	0.96899	1.60911
C	-1.7839	1.52349	-0.3862	H	-0.895	-1.1523	0.82466
C	-2.3313	0.37384	-1.19	H	-2.2173	1.55383	0.61119
C	-3.3771	-0.3959	-0.8648	H	-1.8329	0.20251	-2.1447
C	-4.2367	-0.2993	0.37296	H	-3.6537	-1.1776	-1.5747
O	-2.1521	2.74881	-1.0851	H	-4.2168	0.72248	0.76487
C	-3.8593	-1.2678	1.51716	H	-5.2779	-0.4978	0.08031
C	-3.9238	-2.7599	1.15853	H	-4.5575	-1.0948	2.34487
C	-2.7645	-3.2743	0.32892	H	-2.8621	-1.0211	1.89329
C	-3.0622	3.55638	-0.4946	H	-4.8593	-3.0147	0.64848
O	-3.6981	3.26606	0.49718	H	-3.91	-3.3582	2.08124
C	-3.1476	4.87039	-1.2304	H	-2.2205	5.42404	-1.0461
O	-3.1236	-4.3378	-0.4104	H	-3.2292	4.70717	-2.3091
O	-1.6244	-2.8416	0.33846	H	-4.0008	5.44352	-0.8656
C	-2.0744	-4.9545	-1.1801	H	-2.5481	-5.7865	-1.7008
H	1.99415	2.92868	1.96204	H	-1.6546	-4.2412	-1.8937
H	2.1022	0.28936	1.90256	H	-1.278	-5.3129	-0.5231
H	0.13594	1.6177	-1.3267				

Conformer 10							
Atomic	Standard Orientation (Å)			Atomic	Standard Orientation (Å)		
Type	X	Y	Z	Type	X	Y	Z
C	0.66841	4.3221	0.04909	H	-1.205	1.98758	-2.5488
C	0.30068	3.73422	-1.0952	H	-1.8826	2.12444	-0.9238
C	0.28502	2.21638	-1.0313	H	-0.3927	-0.3861	-1.9844
C	0.57356	1.92993	0.48276	H	-1.9725	-1.6722	-0.8822
C	0.95726	3.30139	1.08096	H	-3.2269	-0.5377	0.99175
O	1.38629	3.50251	2.20117	H	-3.3119	0.95823	0.07198
C	-1.0822	1.65593	-1.5075	H	-4.7013	-0.1957	-1.6726
C	-1.1707	0.15403	-1.4445	H	-4.5898	-1.7017	-0.7755
C	-2.089	-0.5901	-0.8121	H	-5.7595	-0.6296	1.17917
C	-3.2917	-0.1334	-0.0304	H	-5.8757	0.87838	0.28452
C	-4.6177	-0.6087	-0.6579	H	-7.265	-0.2698	-1.4701
C	-5.8512	-0.2151	0.16393	H	-7.15	-1.7765	-0.5755
C	-7.1739	-0.684	-0.456	H	-9.3294	-0.6364	-0.0988
C	-8.4021	-0.2875	0.36974	H	-8.357	-0.7161	1.37858
O	1.32018	1.80661	-1.925	H	-8.4728	0.80201	0.47722
C	1.55271	0.78953	0.8152	H	1.48437	0.85096	-1.832
C	1.57557	0.41143	2.27501	H	1.31748	-0.0775	0.1976
C	0.9994	-0.6683	2.81749	H	2.10601	1.11571	2.90936
C	0.16855	-1.726	2.13811	H	1.12475	-0.8037	3.8935
O	2.91409	1.21724	0.48935	H	-0.7867	-1.8031	2.6781
C	0.82228	-3.1258	2.14037	H	-0.0815	-1.4505	1.11156
C	2.08963	-3.2385	1.28527	H	0.08457	-3.8565	1.78865
C	1.83734	-3.1887	-0.2112	H	1.07765	-3.4051	3.17117
C	3.56803	0.59425	-0.5028	H	2.62767	-4.169	1.50115
O	3.09275	-0.2962	-1.1884	H	2.79124	-2.4278	1.52439
C	4.95013	1.1669	-0.6887	H	4.86396	2.10112	-1.2558
O	2.94863	-3.5348	-0.8889	H	5.41301	1.39979	0.27324
O	0.79002	-2.8843	-0.7485	H	5.5639	0.46483	-1.2548
C	2.86855	-3.4283	-2.3213	H	2.00998	-3.9873	-2.7015
H	0.77818	5.38452	0.23742	H	2.78528	-2.378	-2.6071
H	0.05779	4.24102	-2.0253	H	3.8003	-3.8539	-2.6961
H	-0.3735	1.64789	0.9615				

Conformer 11							
Atomic	Standard Orientation (Å)			Atomic	Standard Orientation (Å)		
Type	X	Y	Z	Type	X	Y	Z
C	1.00313	1.44384	1.42443	H	2.31412	0.17301	-1.6542
C	1.41723	0.23939	1.01189	H	1.11992	-1.0041	-2.2048
C	0.72446	-0.2463	-0.2498	H	2.06953	-2.6942	-0.5156
C	-0.1211	0.99926	-0.6982	H	4.4095	-2.8463	-0.677
C	-0.0034	1.99141	0.48978	H	5.32136	-1.275	-2.5048
O	-0.6023	3.0429	0.62663	H	4.40498	0.04255	-1.7935
C	1.72434	-0.6942	-1.3401	H	6.6127	-1.561	-0.3814
C	2.60796	-1.8395	-0.9202	H	6.82159	0.01281	-1.1351
C	3.94198	-1.9183	-1.0142	H	5.18549	1.01246	0.49045
C	4.91273	-0.8995	-1.5538	H	4.92917	-0.5709	1.20644
C	6.0954	-0.6155	-0.604	H	6.42373	0.65536	2.64688
C	5.68359	0.05631	0.71277	H	7.33934	-0.6503	1.91379
C	6.84172	0.30517	1.69334	H	8.64917	1.49543	1.9701
C	7.88216	1.32138	1.20669	H	8.3944	0.98161	0.29955
O	-0.0445	-1.4052	0.06345	H	7.41333	2.28748	0.98059
C	-1.5725	0.71396	-1.1286	H	-0.6805	-1.2225	0.78552
C	-1.7029	-0.1841	-2.3309	H	-2.1402	0.3317	-0.2843
C	-2.5758	-1.1914	-2.4559	H	-1.0461	0.05528	-3.1671
C	-3.5586	-1.6496	-1.4063	H	-2.5665	-1.7566	-3.3885
O	-2.1443	2.00737	-1.4972	H	-3.8212	-0.8053	-0.7598
C	-3.0164	-2.8215	-0.5474	H	-4.4947	-1.9566	-1.8911
C	-3.6852	-2.8997	0.83641	H	-1.9387	-2.7008	-0.4152
C	-3.3841	-1.6665	1.66958	H	-3.168	-3.7742	-1.0685
C	-3.2075	2.4474	-0.7931	H	-3.2954	-3.7673	1.38546
O	-3.8142	1.78615	0.02733	H	-4.7699	-3.0202	0.75928
C	-3.5222	3.87741	-1.1525	H	-2.7573	4.51428	-0.695
O	-4.4775	-1.1679	2.25233	H	-3.4798	4.02659	-2.235
O	-2.2757	-1.1669	1.80567	H	-4.5058	4.14867	-0.7666
C	-4.2993	0.05991	2.99538	H	-5.2695	0.25937	3.45038
H	1.31213	1.98024	2.31507	H	-3.5307	-0.0673	3.761
H	2.13798	-0.3993	1.51302	H	-4.019	0.86152	2.30909
H	0.37952	1.48666	-1.5468				

Table S10. Energy analyses of 7*S*,8*R*,12*S*-**2**/7*R*,8*S*,12*R*-**2** (five conformers)

**NO.**	**3D conformers**<br>B3LYP/6-31G(d,p)	**G (Hartree)**	**Boltzmann distribution**	**Calculated ECD spectrum**<br>7*S*,8*R*,12*S*-**2**
1	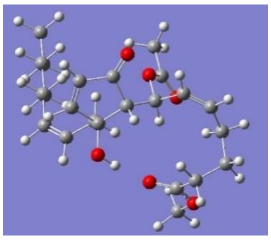	-1347.771487	80.14 %	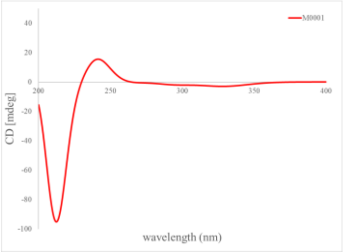
2	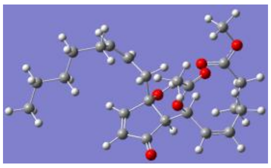	-1347.768911	5.24 %	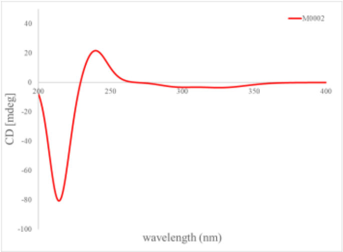
3	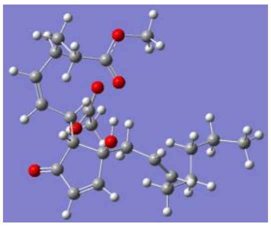	-1347.769451	9.28 %	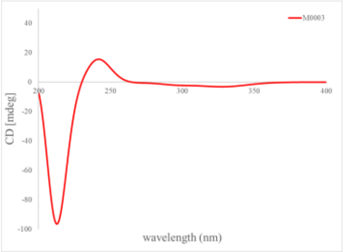
4	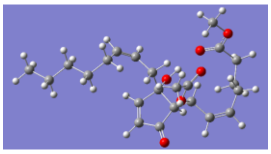	-1347.768154	2.35 %	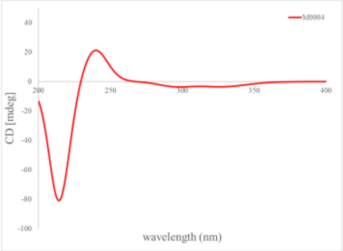
5	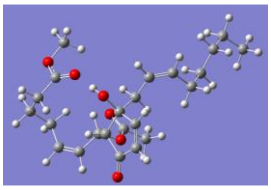	-1347.768328	2.82 %	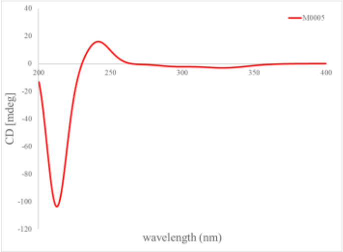

Table S11. Cartesian coordinates of the low-energy re-optimized conformers of 7*S*,8*R*,12*S*-**2**/7*R*,8*S*,12*R*-**2** calculated at B3LYP/6-31G(d,p) level of theory.

Conformer 1							
Atomic	Standard Orientation (Å)			Atomic	Standard Orientation (Å)		
Type	X	Y	Z	Type	X	Y	Z
C	-0.1847	3.67554	1.19432	H	-1.3064	0.7008	0.32669
C	-0.3307	2.64492	2.03732	H	-0.1865	-0.5734	0.80352
C	0.34759	1.37156	1.57032	H	-1.0607	-0.3857	3.21422
C	1.23631	1.88519	0.38482	H	-3.4085	-0.4401	3.28872
C	0.6948	3.29695	0.06811	H	-4.592	1.03998	1.63154
O	0.97972	3.98061	-0.8985	H	-3.4008	0.66479	0.39572
C	-0.7228	0.31588	1.16648	H	-4.1135	-1.7435	0.44264
C	-1.6045	-0.0846	2.32057	H	-5.2809	-1.3809	1.70387
C	-2.943	-0.1047	2.35973	H	-6.4772	0.19783	0.14981
C	-3.9183	0.24843	1.26805	H	-5.3145	-0.1656	-1.1162
C	-4.7754	-0.9543	0.82523	H	-6.0199	-2.5798	-1.0713
C	-5.8204	-0.5952	-0.2383	H	-7.1826	-2.2166	0.19356
C	-6.6764	-1.7881	-0.6832	H	-8.3122	-2.2942	-2.0414
C	-7.7181	-1.422	-1.7455	H	-8.4113	-0.6572	-1.374
O	1.13662	0.8877	2.65085	H	-7.2401	-1.0228	-2.6486
C	1.47626	0.98377	-0.8331	H	1.58475	0.07246	2.34919
C	2.68184	1.42697	-1.6242	H	1.58447	-0.0512	-0.5104
C	3.69691	0.65121	-2.0261	H	2.69521	2.48857	-1.8577
C	3.8768	-0.8376	-1.8649	H	4.51142	1.1488	-2.5564
O	0.28549	1.0569	-1.6819	H	2.92531	-1.3251	-1.6291
C	4.95973	-1.2301	-0.8348	H	4.18371	-1.2414	-2.8395
C	4.53499	-1.0182	0.63087	H	5.87045	-0.645	-1.0154
C	3.37492	-1.8936	1.05882	H	5.22654	-2.2828	-0.9747
C	-0.0595	-0.0567	-2.3642	H	4.24858	0.02048	0.81582
O	0.50692	-1.1263	-2.2685	H	5.38086	-1.2511	1.29119
C	-1.2514	0.21413	-3.253	H	-2.0919	0.58782	-2.6591
O	3.58656	-3.1866	0.77485	H	-1.0004	0.99023	-3.9834
O	2.3546	-1.5061	1.60704	H	-1.5375	-0.7034	-3.7683
C	2.52392	-4.0954	1.12527	H	2.87674	-5.0813	0.82328
H	-0.6136	4.66744	1.28704	H	2.33524	-4.064	2.20099
H	-0.8946	2.64901	2.96464	H	1.60927	-3.831	0.58941
H	2.21918	2.05437	0.84533				

Conformer 2							
Atomic	Standard Orientation (Å)			Atomic	Standard Orientation (Å)		
Type	X	Y	Z	Type	X	Y	Z
C	-1.9169	-2.2156	-1.2298	H	-1.2249	0.31534	0.66134
C	-1.7238	-0.986	-1.7281	H	0.20267	1.19552	0.11713
C	-0.4359	-0.3371	-1.2579	H	-1.2486	2.2745	-1.7331
C	0.34126	-1.5658	-0.6743	H	-3.1798	3.2364	-0.8023
C	-0.7398	-2.6367	-0.4364	H	-2.9192	1.20959	1.5245
O	-0.6251	-3.6478	0.23201	H	-3.2719	2.91605	1.74694
C	-0.7568	0.77229	-0.2142	H	-5.3529	1.69844	1.88104
C	-1.6054	1.87974	-0.7833	H	-5.3804	2.68319	0.42592
C	-2.7066	2.42268	-0.2481	H	-4.7849	0.66632	-0.9519
C	-3.3943	2.075	1.04688	H	-4.7424	-0.3187	0.50035
C	-4.9042	1.80313	0.88329	H	-7.174	0.18258	0.91324
C	-5.2284	0.55881	0.04784	H	-7.2173	1.16974	-0.5385
C	-6.7327	0.29161	-0.0879	H	-8.1334	-1.1158	-1.0022
C	-7.0525	-0.952	-0.9239	H	-6.6556	-0.8575	-1.9423
O	0.22315	0.22133	-2.3878	H	-6.6106	-1.8524	-0.4795
C	1.35668	-1.3489	0.45098	H	0.99511	0.73107	-2.0705
C	2.34403	-2.4831	0.55777	H	1.87695	-0.4075	0.28068
C	3.66137	-2.3564	0.35331	H	1.90626	-3.4536	0.77715
C	4.37651	-1.0635	0.02966	H	4.27358	-3.2561	0.40985
O	0.63448	-1.2178	1.7165	H	3.95481	-0.2631	0.64959
C	4.29255	-0.6638	-1.4629	H	5.43188	-1.1433	0.31851
C	4.66352	0.81273	-1.7072	H	3.27527	-0.8242	-1.8356
C	3.66138	1.72174	-1.0242	H	4.949	-1.3042	-2.0636
C	1.11288	-0.3276	2.6101	H	4.62934	1.02671	-2.7823
O	2.05802	0.41117	2.4109	H	5.66993	1.04013	-1.3448
C	0.30642	-0.3692	3.88695	H	-0.7478	-0.1626	3.67551
O	4.22895	2.52489	-0.1192	H	0.35871	-1.3704	4.32681
O	2.45799	1.70991	-1.241	H	0.69666	0.36769	4.58975
C	3.32976	3.33819	0.66759	H	3.97592	3.98512	1.26068
H	-2.7849	-2.8513	-1.367	H	2.68073	3.92794	0.01676
H	-2.4063	-0.4398	-2.3714	H	2.7284	2.69466	1.31401
H	0.91551	-1.9365	-1.5343				

Conformer 3							
Atomic	Standard Orientation (Å)			Atomic	Standard Orientation (Å)		
Type	X	Y	Z	Type	X	Y	Z
C	-0.1017	3.84985	-1.183	H	1.34847	1.1338	-0.0234
C	0.32611	2.83743	-1.9486	H	0.53394	-0.3244	-0.5851
C	-0.1955	1.48082	-1.516	H	1.70738	-0.0716	-2.8516
C	-1.3167	1.86919	-0.491	H	4.01365	0.30141	-2.6111
C	-1.0578	3.35762	-0.1685	H	4.62628	2.07686	-0.9255
O	-1.5805	4.00713	0.71902	H	3.41259	1.47259	0.19241
C	0.96368	0.63296	-0.9149	H	5.8539	-0.1005	-0.7979
C	2.05707	0.3562	-1.9136	H	5.76315	0.75284	0.736
C	3.37019	0.58051	-1.7737	H	3.84285	-0.7056	1.43986
C	4.1085	1.16323	-0.5965	H	3.95885	-1.5945	-0.0694
C	5.16095	0.20246	-0.001	H	6.17566	-1.421	2.05055
C	4.55306	-1.0375	0.66813	H	5.03704	-2.7471	1.88817
C	5.57906	-1.9818	1.31635	H	7.18705	-3.3773	0.83834
C	6.51751	-2.6787	0.32341	H	5.94866	-3.2503	-0.4208
O	-0.7307	0.84149	-2.6686	H	7.14548	-1.9629	-0.219
C	-1.5865	0.9683	0.72101	H	-1.0777	-0.0298	-2.3911
C	-2.9484	1.22977	1.3148	H	-1.4843	-0.0775	0.43295
C	-3.8775	0.31024	1.60645	H	-3.1616	2.28055	1.49401
C	-3.7965	-1.1916	1.49309	H	-4.8283	0.68262	1.99317
O	-0.5572	1.24981	1.72342	H	-2.7559	-1.5244	1.4229
C	-4.6351	-1.7803	0.33635	H	-4.1811	-1.6154	2.43098
C	-4.0289	-1.5426	-1.0597	H	-5.6423	-1.3447	0.35119
C	-2.6937	-2.2281	-1.2668	H	-4.7537	-2.858	0.48887
C	-0.1422	0.2199	2.49268	H	-3.8844	-0.4766	-1.2548
O	-0.5152	-0.9286	2.36598	H	-4.7159	-1.9283	-1.8245
C	0.85964	0.69514	3.51953	H	1.73201	1.13201	3.02188
O	-2.7362	-3.527	-0.9384	H	0.41477	1.47861	4.14128
O	-1.6783	-1.6963	-1.689	H	1.17064	-0.1453	4.14103
C	-1.5015	-4.2577	-1.0785	H	-1.7333	-5.2768	-0.7698
H	0.17004	4.89675	-1.264	H	-1.1614	-4.2351	-2.1166
H	1.00668	2.91238	-2.7907	H	-0.7319	-3.8243	-0.4356
H	-2.2362	1.86502	-1.0921				

Conformer 4							
Atomic	Standard Orientation (Å)			Atomic	Standard Orientation (Å)		
Type	X	Y	Z	Type	X	Y	Z
C	-1.5075	-2.4158	-1.5313	H	-1.1219	-0.1171	0.7086
C	-1.4961	-1.1123	-1.8449	H	0.15385	1.0295	0.29237
C	-0.2987	-0.3659	-1.2877	H	-1.4548	2.18299	-1.337
C	0.6526	-1.5462	-0.8924	H	-3.544	2.61325	-0.3604
C	-0.2658	-2.7798	-0.811	H	-2.8492	0.47463	1.75774
O	0.00164	-3.8513	-0.299	H	-3.9414	1.83469	1.95888
C	-0.7449	0.52529	-0.0909	H	-4.428	-0.5828	0.13832
C	-1.7567	1.56736	-0.4913	H	-5.1258	-0.3432	1.73604
C	-2.9498	1.80617	0.06932	H	-6.2952	1.73391	0.898
C	-3.5888	1.08812	1.23109	H	-5.6533	1.43254	-0.711
C	-4.7822	0.188	0.83674	H	-6.8373	-0.7836	-0.77
C	-5.9703	0.93372	0.21533	H	-7.4869	-0.4782	0.83249
C	-7.1631	0.01902	-0.093	H	-9.1836	0.08185	-0.9238
C	-8.3495	0.7618	-0.7159	H	-8.72	1.54863	-0.0472
O	0.25166	0.43647	-2.3248	H	-8.0657	1.23962	-1.6618
C	1.65432	-1.3547	0.24969	H	0.95208	0.99861	-1.9378
C	2.79191	-2.3417	0.1849	H	2.03463	-0.3347	0.21852
C	4.0735	-2.0021	-0.0033	H	2.49866	-3.3857	0.26038
C	4.59403	-0.588	-0.135	H	4.80598	-2.8051	-0.0822
O	0.951	-1.512	1.52269	H	4.08082	0.04569	0.59839
C	4.41883	0.00938	-1.5516	H	5.65708	-0.5597	0.13471
C	4.57675	1.54305	-1.5764	H	3.42445	-0.2355	-1.9399
C	3.47543	2.19331	-0.7632	H	5.14219	-0.4384	-2.243
C	1.32108	-0.7016	2.53578	H	4.48765	1.90449	-2.608
O	2.14986	0.18356	2.44458	H	5.55056	1.8537	-1.1877
C	0.556	-1.039	3.79405	H	0.74496	-2.0796	4.07673
O	3.94812	2.92644	0.24931	H	0.86424	-0.3712	4.5992
O	2.28033	2.04543	-0.9759	H	-0.5205	-0.941	3.6193
C	2.96437	3.48186	1.15084	H	3.52917	4.11842	1.83173
H	-2.2823	-3.14	-1.7588	H	2.22531	4.06451	0.59682
H	-2.2609	-0.5781	-2.3996	H	2.47263	2.67283	1.69606
H	1.25228	-1.7052	-1.799				

Conformer 5							
Atomic	Standard Orientation (Å)			Atomic	Standard Orientation (Å)		
Type	X	Y	Z	Type	X	Y	Z
C	-0.0578	3.69214	-1.3962	H	1.25863	0.98878	-0.0556
C	0.3247	2.61364	-2.0925	H	0.37855	-0.4612	-0.5301
C	-0.2623	1.31356	-1.5778	H	1.56784	-0.3885	-2.8097
C	-1.3675	1.81919	-0.587	H	3.88777	-0.1421	-2.5622
C	-1.0406	3.31171	-0.3595	H	4.73974	1.52688	-0.9458
O	-1.5364	4.04094	0.48027	H	3.31731	1.36587	0.07091
C	0.85359	0.45236	-0.9172	H	5.16969	0.2795	1.16347
C	1.93595	0.06301	-1.89	H	4.01116	-0.9604	0.69298
C	3.25945	0.21318	-1.7449	H	5.41556	-1.5289	-1.3029
C	4.00207	0.80091	-0.5717	H	6.57662	-0.2878	-0.852
C	4.74336	-0.2427	0.29669	H	5.87284	-2.7486	0.84408
C	5.85159	-1.0072	-0.441	H	7.25841	-2.6264	-0.2263
C	6.59836	-2.0369	0.42443	H	7.98331	-2.2114	2.1008
C	7.43491	-1.4324	1.55867	H	8.17127	-0.7187	1.16779
O	-0.8235	0.6278	-2.6907	H	6.81435	-0.9011	2.28888
C	-1.6824	1.01222	0.67914	H	-1.2098	-0.2079	-2.3606
C	-3.0336	1.37204	1.24543	H	-1.6259	-0.0537	0.46054
C	-4.0034	0.51543	1.59104	H	-3.2006	2.44069	1.35394
C	-3.9884	-0.9928	1.57697	H	-4.9382	0.95382	1.94633
O	-0.6462	1.31262	1.66887	H	-2.9633	-1.3748	1.53569
C	-4.8478	-1.6189	0.45557	H	-4.3951	-1.3366	2.53796
C	-4.226	-1.5007	-0.9488	H	-5.8346	-1.1394	0.43477
C	-2.921	-2.2554	-1.1012	H	-5.0147	-2.678	0.67779
C	-0.2776	0.3164	2.5034	H	-4.0344	-0.4573	-1.2131
O	-0.6938	-0.8227	2.44265	H	-4.9261	-1.9062	-1.6913
C	0.728	0.81663	3.51447	H	1.59164	1.25815	3.00688
O	-3.0203	-3.5258	-0.6852	H	0.27574	1.60283	4.12804
O	-1.8827	-1.7974	-1.5528	H	1.04971	-0.0104	4.14836
C	-1.8177	-4.3164	-0.7681	H	-2.0944	-5.3016	-0.3932
H	0.26361	4.71771	-1.5422	H	-1.4731	-4.3782	-1.803
H	1.01172	2.60234	-2.9326	H	-1.0326	-3.8741	-0.1504
H	-2.2844	1.81855	-1.1922				

Table S12. Experimental and calculated ^{1}H NMR data for compound **2**.

No.	**2**, exptl. δ_H [a]	7*S*,8*R*,12*R*-**2**, calcd. δ_H [b]	7*S*,8*R*,12*S*-**2**, calcd. δ_H [b]
2	2.34	2.48	2.38
3	1.72	1.88	1.89
4	2.22	2.40	2.46
5	5.57	6.10	6.02
6	5.85	6.55	7.01
7	5.94	5.81	5.90
8	2.55	2.41	2.71
10	6.17	6.40	6.36
11	7.43	7.94	8.09
13	2.56	3.11	2.58
	2.34	2.40	2.48
14	5.36	5.70	6.42
15	5.65	6.01	6.07
16	2	2.25	2.05
17	1.35	1.41	1.31
18	1.27	1.29	1.28
19	1.3	1.38	1.40
20	0.89	1.02	1.02
1-OMe	3.68	4.04	3.94
2′	1.99	2.06	2.02

[a] Recorded in $CDCl_3$ at 600 MHz.

[b] Calculated in $CDCl_3$

Table S13. Experimental and calculated ^{13}C NMR data for compound **2**.

No.	**2**, exptl. δ_C [a]	7*S*,8*R*,12*R*-**2**, calcd. δ_C [b]	7*S*,8*R*,12*S*-**2**, calcd. δ_C [b]
1	174	178.7	180.1
2	33.4	30.5	31.3
3	24.5	23.1	23.1
4	27	24.4	23.9
5	133.3	136.5	134.6
6	129.6	130.8	131.3
7	68.3	65.0	63.7
8	57.2	52.5	61.9
9	204.8	206.9	204.0
10	133.7	134.1	132.6
11	165.4	171.6	172.8
12	79.5	79.0	79.9
13	39.3	36.5	33.4
14	121.8	125.3	126.8
15	135.7	136.7	133.7
16	27.4	26.3	27.1
17	29.1	28.0	28.6
18	31.5	29.6	30.4
19	22.5	21.8	21.9
20	14	10.4	10.4
1-OMe	51.6	48.9	49.8
1′	170.3	170.2	171.9
2′	21.2	17.0	18.0

[a] Recorded in $CDCl_3$ at 600 MHz.

[b] Calculated in $CDCl_3$

Table S14. DP4+ analyses of calculated and experimental NMR chemical shifts of **2** (unscaled). Isomer 1: 7*S*,8*R*,12*R*-**2**; Isomer 2: 7*S*,8*R*,12*S*-**2**

Functional	Solvent?	Basis Set	Type of Data			
mPW1PW91	PCM	6-311+G(d,p)	Unscaled Shifts			
	Isomer 1	Isomer 2	Isomer 3	Isomer 4	Isomer 5	Isomer 6
sDP4+ (H data)	83.83%	16.17%	-	-	-	-
sDP4+ (C data)	100.00%	0.00%	-	-	-	-
sDP4+ (all data)	100.00%	0.00%	-	-	-	-
uDP4+ (H data)	99.02%	0.98%	-	-	-	-
uDP4+ (C data)	3.91%	96.09%	-	-	-	-
uDP4+ (all data)	80.49%	19.51%	-	-	-	-
DP4+ (H data)	99.81%	0.19%	-	-	-	-
DP4+ (C data)	100.00%	0.00%	-	-	-	-
DP4+ (all data)	100.00%	0.00%	-	-	-	-

Functional	Solvent?	Basis Set	Type of Data
mPW1PW91	PCM	6-311+G(d,p)	Unscaled Shifts

		DP4+	100.00%	0.00%	-	-	-
Nuclei	sp2?	Experimenta	Isomer 1	Isomer 2	Isomer 3	Isomer 4	Isomer 5
C	x	174.0	178.7	180.1			
C		33.4	30.5	31.3			
C		24.5	23.1	23.1			
C		27.0	24.4	23.9			
C	x	133.3	136.5	134.6			
C	x	129.6	130.8	131.3			
C		68.3	65.0	63.7			
C		57.2	52.5	61.9			
C	x	204.8	206.9	204.0			
C	x	133.7	134.1	132.6			
C	x	165.4	171.6	172.8			
C		79.5	78.98	79.91			
C		39.3	36.45	33.45			
C	x	121.8	125.30	126.77			
C	x	135.7	136.73	133.75			
C		27.4	26.28	27.10			
C		29.1	27.98	28.59			
C		31.5	29.58	30.36			
C		22.5	21.79	21.89			
C		14.0	10.40	10.36			
C		51.6	48.87	49.75			
C	x	170.3	170.22	171.86			
C		21.2	17.03	17.99			
H		2.3	2.48	2.38			
H		1.7	1.88	1.89			
H		2.22	2.3999751	2.45825543			
H	x	5.57	6.10101227	6.02000694			
H	x	5.85	6.55245404	7.01225953			
H		5.94	5.81206222	5.89869416			
H		2.55	2.4114666	2.71207999			
H	x	6.17	6.40119312	6.35704911			
H	x	7.43	7.94088827	8.0875924			
H		2.56	3.11240124	2.58422038			
H		2.34	2.40440269	2.48382623			
H	x	5.36	5.70232067	6.42483296			
H	x	5.65	6.00938708	6.07013901			
H		2.00	2.2537362	2.05059299			
H		1.35	1.41051741	1.31460266			
H		1.27	1.29085926	1.27875256			
H		1.30	1.37931264	1.39857795			
H		0.89	1.01984823	1.02190545			
H		3.68	4.04144572	3.94368397			
H		1.99	2.05722616	2.02193488			

Table S15. *In silico* prediction of cytotoxicity of the compounds for human tumor cell lines.

	Cell-line	Description	Tissue/Organ	*Pa*	*Pi*	IAP
**1**	A2780cisR	Cisplatin-resistant ovarian carcinoma	Ovarium	0.814	0.026	0.838
**1**	PC-3	Prostate carcinoma	Prostate	0.798	0.005	0.883
**2**	A2780cisR	Cisplatin-resistant ovarian carcinoma	Ovarium	0.928	0.004	0.838
**3**	A2780cisR	Cisplatin-resistant ovarian carcinoma	Ovarium	0.896	0.006	0.838
**3**	PC-3	Prostate carcinoma	Prostate	0.767	0.007	0.883
**4**	PC-3	Prostate carcinoma	Prostate	0.911	0.004	0.883
**4**	A2780cisR	Cisplatin-resistant ovarian carcinoma	Ovarium	0.904	0.005	0.838
**5**	A2780cisR	Cisplatin-resistant ovarian carcinoma	Ovarium	0.869	0.011	0.838
**5**	PC-3	Prostate carcinoma	Prostate	0.858	0.005	0.883
**6**	PC-3	Prostate carcinoma	Prostate	0.828	0.005	0.883
**6**	A2780cisR	Cisplatin-resistant ovarian carcinoma	Ovarium	0.790	0.035	0.838
**7**	A2780cisR	Cisplatin-resistant ovarian carcinoma	Ovarium	0.869	0.011	0.838
**7**	PC-3	Prostate carcinoma	Prostate	0.858	0.005	0.883
**8**	PC-3	Prostate carcinoma	Prostate	0.982	0.003	0.883
**8**	HT-29	Colon adenocarcinoma	Colon	0.935	0.004	0.888
**8**	A2780cisR	Cisplatin-resistant ovarian carcinoma	Ovarium	0.846	0.017	0.838
**9**	PC-3	Prostate carcinoma	Prostate	0.854	0.005	0.883
**9**	A2780cisR	Cisplatin-resistant ovarian carcinoma	Ovarium	0.747	0.051	0.838
**10**	PC-3	Prostate carcinoma	Prostate	0.920	0.004	0.883
**10**	A2780cisR	Cisplatin-resistant ovarian carcinoma	Ovarium	0.904	0.005	0.838
**11**	PC-3	Prostate carcinoma	Prostate	0.911	0.004	0.883
**11**	A2780cisR	Cisplatin-resistant ovarian carcinoma	Ovarium	0.904	0.005	0.838
**12**	PC-3	Prostate carcinoma	Prostate	0.879	0.005	0.883
**12**	A2780cisR	Cisplatin-resistant ovarian carcinoma	Ovarium	0.859	0.013	0.838
**13**	PC-3	Prostate carcinoma	Prostate	0.879	0.005	0.883
**13**	A2780cisR	Cisplatin-resistant ovarian carcinoma	Ovarium	0.859	0.013	0.838

Pa: the probability of "to be active".

Pi: the probability of "to be inactive".

IAP: Invariant accuracy of prediction.

Table S16. *In silico* prediction of the nitric oxide (NO) production inhibition activity of the compounds.

	**Predictions**
**1**	0.319
**2**	0.329
**3**	0.344
**4**	0.325
**5**	0.365
**6**	0.430
**7**	0.339
**8**	0.326
**9**	0.310
**10**	0.383
**11**	0.345
**12**	0.344
**13**	0.372
**Aminoguanidine**	0.657
**Apigenin**	0.621

Predictions: the predicted probabilities of test compounds with the IC_{50} (inhibition for NO production) < 50 μM in macrophages.

Table S17. The *in silico* predicted water solubility of the compounds.

	ESOL model		Ali model		SILICOS-IT model	
	**Log *S***	**Class**	**Log *S***	**Class**	**Log *S***	**Class**
**1**	-3.48	Soluble	-4.96	Moderately soluble	-3.79	Soluble
**2**	-3.61	Soluble	-5.13	Moderately soluble	-3.79	Soluble
**3**	-4.81	Moderately soluble	-5.82	Moderately soluble	-4.83	Moderately soluble
**4**	-3.99	Soluble	-5.43	Moderately soluble	-4.20	Moderately soluble
**5**	-3.39	Soluble	-4.71	Moderately soluble	-3.26	Soluble
**6**	--	--	--	--	--	--
**7**	-3.39	Soluble	-4.71	Moderately soluble	-3.26	Soluble
**8**	-4.70	Moderately soluble	-5.40	Moderately soluble	-4.64	Moderately soluble
**9**	--	--	--	--	--	--
**10**	-3.50	Soluble	-4.72	Moderately soluble	-3.58	Soluble
**11**	-4.35	Moderately soluble	-6.00	Moderately soluble	-4.60	Moderately soluble
**12**	--	--	--	--	--	--
**13**	--	--	--	--	--	--

Class: solubility class, Log *S* scale, Insoluble ＜ -10 ＜ Poorly ＜ -6 ＜ Moderately ＜ -4 ＜ Soluble ＜ -2 ＜ Very ＜ 0 ＜ Highly.

--: the compounds were not suitable for the *in silico* tool.

Table S18. The *in silico* predicted pharmacokinetics of the compounds.

	**GI absorption**	**BBB permeant**	**P-gp substrate**	**Inhibitors for CYP**					**Log K_p (cm/s)**
				**1A2**	**2C19**	**2C9**	**2D6**	**3A4**	
**1**	High	No	**Yes**	No	**Yes**	**Yes**	**Yes**	No	-7.04
**2**	High	No	**Yes**	No	**Yes**	**Yes**	**Yes**	No	-6.25
**3**	High	No	**Yes**	**Yes**	**Yes**	No	**Yes**	**Yes**	-6.55
**4**	High	Yes	**Yes**	**Yes**	**Yes**	**Yes**	**Yes**	No	-5.56
**5**	High	No	**Yes**	**Yes**	**Yes**	**Yes**	**Yes**	No	-6.45
**6**	--	--	--	--	--	--	--	--	--
**7**	High	No	**Yes**	**Yes**	**Yes**	**Yes**	**Yes**	No	-6.45
**8**	High	Yes	**Yes**	**Yes**	**Yes**	**Yes**	**Yes**	No	-6.01
**9**	--	--	--	--	--	--	--	--	--
**10**	High	Yes	**Yes**	**Yes**	**Yes**	**Yes**	**Yes**	No	-5.71
**11**	High	Yes	**Yes**	**Yes**	**Yes**	**Yes**	**Yes**	**Yes**	-5.26
**12**	--	--	--	--	--	--	--	--	--
**13**	--	--	--	--	--	--	--	--	--

GI absorption: Gastrointestinal absorption; **BBB permeation**: blood-brain barrier permeation; **P-gp substrate**: P-glycoprotein substrate; **CYP**: Cytochrome P450; **Log K_p**: for skin permeation.

--: the compounds were not suitable for the *in silico* tool.

Table S19. The *in silico* evaluation of the compounds for druglikeness.

	**Lipinsk**	**Ghose**	**Veber**	**Egan**	**Muegge**	**Bioavailability score**
**1**	Yes	Yes	**No**; 1 violation: Rotors > 10	Yes	**No**; 1 violation: Rotors > 15	0.55
**2**	Yes	Yes	**No**; 1 violation: Rotors > 10	Yes	Yes	0.55
**3**	**No**; 1 violation: MW > 500	**No**; 1 violation: MW > 480	**No**; 1 violation: Rotors > 10	Yes	Yes	0.55
**4**	Yes	Yes	**No**; 1 violation: Rotors > 10	Yes	Yes	0.55
**5**	Yes	Yes	**No**; 1 violation: Rotors > 10	Yes	Yes	0.55
**6**	--	--	--	--	--	--
**7**	Yes	Yes	**No**; 1 violation: Rotors > 10	Yes	Yes	0.55
**8**	Yes	Yes	**No**; 1 violation: Rotors > 10	Yes	Yes	0.55
**9**	--	--	--	--	--	--
**10**	Yes	Yes	**No**; 1 violation: Rotors > 10	Yes	Yes	0.55
**11**	Yes	Yes	**No**; 1 violation: Rotors > 10	Yes	Yes	0.55
**12**	--	--	--	--	--	--
**13**	--	--	--	--	--	--

--: the compounds were not suitable for the *in silico* tool.

Table S20. The *in silico* evaluation of the compounds for medicinal chemistry.

	PAINS	Brenk	Leadlikeness	Synthetic accessibility
**1**	0 alert	**2 alerts**: ichael acceptor 1, more than 2 esters	**No**; 2 violations: MW > 350, Rotors > 7	5.39
**2**	0 alert	**2 alerts**: isolated alkene, more than 2 esters	**No**; 3 violations: MW > 350, Rotors > 7, XLOGP3 > 3.5	5.22
**3**	**1 alert**: ene one hal	**3 alerts**: iodine, isolated alkene, more than 2 esters	**No**; 3 violations: MW > 350, Rotors > 7, XLOGP3 > 3.5	5.41
**4**	0 alert	**3 alerts**: isolated alkene, michael acceptor 1, more than 2 esters	**No**; 3 violations: MW > 350, Rotors > 7, XLOGP3 > 3.5	4.83
**5**	0 alert	**3 alerts**: isolated alkene, michael acceptor 1, more than 2 esters	**No**; 2 violations: MW > 350, Rotors > 7	5.20
**6**	--	--	--	--
**7**	0 alert	**3 alerts**: isolated alkene, michael acceptor 1, more than 2 esters	**No**; 2 violations: MW > 350, Rotors > 7	5.20
**8**	**1 alert**: ene one hal	**3 alerts**: iodine, isolated alkene, michael acceptor 1	**No**; 3 violations: MW > 350, Rotors > 7, XLOGP3 > 3.5	4.78
**9**	--	--	--	--
**10**	0 alert	**2 alerts**: isolated alkene, michael acceptor 1	**No**; 2 violations: Rotors > 7, XLOGP3 > 3.5	4.58
**11**	0 alert	**3 alerts**: isolated alkene, michael acceptor 1, more than 2 esters	**No**; 3 violations: MW > 350, Rotors > 7, XLOGP3 > 3.5	4.95
**12**	--	--	--	--
**13**	--	--	--	--

PAINS: Pan Assay Interference Structures.

Synthetic score: the synthetic accessibility score; from 1 (very easy) to 10 (very difficult).

--: the compounds were not suitable for the *in silico* tool.

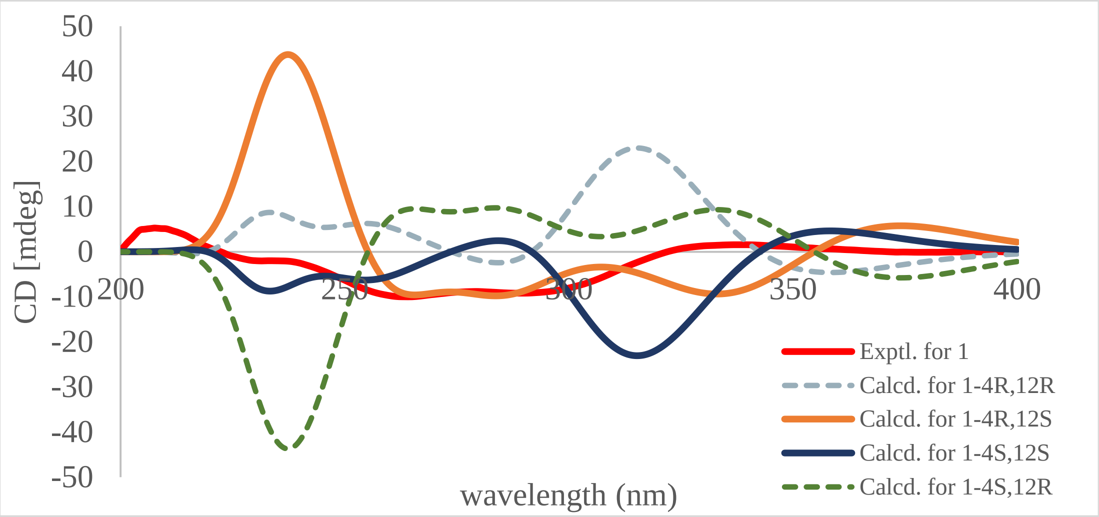

Note: The ECD curves of 4*R*,12*S* and 4*S*,12*S* were generated by software calculation. The curves of 4*R*,12*R* and 4*S*,12*R* are directly transformed from their enantiomers 4*R*,12*S* and 4*S*,12*S*.

Figure S1. Experimental and Calculated ECD of **1**

1

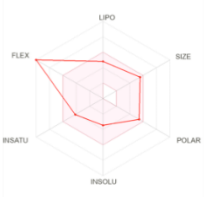

2

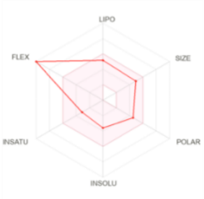

3

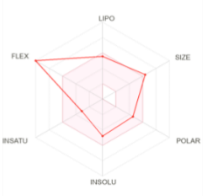

4

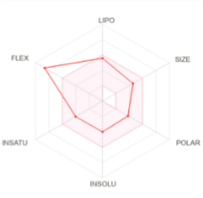

5

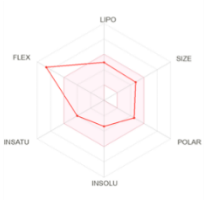

7

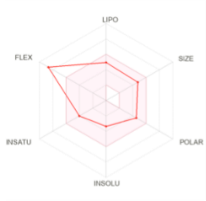

8

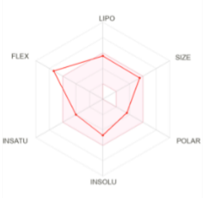

10

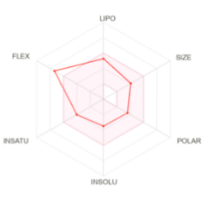

11

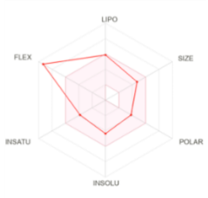

Figure S2. The predicted oral bioavailability of nine compounds.

The colored zone could be the suitable physicochemical space of the ideal compound predicted by the in silico tool SwissADME, and six red spots were the predicted values of the test compound. Lipophility (LIPO): -0.7 ＜ XLOGP3 ＜ +5.0; size (SIZE): 150g/mol ＜ MV ＜ 500 g/mol; polarity (POLAR): 20 Å² ＜ TPSA ＜ 130 Å²; insolubility (INSOLU): -6 ＜ Log S (ESOL) ＜ 0; insaturation (INSATU): 0.25 ＜ Fraction Csp3 ＜ 1; flexibility (FLEX): 0 ＜ Num. rotatable bonds ＜ 9. Almost all of the five values of nine compounds were located in the colored zone, but only one value (size) of compound 3 was not located in the colored zone. Only one value (flexibility) of nine compounds was not located in the colored zone.

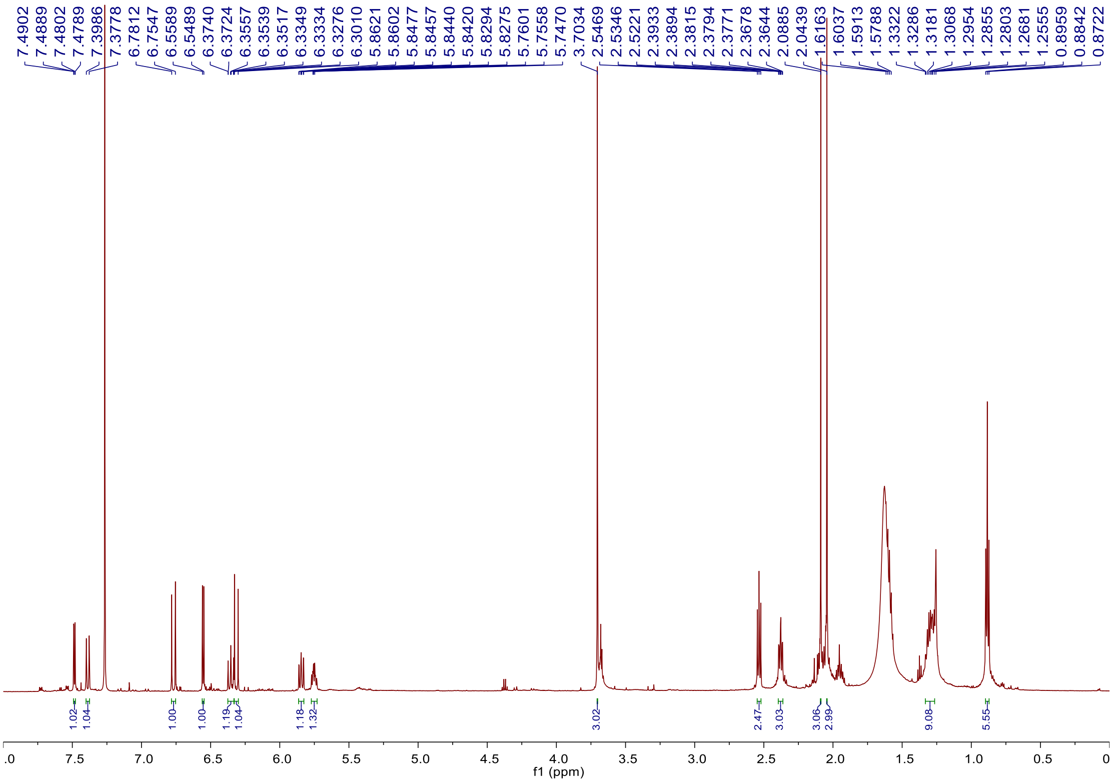

Figure S3. ^{1}H NMR spectrum of **1** (600 MHz, $CDCl_3$)

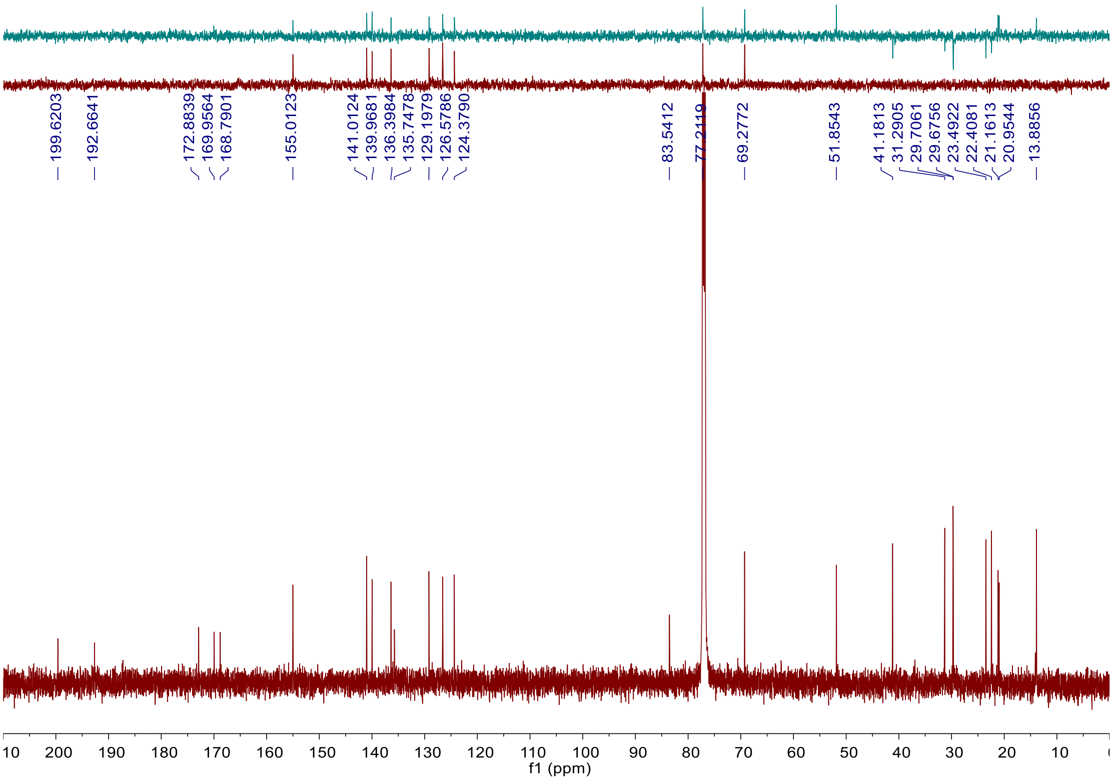

Figure S4. ^{13}C NMR spectrum of **1** (125 MHz, $CDCl_3$)

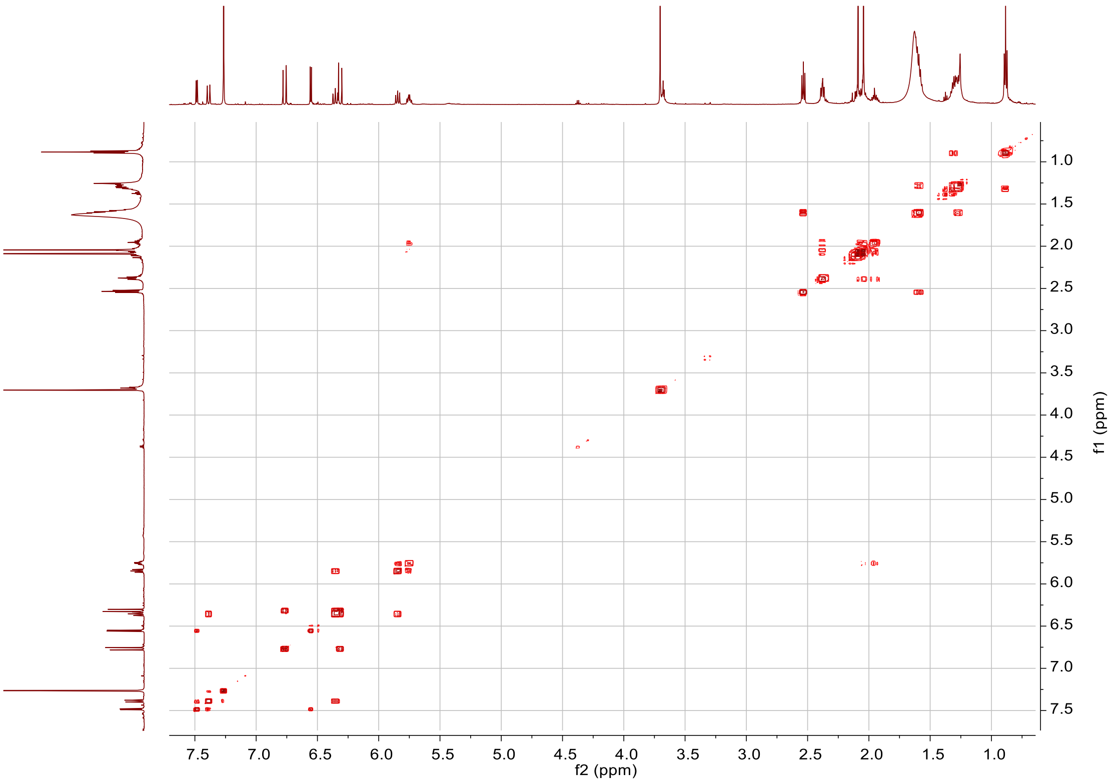

Figure S5. COSY spectrum of **1**

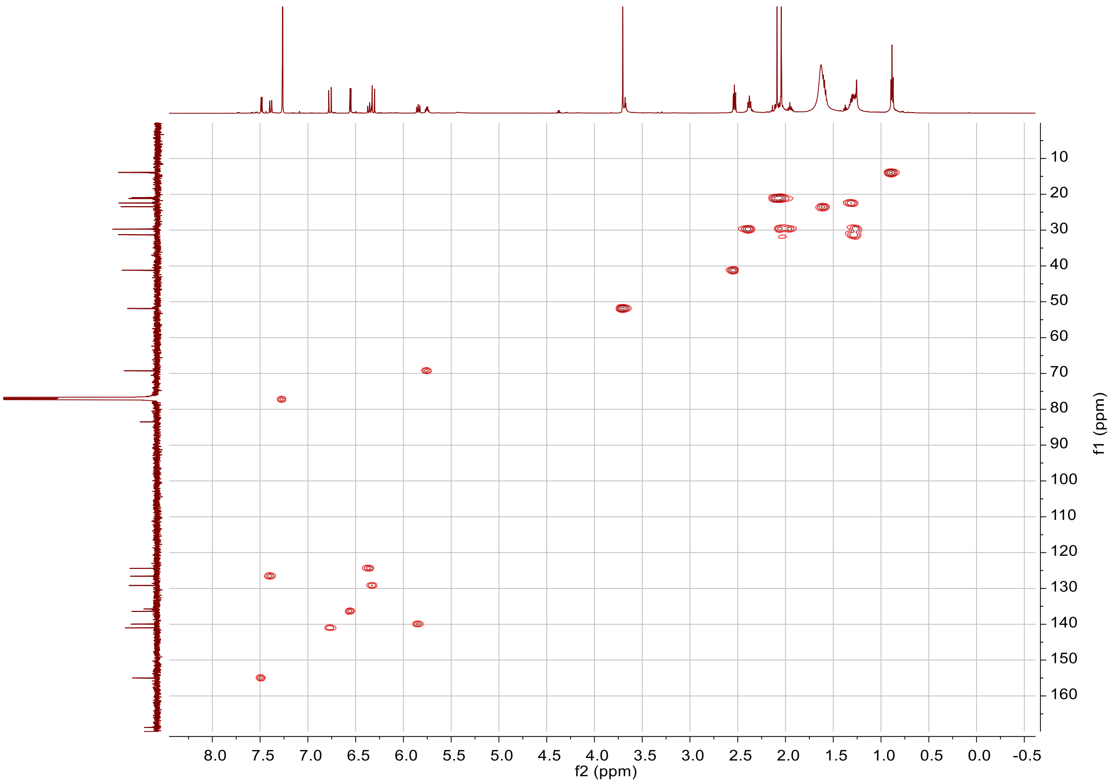

Figure S6. HSQC spectrum of **1**

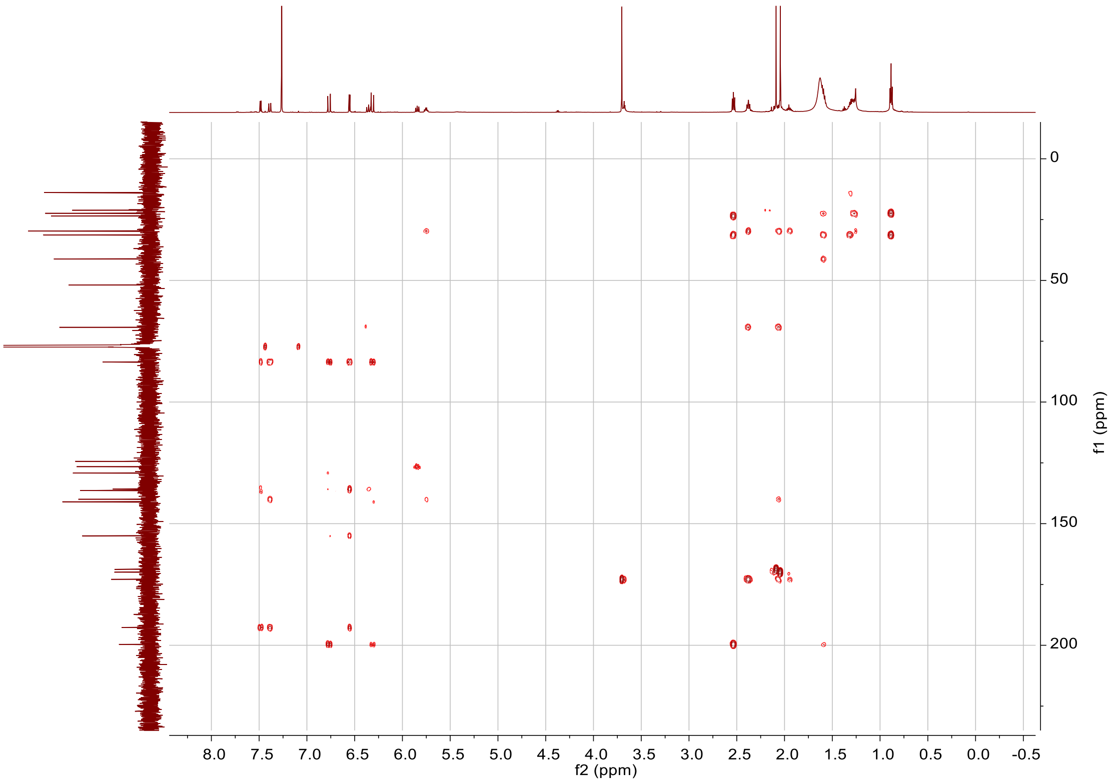

Figure S7. HMBC spectrum of **1**

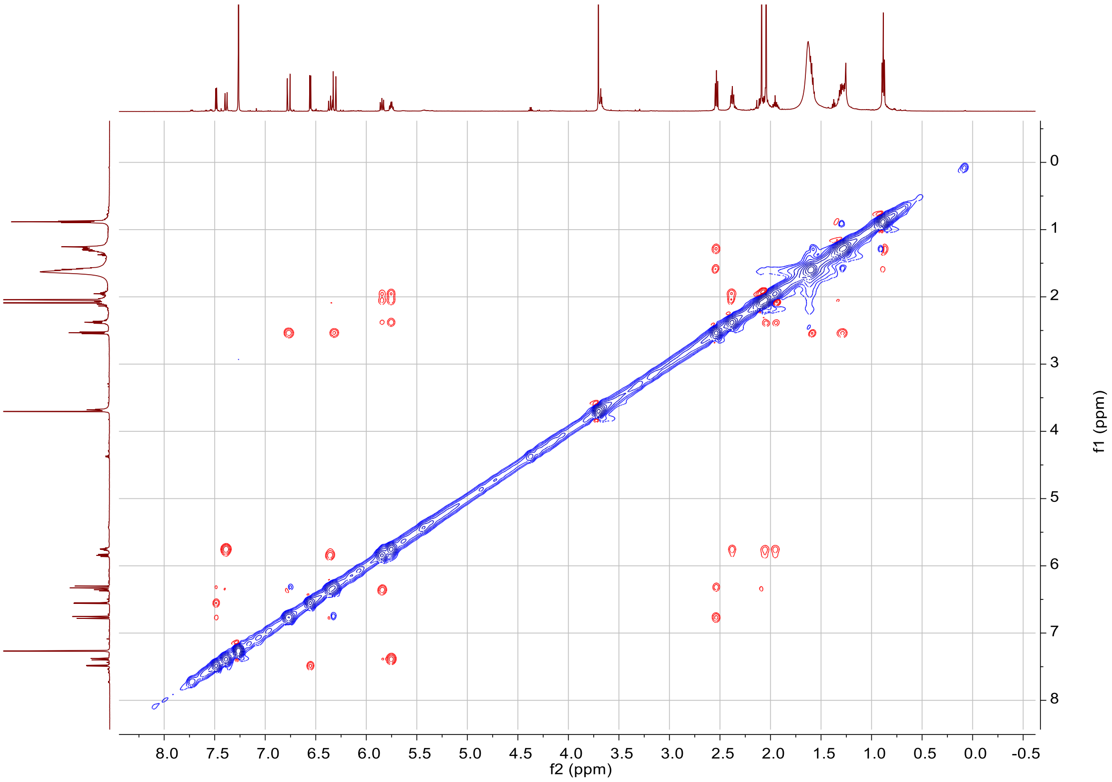

Figure S8. NOESY spectrum of **1**

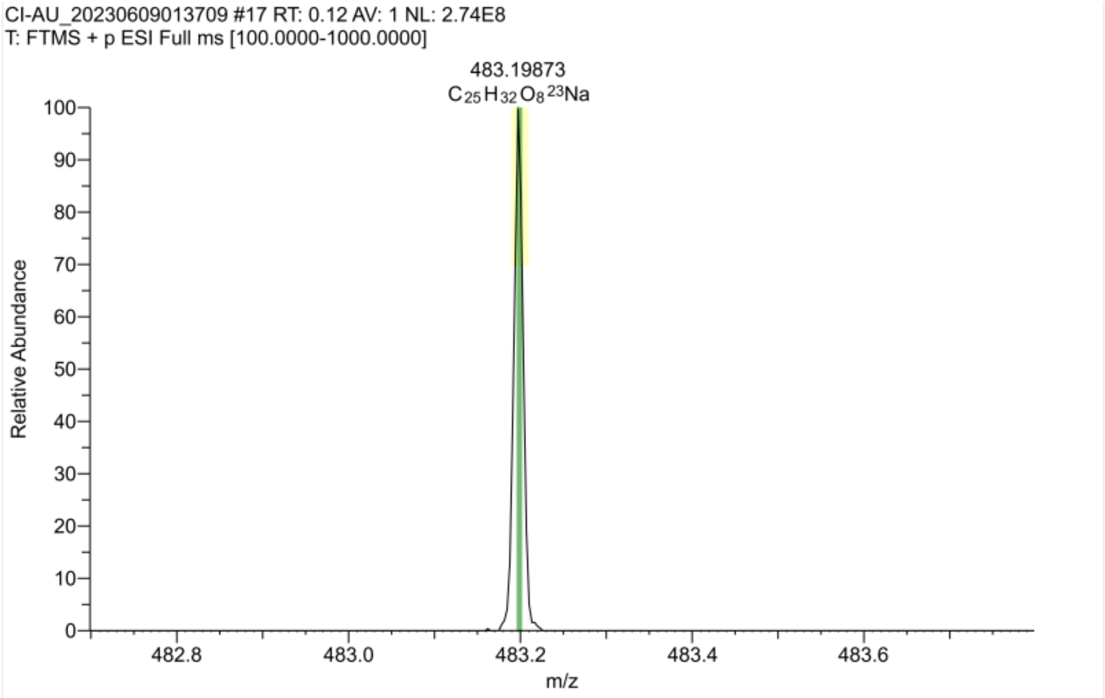

Peak Mass	Display Formula	S Fit	RDB	Delta [ppm]	Theo. mass	Pattern Cov. [%]	MSMS Matched...
483.19873	$C_{25}H_{32}O_8{}^{23}Na$	29.0568836020822	9.50	-0.43	483.19894	95.57	(Collection)

Figure S9. HRESIMS spectrum of **1**

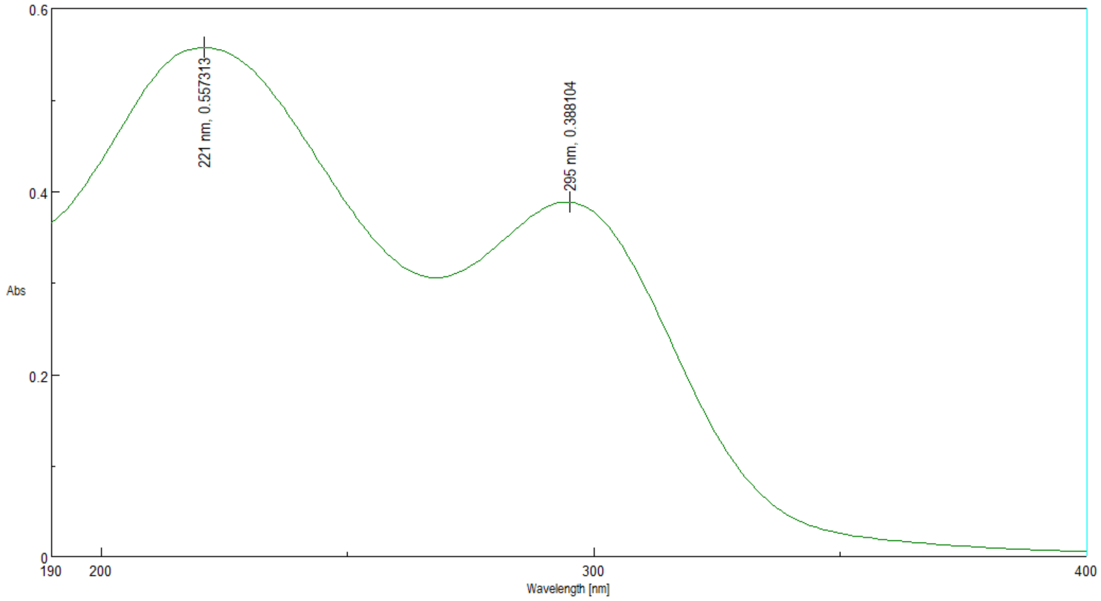

Figure S10. UV spectrum of **1**

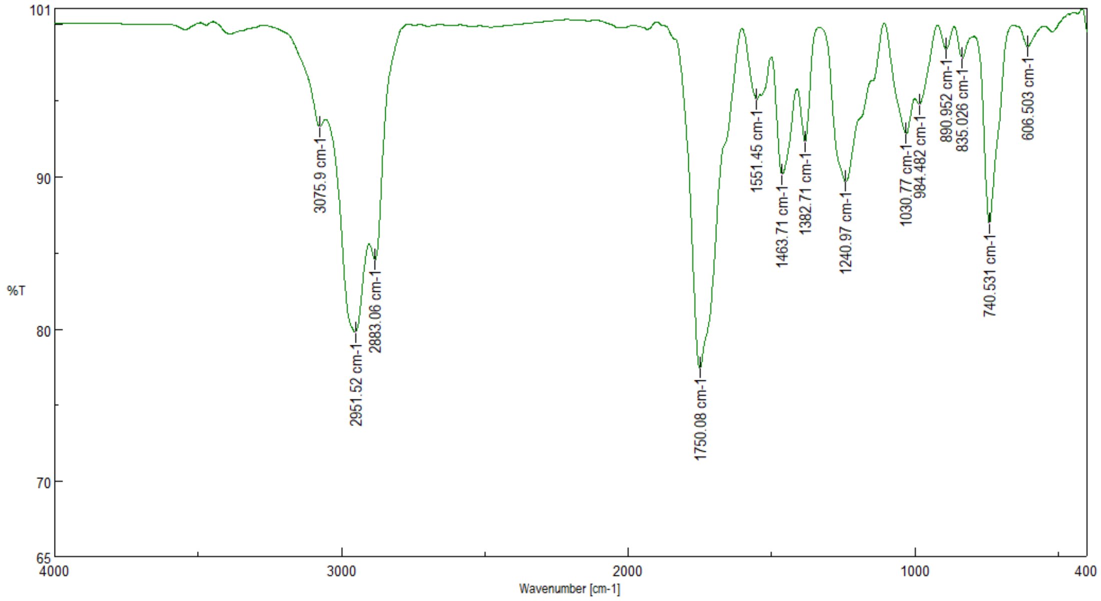

Figure S11. IR spectrum of **1**

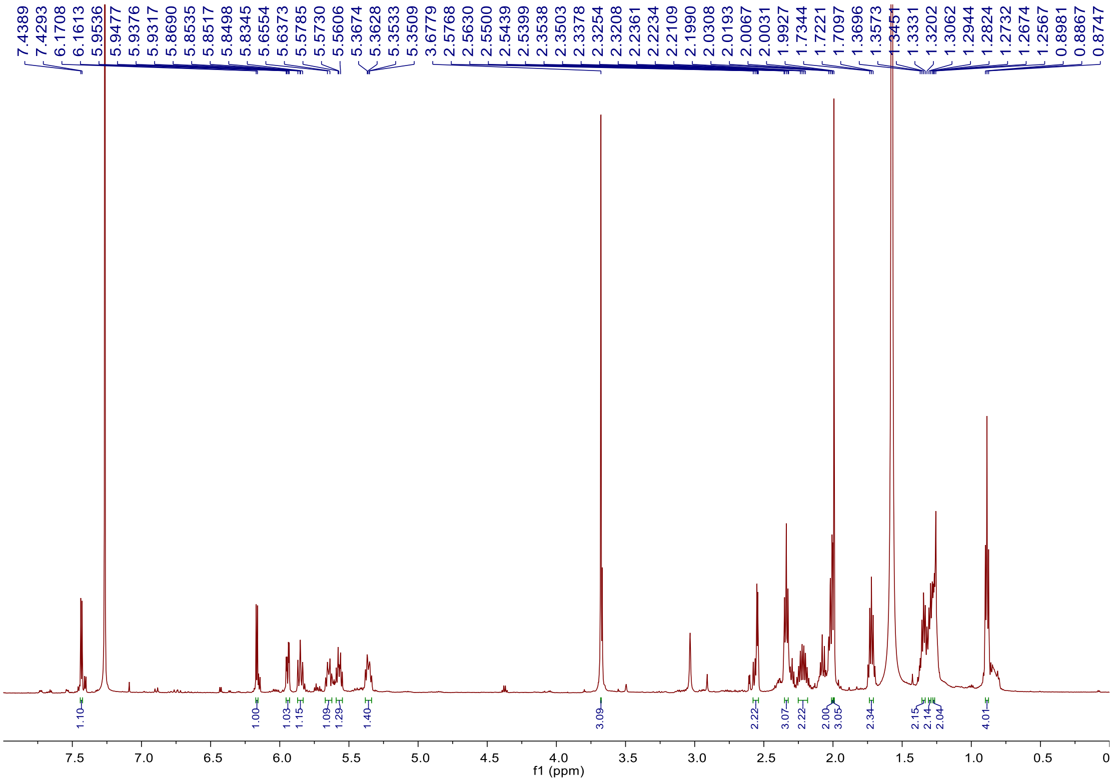

Figure S12. ^{1}H NMR spectrum of **2** (600 MHz, $CDCl_3$)

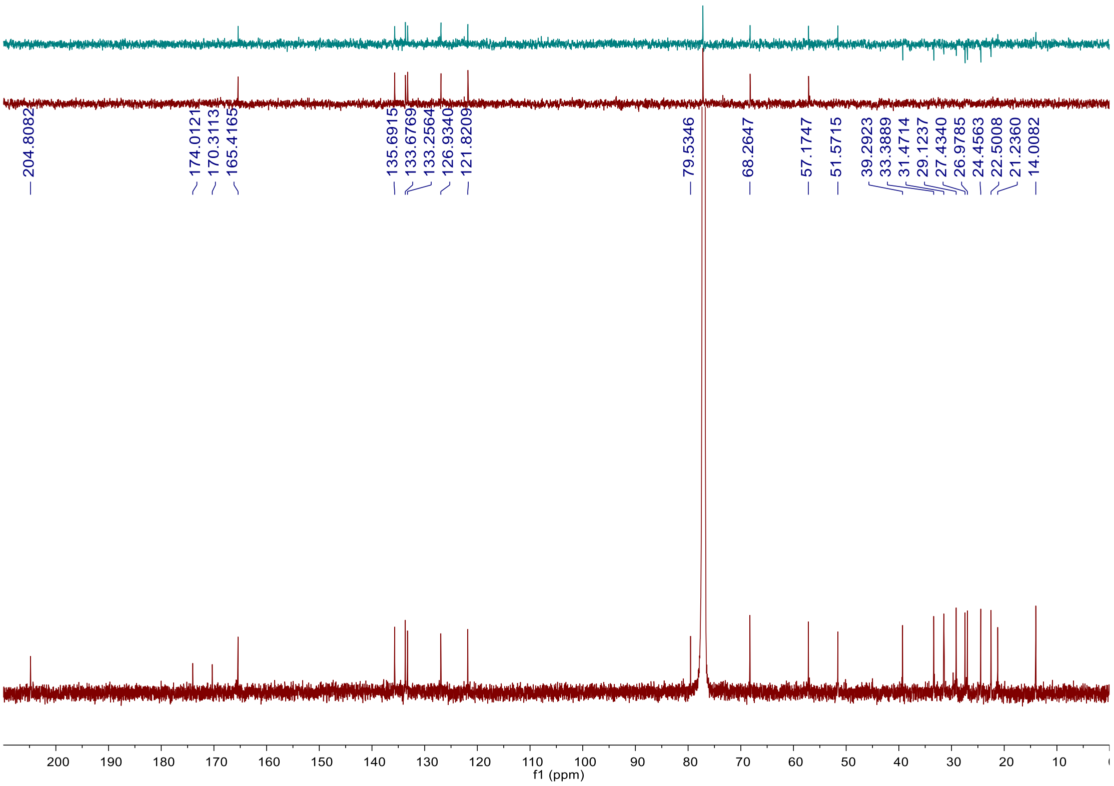

Figure S13. ^{13}C NMR spectrum of **2** (125 MHz, $CDCl_3$)

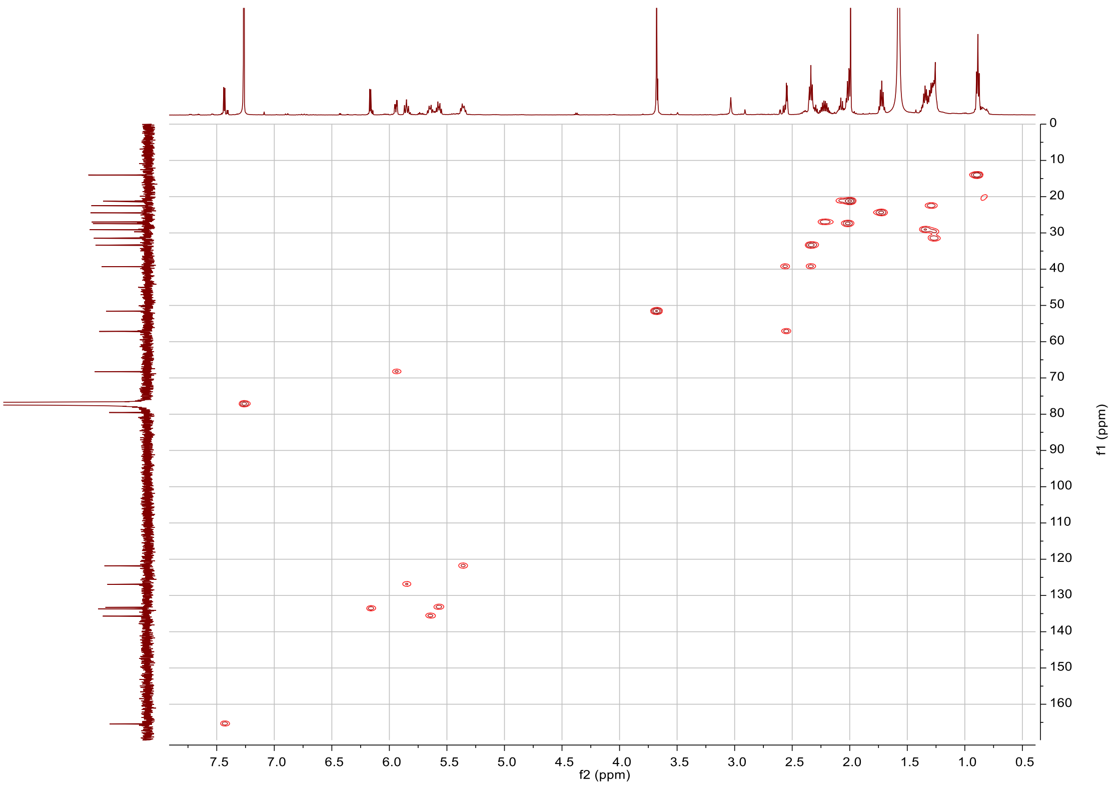

Figure S14. COSY spectrum of **2**

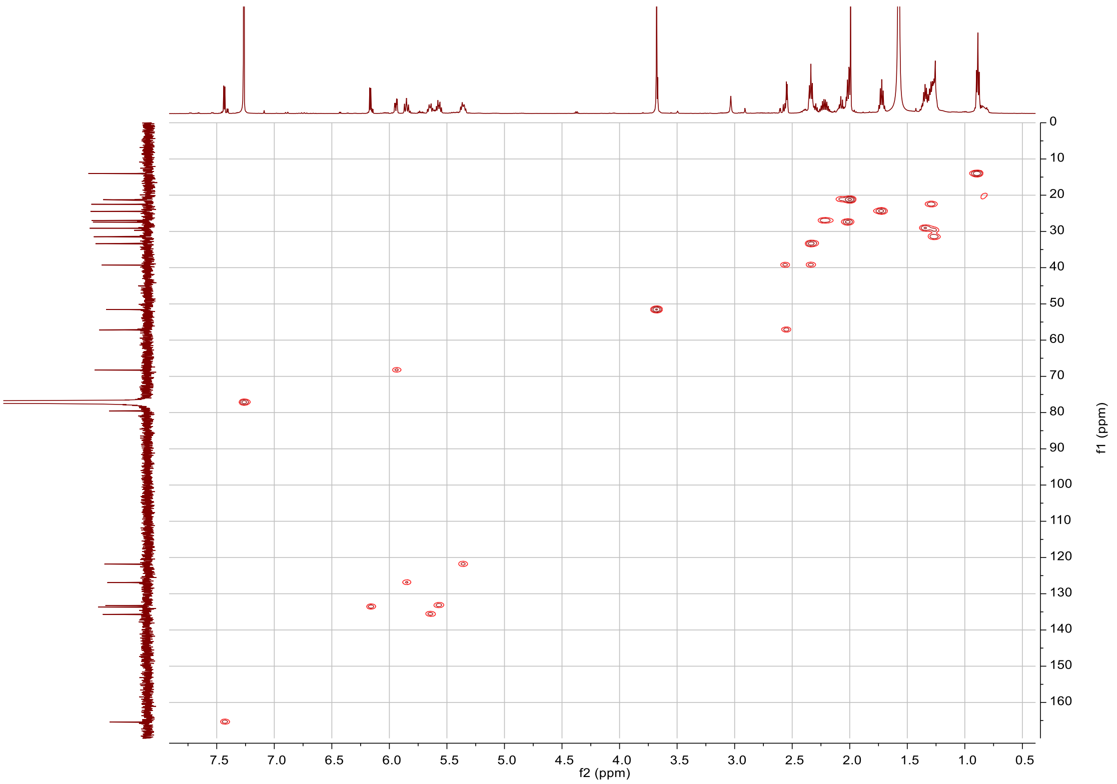

Figure S15. HSQC spectrum of **2**

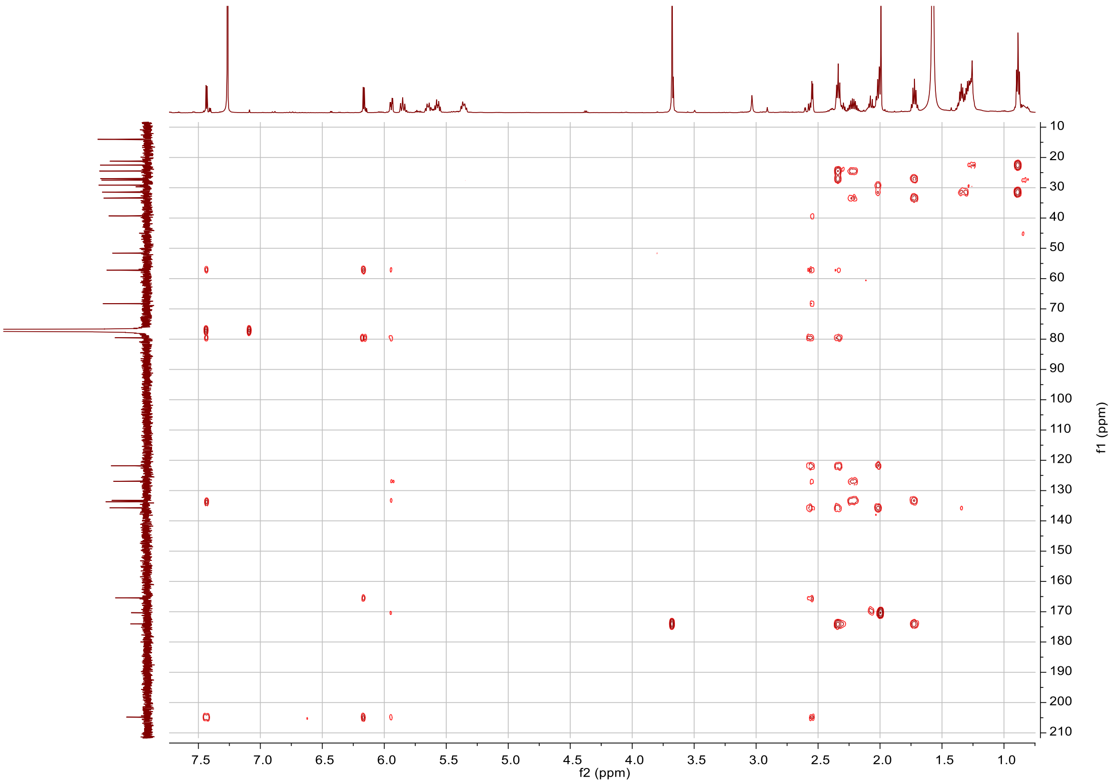

Figure S16. HMBC spectrum of **2**

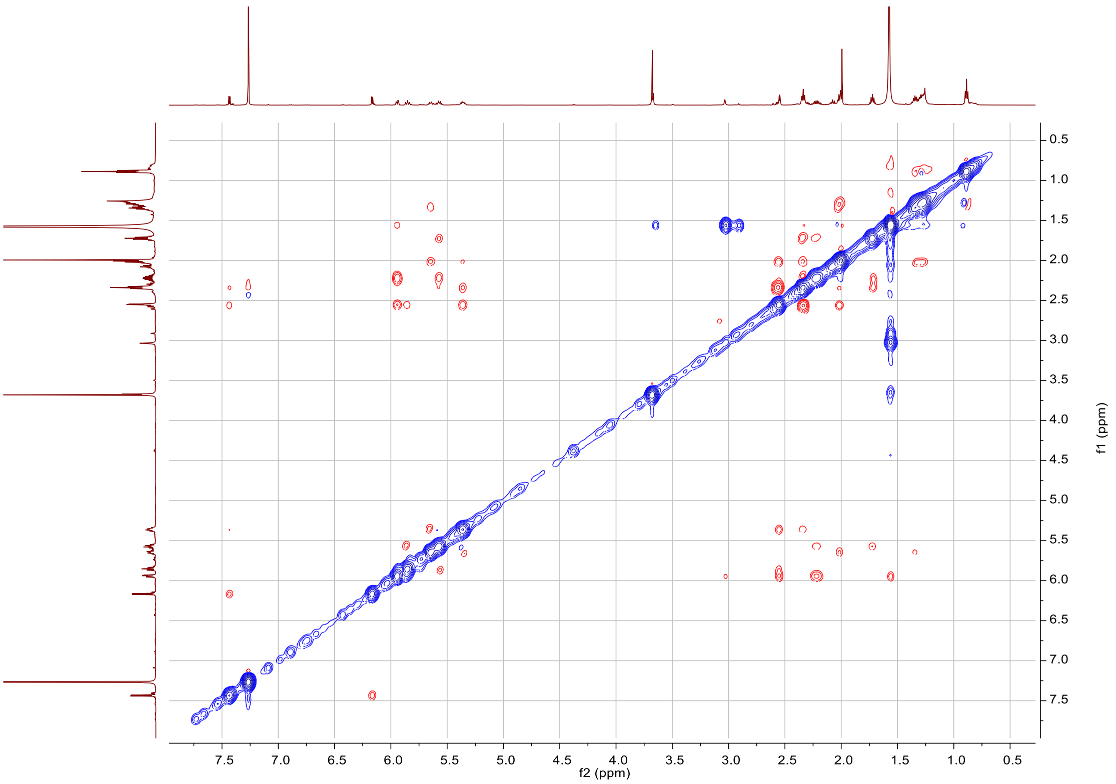

Figure S17. NOESY spectrum of **2**

Mass Spectrum SmartFormula Report

Analysis Info

Analysis Name	D:\1107\CIACR.d	11/8/2022 2:45:15 PM
Method	tune_wide_pos_20220422.m	Operator: YU HSIAO-CHING
Sample Name	CI-AC	Instrument: BRUKER micrOTOF-Q
Comment	ESI Positive	

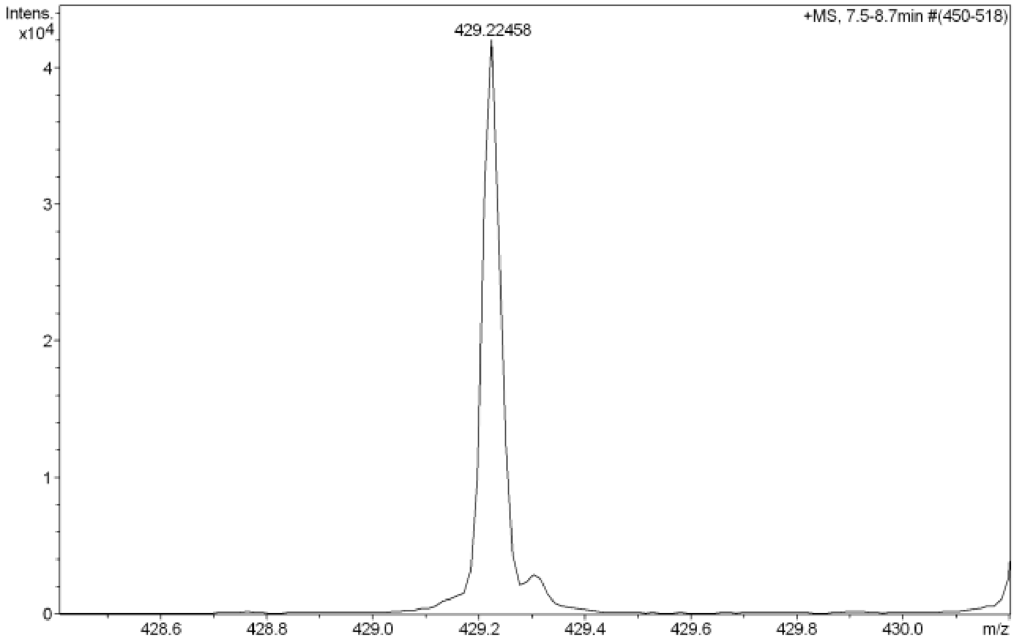

Meas. m/z	#	Formula	Score	m/z	err [mDa]	err [ppm]	mSigma	rdb	e⁻ Conf	N-Rule
429.22458	1	C 23 H 34 Na O 6	100.00	429.22476	0.18	0.42	37.8	6.5	even	ok

Figure S18. HRESIMS spectrum of **2**

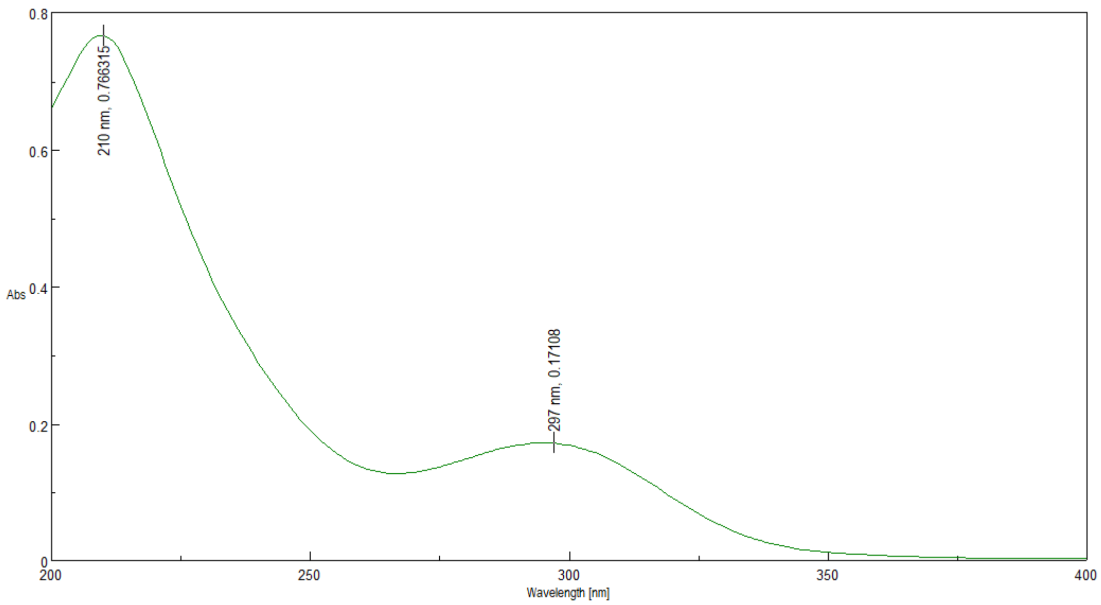

Figure S19. UV spectrum of **2**

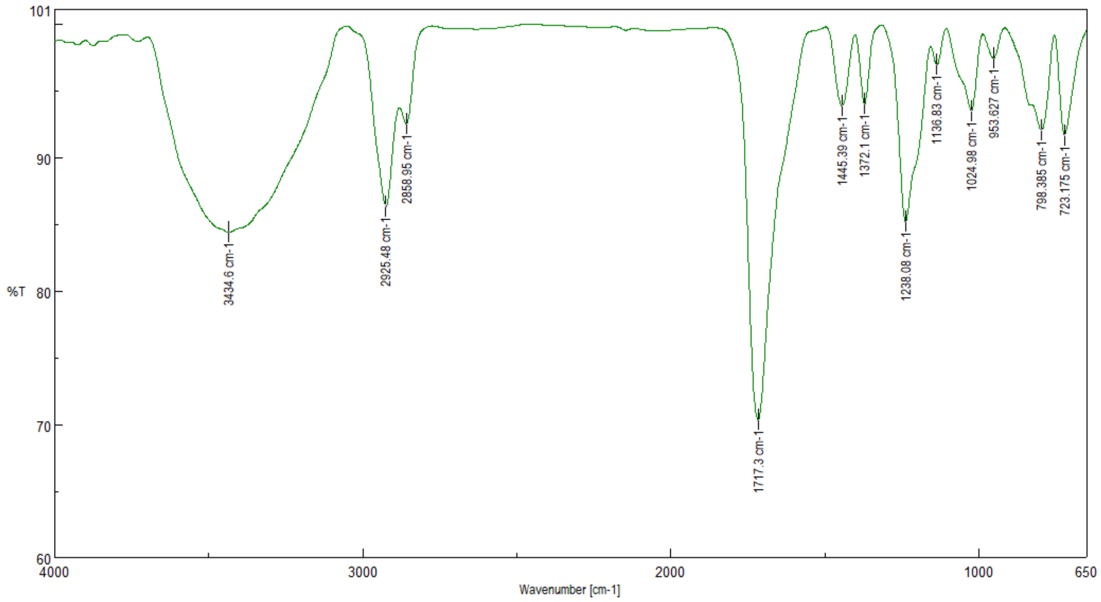

Figure S20. IR spectrum of **2**
